# Three-Component Synthesis of 1-Substituted 5-Aminotetrazoles Promoted by Bismuth Nitrate

**DOI:** 10.1021/acs.joc.4c01727

**Published:** 2024-09-13

**Authors:** Iva S. de Jesus, Amenson Trindade Gomes, Igor Sande, Silvio Cunha

**Affiliations:** † Instituto de Química, Universidade Federal da Bahia, Campus de Ondina, Salvador, Bahia 40170-115, Brazil; ‡ Instituto Nacional de Ciência e Tecnologia - INCT em Energia e Ambiente, Campus Ondina, Salvador, Bahia 40170-290, Brazil

## Abstract

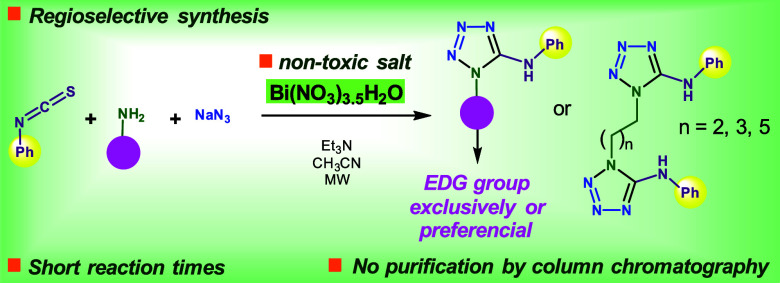

A nontoxic bismuth-promoted multicomponent synthesis of 5-aminotetrazoles and bistetrazoles is reported. The reaction of phenyl isothiocyanate, NaN_3_, and amine (primary aliphatic, aromatic, and aliphatic diamine) promoted by Bi(NO_3_)_3_·5H_2_O under microwave heating affords good yields, short reaction times, simple workup, and purification without column chromatography. A set of diagnostic ^1^H NMR signals was identified as a guide for quickly elucidating the exclusive (or main) regioisomer formed, with the stronger electron donor group located at heterocyclic nitrogen 1. This regioselectivity is strongly dependent on the electronic density of the amine. It is opposite to that obtained by several thiourea desulfurization methods promoted by thiophilic metals and metal-free protocols.

## Introduction

**Figure 1:**
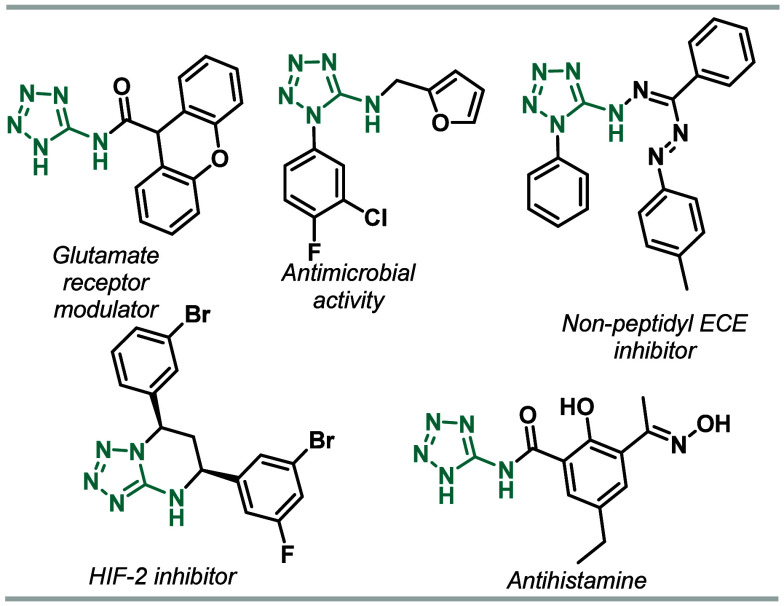
Drugs and biologically active compounds containing the 5-aminotetrazole moiety.

Tetrazoles are poly aza-heterocyclic compounds consisting of a five-membered ring of four N atoms and one C atom,^1,2^ which are scarce in nature.^3^ In particular, 5-aminotetrazoles are found in compounds that present antiviral,^4^ antineoplastic,^5^ antiallergic/antiasthmatic,^6^ antihistaminic,^7^ antimicrobial activities,^8^ have a selective positive allosteric modulator of mGlu1 receptors,^9^ and are used in the treatment of cognitive diseases.^10^ They also have an inhibitory effect on the secretion process of hepatitis B virus surface antigen (HBsAg)^11^ and could act as nonpeptidyl ECE inhibitors^12^ and HIF-2,^13^ as shown in Figure 1. Additionally, 5-aminotetrazoles exhibit a wide range of applications such as in bioorganic chemistry,^14^ coordination chemistry,^15^ and materials science, including photography^16^ and explosives.^17^ These compounds are also valuable building blocks for the preparation of other nitrogen-containing heterocycles.^18^

**Scheme 1:**
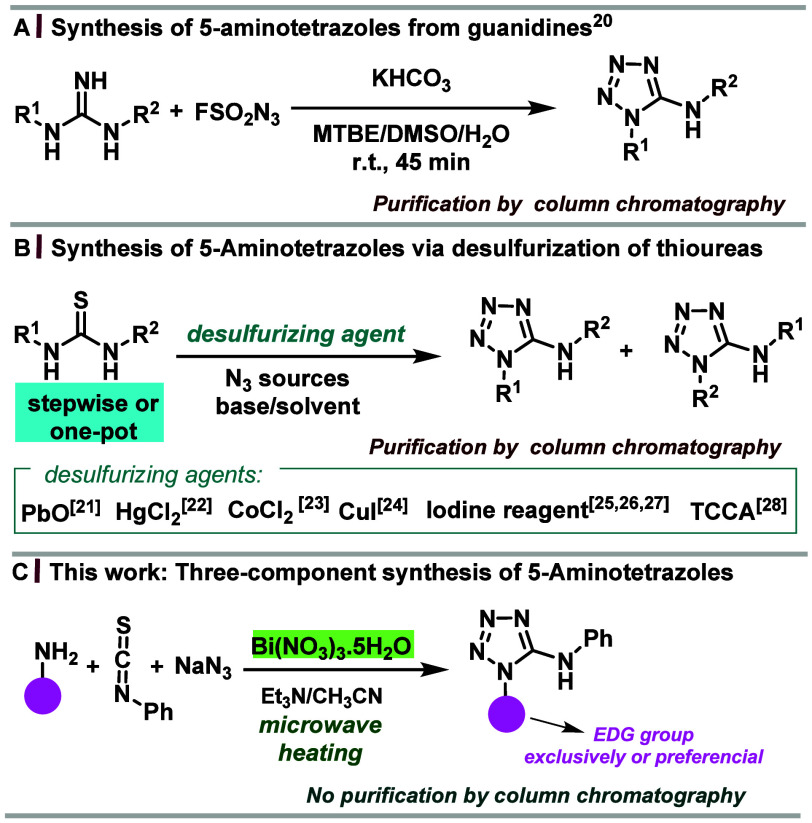
Synthetic Routes to 1,5-Disubstituted 5-Aminotetrazoles

Due to these diverse applications, several methods describing the synthesis of 5-aminotetrazoles have been reported.^19,20,21,22,23,24,25,26,27,28^ Despite there being different combinations of nitrogen substrates (for instance, thiourea^22^ and guanidine^20^) for the synthesis of 5-aminotetrazoles, the most common route for preparing these compounds is the addition of the azide ion to substituted thiourea promoted by thiophiles such as PbO,^21^ HgCl_2_,^22^ CoCl_2_,^23^ copper(I) salts,^24^ iodine,^25^*o*-iodoxybenzoic acid (IBX),^26^ iodobenzene/oxone,^27^ and trichloroisocyanuric acid^28^ (Scheme 1). These metal-based and metal-free methods are traditional stepwise approaches whose purification requires column chromatography. In this context, alternatives that dispense toxic desulfurizing agents and can yield 1-substituted 5-aminotetrazoles in a multicomponent strategy with simple purification are a goal yet to be achieved.

Multicomponent reactions (MCRs) have garnered extensive application as efficient strategies for constructing structurally intricate scaffolds possessing pharmacological significance.^29^ Multicomponent synthesis of 1,5-disubstituted tetrazoles is a theme of ongoing interest, and the elegant Ugi-tetrazole four-component reaction is the most versatile example.^30^ However, it does not afford 1,5-disubstituted 5-aminotetrazoles.

In recent years, bismuth salts have emerged as a powerful alternative for the catalysis or promotion of various organic transformations due to their Lewis acidity.^31^ Additionally, most bismuth compounds are inexpensive, relatively nontoxic, readily available, eco-friendly, and easy to handle.^32^ Our previous studies on the use of bismuth salts as desulfurizing agents in guanylation reactions^33,34^ prompted us to develop a bismuth-promoted three-component synthesis of 1-substituted 5-aminotetrazoles and bistetrazoles under microwave heating (Scheme 1c), avoiding purification by column chromatography.

## Results and Discussion

For the synthesis of substituted 5-aminotetrazoles, we were inspired by our previous studies concerning the use of bismuth salts as thiophiles in bicomponent guanylation reaction.^34^ Thus, easily prepared *N*,*N*′-disubstituted thioureas and NaN_3_ were envisioned as versatile reagents for the bismuth-promoted synthesis of tetrazoles. 1-Benzyl-3-phenylthiourea **1a** was chosen as a model substrate, and different reaction parameters were investigated (Table 1). Initially, **1a** was reacted in CH_3_CN in the presence of NaN_3_**2** and Bi(NO_3_)_3_·5H_2_O at room temperature (entry 1), with reagents being recovered. Under reflux, a slow reaction (24 h) was observed, affording 5-aminotetrazoles **3a** as a single isomer (entry 2), revealing that bismuth nitrate is effective as thiophilic Lewis acid for the stepwise synthesis of this class of tetrazoles from thiourea and NaN_3_.

**Table 1:**
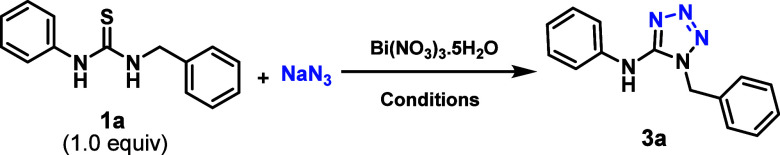
Optimization of the Bicomponent Reaction Conditions

Entry	NaN_3_ equiv	Bi(NO_3_)_3_ equiv	Solvent	Temp (°C)	Time (min)	Base^a^	Yield (%)^b^
1	3	1	CH_3_CN	rt	1440	Et_3_N	trace
2	3	1	CH_3_CN	reflux	1440	Et_3_N	59
3	3	1	CH_3_CN	125^c^	20	Et_3_N	72
4	2	1	CH_3_CN	125^c^	20	Et_3_N	36
5	1	1	CH_3_CN	125^c^	20	Et_3_N	trace
6	3	0.5	CH_3_CN	125^c^	20	Et_3_N	13
7	3	1	H_2_O	125^c^	20	Et_3_N	none^d^
8	3	1	H_2_O	125^c^	20	K_2_CO_3_	none^d^
9	3	1	DMF	125^c^	15	Et_3_N	69

a 3.0 equiv.

b Isolated yield.

c Microwave heating.

d Urea formed.

The same reaction was investigated under microwave heating (MW), and **3a** was obtained in 20 min (a significant reaction time decrease of 99%, entry 3), which places MW conditions as crucial to achieving an efficient transformation with nontoxic Bi(NO_3_)_3_·5H_2_O. All metal-based methods of 5-aminotetrazole synthesis from thioureas employ three equivalents of NaN_3_ per equivalent of organic substrate, and this proportion was the starting point (entries 1–3). However, the amount of NaN_3_ was also studied, and a dramatic yield decrease was observed when lower amounts of NaN_3_ were employed (entries 4–5). In addition, when the Bi(NO_3_)_3_·5H_2_O load was reduced to 0.5 equiv, the isolated yield was unsatisfactory (entry 6). Additional optimization studies (using BI_3_ and other MW conditions) can be found in the Supporting Information.

**Scheme 2:**
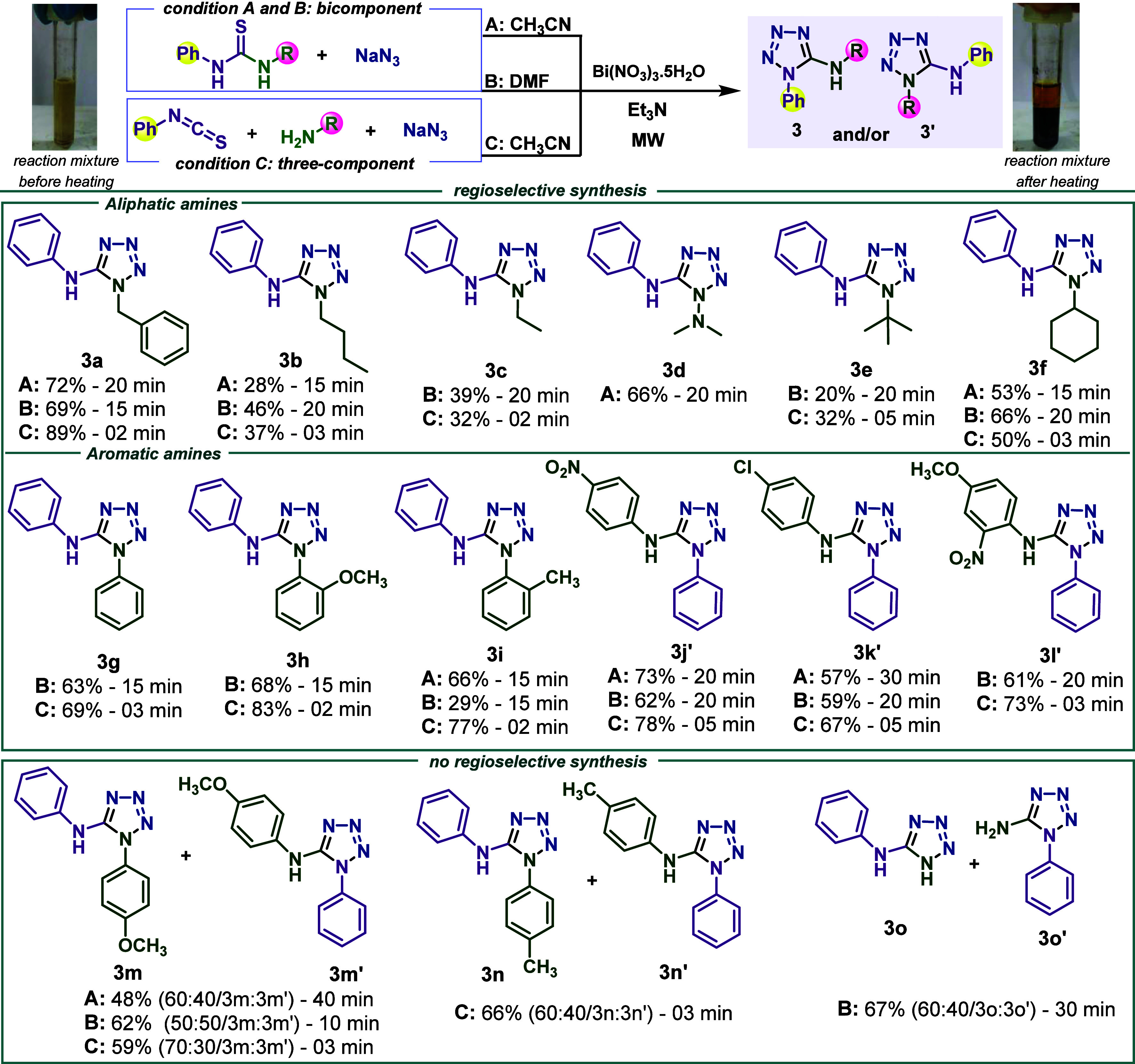
Bi- and Three-Component Bismuth-Promoted 5-Aminotetrazoles Synthesis under Microwave Heating Reaction conditions A and B: All reactions (1.0 mmol scale) were performed using a 1:3 ratio of thiourea and NaN_3_, 1.0 mmol Bi(NO_3_)_3_·5H_2_O, 5.0 mL of CH_3_CN (for condition A) or DMF (for condition B), 10–40 min, 125°C, 150 W. The crude product is recrystallized by ethanol. Reaction condition C: All reactions (1.0 mmol scale) were performed using a 1:1:3 ratio of phenyl isothiocyanate, amine, and NaN_3_, 1.0 mmol Bi(NO_3_)_3_·5H_2_O, 5.0 mL of CH_3_CN, 2–5 min, 125 °C, 150 W. The crude product is recrystallized by ethanol.

In a reported telescopic synthesis of 5-aminotetrazoles in water as the solvent, HgCl_2_-promoted desulfurization of thioureas afforded satisfactory yields.^22b^ A similar condition was herein tested with the nontoxic Bi(NO_3_)_3_·5H_2_O. When the model reaction was evaluated in water, the corresponding urea of **1a** was exclusively formed in 86% and 81% yield (entries 7 and 8 of Table 1, respectively, compound **1a′** with experimental details in the Supporting Information). Thus, among the tested solvents (entries 1–9), CH_3_CN was the optimal choice, affording a 72% yield of the desired product. Despite DMF affording **3a** in comparable yield to CH_3_CN and somewhat lower reaction time (see entry 9 of Table 1 and Scheme 2 for other examples), the fast and straightforward workup of the reaction mixture avoiding tedious DMF elimination (see Experimental Section) confers better performance to CH_3_CN as a solvent in this desulfurization, in terms of time consumption and waste generation.

With the optimal conditions in hand, the substrate scope was evaluated with a set of 1,3-disubstituted thioureas **1**, and results are presented in condition A of Scheme 2. However, some thioureas were insoluble in CH_3_CN even under heating, which forced us to synthesize tetrazoles **3d**, **3g**, **3h**, **3j′**, **3l′**, and **3o–3o′** in DMF, condition B of Scheme 2. Thioureas with one N-aliphatic substituent were successfully employed, affording 5-aminotetrazoles **3a**–**f** in low to modest yields (20–69%). *N*-aromatic thioureas bearing electron-donating substituents, *o*-OMe (**3h**), *o*-Me (**3i**), *p*-OMe (**3n** and **3n′**), and electron-withdrawing groups, *p*-NO_2_ (**3j′**), *p*-Cl (**3k′**), and *o*-NO_2_-*p*-OCH_3_–Ph (**3l′**), afforded the corresponding 5-aminotetrazoles in moderate yields (59–68%, Scheme 2).

Due to our interest in multicomponent reactions,^35^ we envisioned a three-component synthesis of 5-aminotetrazoles through the combination of different amines, phenyl isothiocyanate, and NaN_3_, in the presence of Bi(NO_3_)_3_·5H_2_O under microwave heating. At the onset of our study, benzylamine was chosen as the model substrate to investigate the feasibility of this three-component reaction. The three-component reaction afforded pure product **3a** in 89% yield and a reaction time of 2 min, which was isolated by simple filtration, avoiding purification by column chromatography. Encouraged by these findings, the scope regarding the amine component was reevaluated (Scheme 2). The reaction time was low for all of the aliphatic amines tested (condition C, Scheme 2), and the yields were higher than those obtained for the bicomponent reaction (condition A or B). An additional advantage of the three-component synthesis is that all reagents are soluble in CH_3_CN, which provides an operationally simple workup for the synthesis of 5-aminotetrazoles.

Overall, the yields obtained in the three-component reaction were higher than those obtained from the preformed thiourea for all aliphatic and aromatic amines. Aromatic amines containing an electron-withdrawing group (EWG) afforded tetrazoles in reaction times that were higher than those of aromatic amines containing an electron-donating group (EDG). A possible explanation for the remarkably decreased reaction time observed for the three-component method is a profile of pseudo-first order reactions under this condition because, at the initial time, the initial concentration of the azide is much higher than the initial concentration of *in situ* formed thiourea. Thus, the reaction rate depends only on the concentration of the thiourea, which, when formed, is immediately consumed by the nucleophilic azide.

**Scheme 3:**
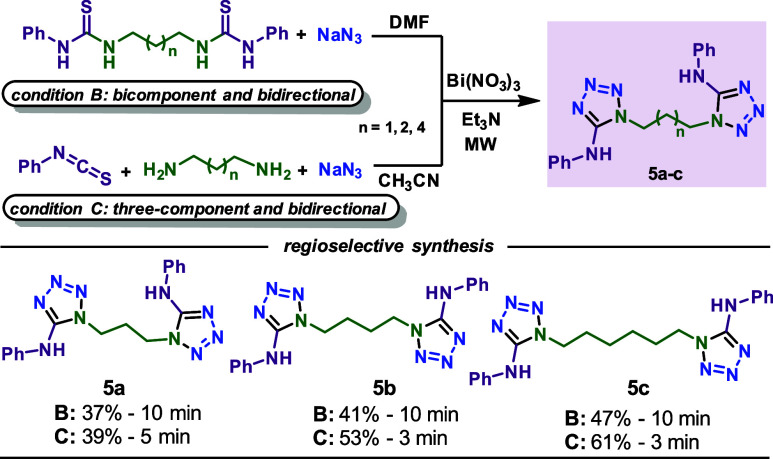
Bismuth-Promoted Bidirectional Multicomponent Synthesis of Bis-5-Aminotetrazoles Condition B: reactions were performed using 1:6:2 ratio of bisthiourea, NaN_3_, and Bi(NO_3_)_3_·5H_2_O, 5 mL of DMF, 125 °C. Condition B: performed using 2:1:6:2 ratio of phenyl isothiocyanate, amine, NaN_3_, and Bi(NO_3_)_3_·5H_2_O, 5 mL of CH_3_CN, 2–5 min, 125 °C, 150 W. The crude product is recrystallized by ethanol.

After various 1,5-disubstituted 5-aminotetrazoles, the bismuth-promoted protocol was extended to bis-5-aminotetrazoles from previously synthesized or in *situ* prepared bisthioureas **4**. Thus, aliphatic primary diamines with four different spacers were tested, as shown in Scheme 3. While reagents were recovered with ethylenediamine, products **5a**–**c** were isolated in good yields in 3–10 min of reaction time. In both stepwise and three-component reactions, greater yields were observed with increasing chain size. Based on these results, the three-component reaction was the best strategy under the conditions studied for synthesizing mono and bis-1,5-disubstituted 5-aminotetrazoles.

**Figure 2:**
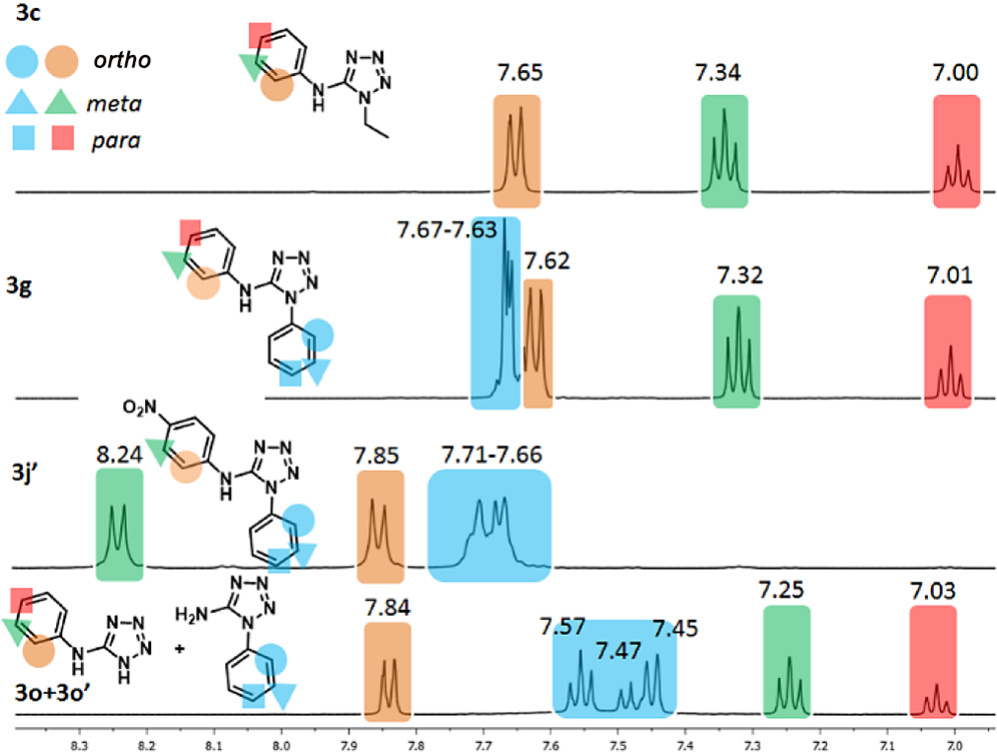
Comparison of ^1^H NMR spectra (DMSO-*d*_6_, 500 MHz) of representative 5-aminotetrazoles (**3c**, **3g**, **3j′** and **3o** + **3o′**) showed different resolution patterns for aromatic hydrogens at position N1 (overlapped) and N5 (well resolved).

Differentiation between regioisomeric 1,5-diaryl-5-aminotetrazoles is unequivocally done by X-ray structural determination.^19,22c,24,25,36^ Although most 5-aminotetrazoles are solid compounds, the dependence of monocrystal obtention is a severe restriction to the elucidation of which aryl group is at the endocyclic (N1) or exocyclic (N5) position. Comparative studies on the NMR data are rarely explored to distinguish between regioisomers of nonsymmetrical 1,5-disubstituted 5-aminotetrazoles.^19,19^ However, two reports indicated that NMR data is adequate to distinguish between monosubstituted regioisomeric 1-phenyl-5-aminotetrazole/1-*H*-5-aminophenyltetrazole, and 1,5-diaryl-5-aminotetrazoles because, in the ^1^H NMR spectra of such derivatives, the *ortho*, *meta*, and *para* hydrogen signals are well resolved at the N5-aryl group. In contrast, the same signals of the hydrogens at N1-aryl are overlapped. To evaluate if these general trends are also adequate for differentiation of nonsymmetrical 1,5-disubstituted 5-aminotetrazoles, we compared ^1^H NMR spectra of selected 1,5-disubstituted 5-aminotetrazoles **3c**, **3g**, **3j′** and the mixture of isomers **3o**/**3o′**, as indicated in Figure 2.

Compound **3c** was chosen as the model because it is easily characterized as N5-aryl substituted by the ^1^H NMR, and the mixture of isomers **3o**/**3o′** presented the most resolved set of signals at N1-aryl. As can be seen in compound **3g** with identical phenyl groups at N1 and N5, chemical shifts for the aromatic hydrogens at N5 are similar to those in **3c** and **3g**. In contrast, hydrogens at N1-phenyl are not resolved, with each hydrogen signal attributed to the specific phenyl group at N1 or N5. These NMR features are also observed in nonsymmetrical substituted derivative **3j′** (and other nonsymmetrical derivatives, see Supporting Information). They are enough to distinguish between regioisomers of nonsymmetrical derivatives, even in the case of derivatives **3j′** and **3o′** where hydrogens at N1 are totally overlapped, as in **3g**. As a general guide for discrimination between similar 1,5-diaryl-5-aminotetrazoles with different aryl groups at N1 and N5, the hydrogens of the aryl group at N1 generate a second-order multiplet. In contrast, the hydrogens of the aryl group at N5 lead to a first-order splitting pattern.

Structural analysis of all obtained 1,5-disubstituted-5-aminotetrazoles from phenyl isothiocyanate revealed that the regioselectivity herein synthesized is strongly determined by the electronic density of the employed amine, whereby one tetrazole regioisomer is exclusively formed (or is the major isomer) with the stronger EDG located at heterocyclic N1 and the EWG at exocyclic nitrogen (Scheme 2). This regioselectivity is opposite to that obtained by thioureas/NaN_3_ and other metal salts such as HgCl_2_^22^ and Cu salts,^24^ and from various non-metal based syntheses of 1,5-disubstituted-5-aminotetrazoles.^19h,19e,25,26,27^

**Scheme 4:**
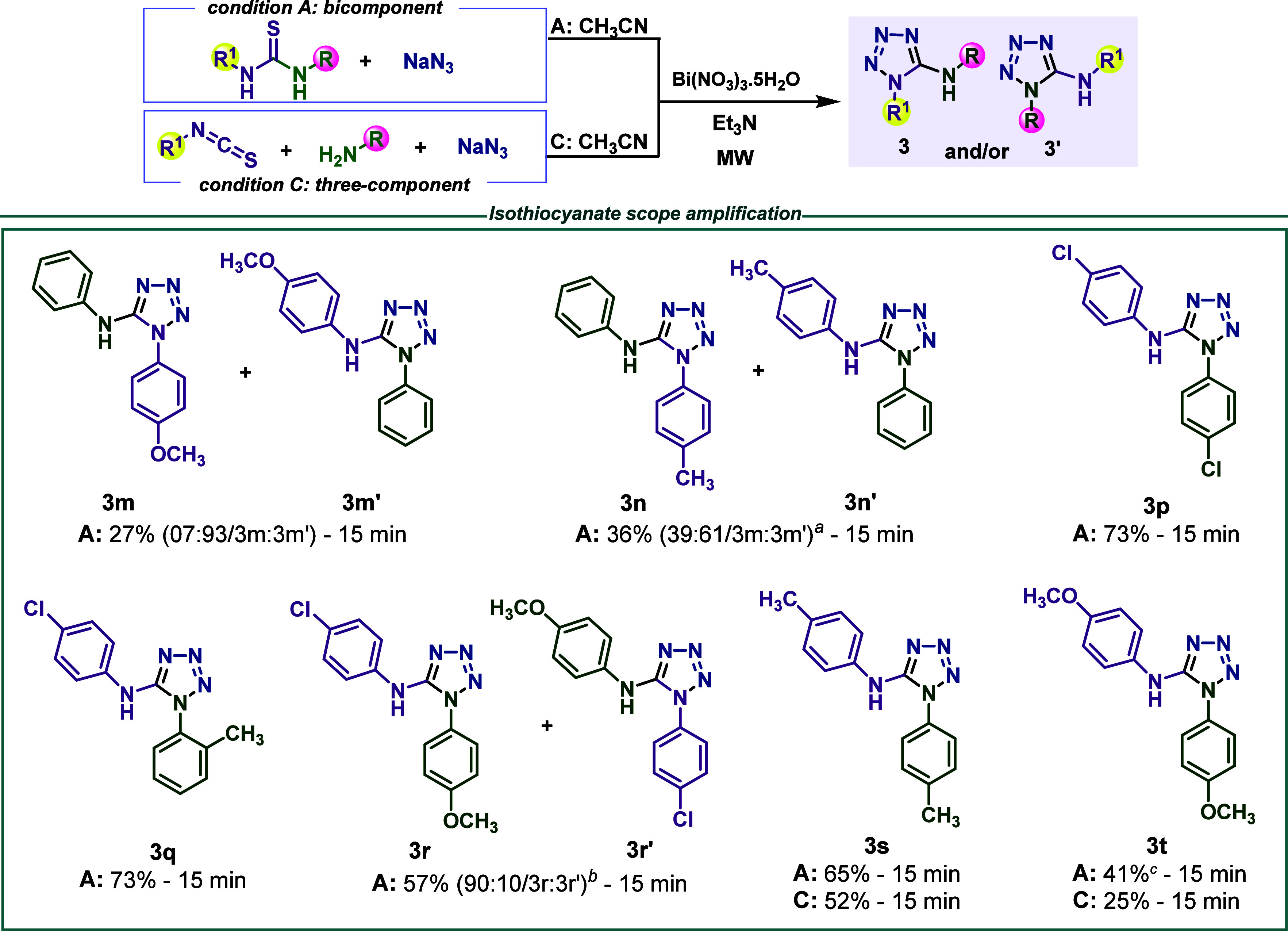
Reaction Scope for Selected Substituted Isothiocyanates Reaction condition A: All reactions (0.5 mmol scale) were performed using a 1:3 ratio of thiourea and NaN_3_, 0.5 mmol Bi(NO_3_)_3_·5H_2_O, 2.5 mL of CH_3_CN, 15 min, 125 °C, 150 W. The crude product is recrystallized by ethanol/water. Reaction condition C: All reactions (0.5 mmol scale) were performed using a 1:1:3 ratio of isothiocyanate, amine, and NaN_3_, 0.5 mmol Bi(NO_3_)_3_·5H_2_O, 2.5 mL of CH_3_CN, 15 min, 125 °C, 150 W. Contamined with 7% of 1-(4-methylphenyl)-3-phenylurea (**6a**), quantified by ^1^H NMR. Contamined with 11% of 1-(4-chlorophenyl)-3-(4-methoxyphenyl)urea (**6b**), quantified by ^1^H NMR. Contamined with 11% of 1,3-bis(4-methoxyphenyl)urea (**6c**), quantified by ^1^H NMR.

Three additional isothiocyanates were selected to amplify the reaction scope, and the results are presented in Scheme 4: *p*-CH_3_O–Ph, *p*-CH_3_–Ph, and *p*-Cl–Ph isothiocyanates. Under condition C of Scheme 4, a complex mixture of compounds in the reaction of anilines with *p*-Cl–Ph isothiocyanate was observed, despite the fact that the corresponding tetrazol was detected by TLC analysis. With the other two isothiocyanates, the tetrazoles **3s** and **3t** could be obtained in low yield (Scheme 4). However, all isothiocyanates were successfully transformed into thioureas under bicomponent conditions and tetrazoles **3m**–**3t** were obtained. Some of these 1,5-disubstituted-5-aminotetrazoles were previously prepared (Scheme 2) as regioisomeric mixtures (**3m/3m′** and **3n/3n′**). The additional tetrazoles **3p**–**3t** were obtained, with the nonsymmetrical **3q** and **3r/3r′** being formed with the EDG group at endocyclic nitrogen (Scheme 4).

**Scheme 5:**
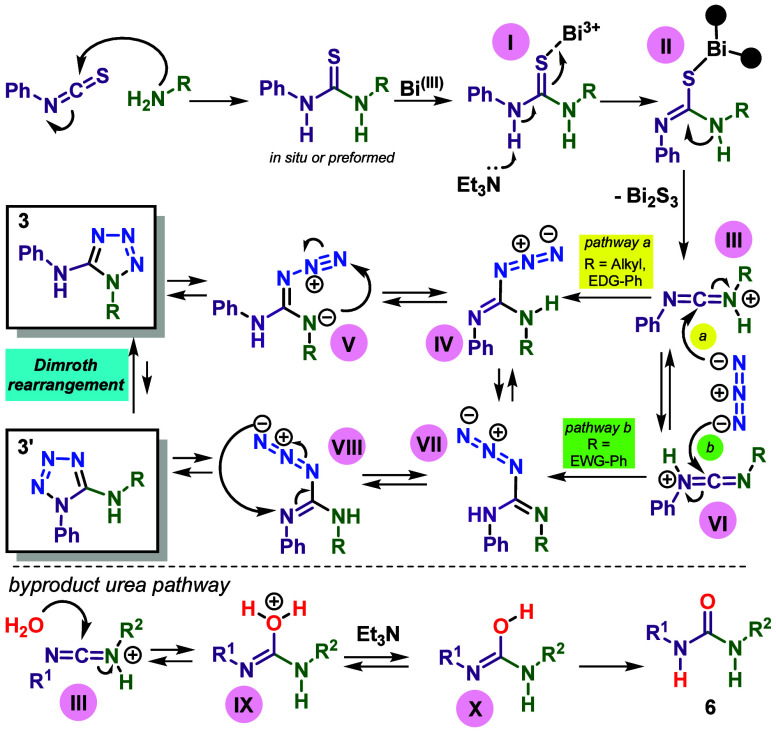
Proposed Mechanism for Bi(NO_3_)_3_-Promoted Formation of 1-Substituted 5-Aminotetrazoles from *N*,*N*′-Disubstituted Thioureas

To **3m/3m′** previously obtained via the bicomponent approach, a shorter reaction time was applied to gain insight into the effect of reaction time on the regioisomeric proportion. Thus, when the reaction time was decreased from 40 min (condition A, Scheme 2) to 15 min (condition A, Scheme 4), isomer **3m′** was the major one, which indicates that reaction time is an important variable in the regioisomer distribution of the 1-substituted 5-aminotetrazoles synthesis promoted by bismuth nitrate. This fact will be discussed below in the context of the proposed mechanism of 1-substituted 5-aminotetrazoles formation from *N*,*N*′-disubstituted thioureas (Scheme 5).

A mechanistic proposal was formulated in Scheme 5 to rationalize this regioselectivity. The metal coordinates with the thiourea sulfur atom in all metal-based mechanistic pathways to 5-aminotetrazoles.^19a,19g,22,23,24^ Similarly, Bi(III) should behave as a thiophilic soft Lewis acid that coordinated with thiourea (species **I** and **II** in Scheme 5) to promote base-induced desulfurization forming an intermediate carbodiimide (**III/VI**),^34^ which reacts with an azide ion affording a guanylazide (**IV**/**VII**) as a common advanced intermediate. In the next step, intramolecular cyclization/electrocyclization results in the tetrazole ring (Scheme 5). Protonated carbodiimide **III** is more stable than **VI** when an alkyl or EDG-aryl group is attached to the nitrogen atom from the amine and, upon tautomeric equilibrium, the more basic R–N nitrogen of **V** attacks terminal N of guanylazide **V** to form tetrazole ring **3**. Otherwise, more stable protonated carbodiimide **VI** from EWD-arylamine affords isomeric tetrazole **3′**. The formation of byproduct urea can be rationalized by the addition reaction of H_2_O with protonated carbodiimide **III** (or **IV**), affording transient **IX**, which, after reaction with Et_3_N, **X** tautomerizes to stable urea **6** (Scheme 5).

**Scheme 6:**
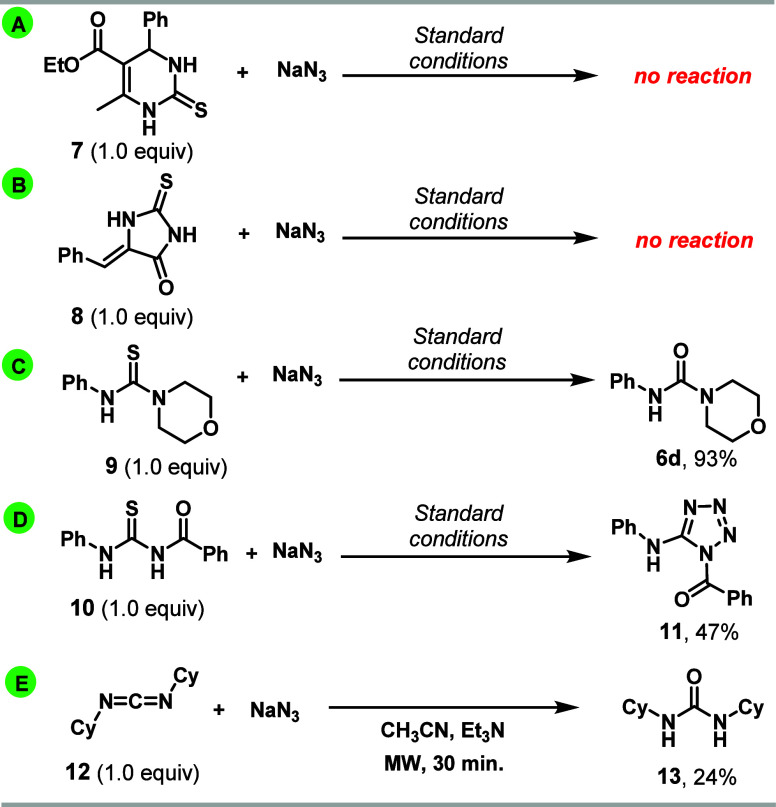
Control Experiments All reactions (1.0 mmol scale) were performed using 3.0 mmol NaN_3_ (3.0 equiv), 1.0 mmol Bi(NO_3_)_3_·5H_2_O, 5.0 mL of CH_3_CN, 2–5 min, 125 °C, 150 W. Products **6d** and **11** were recrystallized by ethanol.

To support the proposed reaction pathway of Scheme 5, some control reactions were undertaken and are shown in Scheme 6. It is known that thiourea derived from secondary amine and cyclic thiourea cannot form a carbodiimide due to geometrical restrictions but can be converted to bicyclic tetrazoles promoted by Hg salts via an addition–elimination pathway.^36^ Under the bismuth-promoted condition, compounds **7**–**8** did not react, with reagents being recovered, equations A and B of Scheme 6, while thiourea **9** afforded urea **6d**(37) in excellent yield, equation C. These results suggest that a carbodiimide like **III/VI** should be involved in the bismuth-promoted reactions of thiourea to tetrazole. Besides, *N*-benzoylthiourea **10** was transformed into tetrazole **11**(38) with the Bz group at endocyclic nitrogen, equation D, whose regioselectivity agrees with the proposed reaction pathway via more stable carbodiimide **III**, due to its N-acyliminium cation character. In a test reaction, *N*,*N*′-dicyclohexylcarbodiimide **12** was submitted to the standard reaction condition without bismuth salt, and only urea **13** was isolated, albeit in low yield, equation E of Scheme 6, which indicates the essential role of protonated carbodiimide in the reaction course.

In addition to the proposed mechanistic rationalization to explain such regioselectivity, under the bismuth-promoted reaction heating condition, the Dimroth rearrangement of **3′** to **3** may also be responsible for the isolated regioisomer.^39^ Particularly, the increased proportion of isomer **3m** (thermodynamic isomer^39^) in comparison to isomer **3m′** (kinetic isomer^39^) under more prolonged reaction time (**3m/3m′** from 3:93 to 60:40, 15 to 40 min, from condition A, Scheme 4 to condition A, Scheme 2) reinforces that the Dimroth rearrangement should be an important factor in the observed regioselectivity.^39^

## Conclusions

The nontoxic Bi(NO_3_)_3_·5H_2_O is adequate as a thiophilic Lewis acid to promote the three-component syntheses of 5-aminotetrazoles and bistetrazoles. The method applies to aliphatic and aromatic amines containing electron-rich and -deficient functional groups. It affords the desired products in good yields and short reaction time under microwave heating, with simple purification, which dispenses column chromatography. The method exhibits a high regioselectivity, which is strongly determined by the electronic density of the amine employed. This regioselectivity is opposite that obtained by several thiourea desulfurization methods promoted by thiophilic metals and metal-free protocols. In addition, this study shows that a simple NMR investigation based on the splitting pattern of the aromatic groups attached at positions 1 and 5 of the tetrazole ring unequivocally indicates which regioisomer is obtained in each synthetic route.

## Experimental Section

### General Experimental Information

Commercially available chemicals and solvents were used without further purification, unless otherwise noted. Melting points were determined on a Microqumica MQAPF 301 hot plate apparatus and were uncorrected. Infrared spectra were recorded as KBr discs on a Shimadzu IR Affinity-1 instrument. Thin layer chromatography (TLC) was performed using TLC silica gel 60 F_254_ glass plates. ^1^H NMR and ^13^C NMR spectra were recorded on a Bruker Avance III NMR spectrometer (500 MHz for ^1^H and 125 MHz for ^13^C) with tetramethylsilane as an internal standard. Chemical shifts (δ) are reported in parts per million relative to the residual solvent signals, and coupling constants (J) are reported in hertz. The multiplicities are described as brs = broad signal, s = singlet, d = doublet, t = triplet, q = quartet, dd = doublet of doublets, dt = doublet of triplets, and m = multiplet. High-resolution mass spectra (HRMS) were recorded using electrospray ionization (ESI) (hybrid linear ion trap–orbitrap FT-MS/MS and QqTOF Microtof-QII models). Reagents and materials were of the highest commercially available grade and used without further purification. Microwave heating reactions were performed in a CEM Discover SP using the 10 or 30 mL Pyrex pressure vial for closed vessel reactions, under the indicated power automatically to reach and maintain the set temperature, specified in each case, with infrared (IR) temperature control and medium stirring speed using cylindrical stir bars (10 × 3 mm), with a default ramp time of 2 min. Thioureas and bis thioureas were prepared according to known procedures.^43^

***CAUTION****! Metal azides and tetrazoles are potentially explosive. However, all tetrazoles synthesized here were stored at room temperature (25–32 °C) and remained intact in terms of their chemical composition, even though they were prepared more than 5 years ago.*

### General Procedure: Synthesis of 1-Substituted 5-aminotetrazoles (**3a–3o**)

#### General Procedure for Bicomponent Condition – Methods A and B

To a 10 mL microwave reactor vial, the appropriate thiourea (1.0 mmol, 1.0 equiv) and sodium azide (3.0 mmol, 3.0 equiv) were added. The mixture was dissolved in 5.0 mL of CH_3_CN (for method A) or DMF (for method B), followed by the addition of Bi(NO_3_)_3_·5H_2_O (1.0 mmol, 1.0 equiv) and triethylamine (3.0 mmol, 3.0 equiv). The vial was capped, and the solution was stirred for 1 min to ensure complete homogeneity. The reaction mixture was subsequently taken into a microwave reactor and heated at 125 °C with 150 W of power within the time indicated in each case. Afterward, the mixture was cooled down and filtered through a pad of Celite to retain a dark precipitate, which was washed with 10 mL of acetonitrile. The combined organic phase was concentrated under vacuum, and the residue obtained was purified, providing the respective product **3.**

#### General Procedure for the Three-Component Condition—Method C

To a 10 mL microwave reactor vial were added the appropriate amine (1.0 mmol, 1.0 equiv), phenyl isothiocyanate (1.0 mmol, 1.0 equiv), and sodium azide (3.0 mmol, 3.0 equiv). The mixture was dissolved in 5.0 mL of CH_3_CN, followed by the addition of Bi(NO_3_)_3_·5H_2_O (1.0 mmol, 1.0 equiv) and triethylamine (3.0 mmol, 3.0 equiv). The vial was capped, and the solution was stirred for 1 min to ensure complete homogeneity. The reaction mixture was subsequently taken into a microwave reactor and heated at 125 °C with 150 W of power within the time indicated in each case. Afterward, the mixture was cooled down and filtered through a pad of Celite to retain a dark precipitate, which was washed with 10 mL of acetonitrile. The combined organic phase was concentrated under vacuum, and the residue obtained was purified, providing the respective product **3.**

##### 1-Benzyl-*N*-phenyl-1*H*-tetrazol-5-amine (**3a**)^39^

*02 min* (205 mg, 89% yield), white solid, purified by recrystallization in ethanol, mp: 193–195 °C. ^1^H NMR (DMSO-*d*_6_, 500 MHz) δ 9.50 (s, 1H), 7.65 (d, J = 8.0 Hz, 2H), 7.40–7.30 (m, 5H), 7.25 (d, *J* = 7.5 Hz, 2H), 7.01 (t, *J* = 7.5 Hz, 1H), 5.67 (s, 2H). ^13^C{^1^H} NMR (DMSO-*d*_6_, 125 MHz) δ: 152.9, 140.2, 135.0, 129.4, 129.2, 128.5, 127.8, 122.3, 118.0, 48.6. IR (KBr): ν/cm^–1^ 3277, 3130, 1616, 1575, 1541, 1498, 1332, 715, 688. HRMS (ESI): *m*/*z* calc. for C_14_H_14_N_5_ [M + H]^+^ 252.1249, found 252.1243.

##### 1-Butyl-*N*-phenyl-1*H*-tetrazol-5-amine (**3b**)^40^

*3 min.* (79 mg, 37% yield), white solid, purified by recrystallization in ethanol, mp: 130–132 °C. ^1^H NMR (DMSO-*d*_6_, 500 MHz) δ 9.22 (s, 1H), 7.64 (d, *J* = 8.0 Hz, 2H), 7.35 (t, *J* = 8.0 Hz, 2H), 7.00 (t, *J* = 7.5 Hz, 1H), 4.33 (t, *J* = 7.0 Hz, 2H), 1.78 (quin, *J* = 7.0 Hz, 2H), 1.31 (sex, *J* = 7.0 Hz, 2H), 0.91 (t, *J* = 7.0 Hz, 3H). ^13^C{^1^H} NMR (DMSO-*d*_6_, 125 MHz) δ 152.7, 140.5, 129.4, 122.3, 118.0, 45.4, 31.0, 19.5, 13.8. IR (KBr): ν/cm^–1^ 3273, 3124, 3057, 2960, 1618, 1575, 630. HRMS (ESI) *m*/*z* calcd for C_11_H_16_N_5_ [M + H]^+^ 218.1406, found 218.1401.

##### 1-Ethyl-*N*-phenyl-1*H*-tetrazol-5-amine (**3c**)^40^

*2 min.* (60 mg, 32% yield), white solid, purified by recrystallization in ethanol, mp: 160–162 °C. ^1^H NMR (DMSO-*d*_6_, 500 MHz) δ: 9.22 (s, 1H), 7.65 (d, *J* = 8.0 Hz, 2H), 7.33 (t, *J* = 8.0 Hz, 2H), 6.98 (t, *J* = 7.5 Hz, 1H), 4.35 (q, *J* = 7.5 Hz, 2H), 1.40 (t, *J* = 7.5 Hz, 3H). ^13^C{^1^H} NMR (DMSO-*d*_6_, 125 MHz) δ 152.4, 140.5, 129.3, 122.1, 118.0, 40.9, 14.6. IR (KBr): ν/cm^–1^ 3313, 3132, 1612, 1573, 1535, 752, 559. HRMS (ESI) *m*/*z* calcd for C_9_H_12_N_5_ [M + H]^+^ 190.1093, found 190.1087.

##### *N*^1^,*N*^1^-Dimethyl-*N*^5^-phenyl-1*H*-tetrazole-1,5-diamine (**3d**)

20 min. (134 mg, 66% yield), yellow oil, purified by flash column chromatography (10–30% EtOAc in hexanes). ^1^H NMR (DMSO-*d*_6_, 500 MHz) δ 9.26 (s, 1H), 7.78–7.80 (m, 2H), 7.35–7.31 (m, 2H), 6.98 (t, *J* = 7.5 Hz, 1H). ^13^C{^1^H} NMR (DMSO-*d*_6_, 125 MHz) δ 149.8, 139.9, 129.2, 122.2, 118.1, 47.1. IR (KBr): ν/cm^–1^ 3383, 3014, 1730, 1618, 1604,1583, 1498, 1217, 754.

##### 1-(*tert*-Butyl)-*N*-phenyl-1*H*-tetrazol-5-amine (**3e**)^40^

*5 min.* (68 mg, 32% yield), yellow solid, purified by recrystallization in ethanol, mp: 177–179 °C. ^1^H NMR (DMSO-*d*_6_, 500 MHz) δ 8.26 (s, 1H), 7.41 (dd, *J* = 1.0 Hz, *J* = 7.5 Hz, 2H), 7.29 (tl, *J* = 7.5 Hz, 2H), 6.98 (tl, *J* = 7.5 Hz, 1H), 1.75 (s, 9H). ^13^C{^1^H} NMR (DMSO-*d*_6_, 125 MHz) δ 152.5, 141.6, 129.2, 122.2, 118.9, 60.0, 28.7. IR (KBr): ν/cm^–1^ 3313, 2995, 1600, 1562, 749, 551. HRMS (ESI) *m*/*z* calcd for C_11_H_16_N_5_ [M + H]^+^ 218.1406, found 218.1403.

##### 1-Cyclohexyl-*N*-phenyl-1*H*-tetrazol-5-amine (**3f**)^41^

*3 min.* (121 mg, 50% yield), white solid, purified by recrystallization in ethanol, mp: 220–221 °C. ^1^H NMR (DMSO-*d*_6_, 500 MHz) δ 9.16 (s, 1H), 7.64 (d, *J* = 7.5 Hz, 2H), 7.35 (t, *J* = 7.5 Hz, 2H), 7.00 (t, *J* = 7.5 Hz, 1H), 4.49 (m, 1H), 2.03–1.70 (m, 7H), 1.44 (q, *J* = 13.0 Hz, 2H), 1.28 (t, *J* = 13.0 Hz, 1H). ^13^C{^1^H} NMR (DMSO-*d*_6_, 125 MHz) δ 151.9, 140.5, 129.4, 122.2, 118.1, 55.1, 32.5, 25.3, 25.1. IR (KBr): ν/cm^–1^ 3278, 3120, 3043, 2933, 1610, 1575, 1531, 684. HRMS (ESI) *m*/*z* calcd for C_13_H_18_N_5_ [M + H]^+^ 244.1562, found 244.1555.

##### *N*,1-Diphenyl-1*H*-tetrazol-5-amine (**3g**)^42^

*2 min.* (187 mg, 69% yield), white solid, purified by recrystallization in ethanol, mp: 163–164 °C. ^1^H NMR (DMSO-*d*_6_, 500 MHz) δ 9.31 (s, 1H), 7.67–7.61 (m, 4H), 7.32 (t, *J* = 7.5 Hz, 4H), 7.00 (t, *J* = 7.5 Hz, 2H). ^13^C{^1^H} NMR (DMSO-*d*_6_, 125 MHz) δ 152.8, 140.3, 133.5, 130.5, 130.4, 129.2, 126.0, 122.6, 118.7. IR (KBr): ν/cm^–1^ 3118, 3072, 2995, 1610, 1570, 1529, 690.

##### 1-(2-Methoxyphenyl)-*N*-phenyl-1*H*-tetrazol-5-amine (**3h**)^25^

*2 min.* (218 mg, 83% yield), white solid, purified by recrystallization in ethanol, mp: 183–185 °C. ^1^H NMR (DMSO-*d*_6_, 500 MHz) δ 9.08 (s, 1H), 7.66–7.61 (m, 3H), 7.53 (dd, *J* = 1.5 Hz, *J* = 7.5 Hz, 1H), 7.33–7.28 (m, 3H), 7.16 (dt, *J* = 7.5 Hz, *J* = 1.5 Hz, 1H), 6.98 (tl, *J* = 7.5 Hz, 1H), 3.78 (s, 3H). ^13^C{^1^H} NMR (DMSO-*d*_6_, 125 MHz) δ 155.0, 153.4, 140.2, 132.7, 129.3, 129.2, 122.4, 121.4, 121.3, 118.5, 113.6, 56.4. IR (KBr): ν/cm^–1^ 3251, 3199, 1612, 1573, 1533, 756, 698. HRMS (ESI) *m*/*z* calcd for C_14_H_14_N_5_O [M + H]^+^ 268.1198, found 268.1193.

##### *N*-Phenyl-1-(*O*-tolyl)-1*H*-tetrazol-5-amine (**3i**)^19e^

*2 min.* (193 mg, 77% yield), white solid, purified by recrystallization in ethanol, mp: 189–193 °C. ^1^H NMR (DMSO-*d*_6_, 500 MHz) δ 9.27 (s, 1H), 7.65 (d, *J* = 8.0 Hz, 2H), 7.52 (m, 4H), 7.31 (t, *J* = 8.0 Hz, 2H), 7.00 (t, *J* = 7.5 Hz, 1H), 2.05 (s, 3H). ^13^C{^1^H} NMR (DMSO-*d*_6_, 125 MHz) δ 152.4, 139.4, 135.3, 131.4, 131.1, 130.6, 128.5, 127.7, 127.1, 121.7, 117.8, 16.6. IR (KBr): ν/cm^–1^ 3244, 3088, 1618, 1602, 1571, 752, 694. HRMS (ESI) *m*/*z* calcd for C_14_H_14_N_5_ [M + H]^+^ 252.1249, found 252.1243.

##### *N*-(4-Nitrophenyl)-1-phenyl-1*H*-tetrazol-5-amine (**3j′**)^42^

*5 min.* (115 mg, 78% yield), orange solid, purified by recrystallization in ethanol. mp: 240–242 °C. ^1^H NMR (DMSO-*d*_6_, 500 MHz) δ 10.15 (s, 1H), 8.23 (d, *J* = 9.0 Hz, 2H), 7.85 (d, *J* = 9.0 Hz, 2H), 7.66–7.61 (m, 5H). ^13^C{^1^H} NMR (DMSO-*d*_6_, 125 MHz) δ 151.1, 145.8, 140.8, 132.4, 130.0, 129.6, 125.3, 124.8, 117.1. IR (KBr): ν/cm^–1^ 3385, 1622, 1585, 1535, 1334, 850, 690.

##### *N*-(4-Chlorophenyl)-1-phenyl-1*H*-tetrazol-5-amine (**3k′**)^42^

*5 min.* (192 mg, 67% yield), yellow solid, purified by recrystallization in ethanol. mp: 190–192 °C. ^1^H NMR (DMSO-*d*_6_, 500 MHz) δ 9.46 (s, 1H), 7.83–7.61 (m, 7H), 7.38 (t, J = 10.0 Hz, 2H). ^13^C{^1^H} NMR (DMSO-*d*_6_, 125 MHz) δ 152.6, 139.3, 133.4, 130.5, 130.3, 129.0, 126.2, 126.0, 120.3. IR (KBr): ν/cm^–1^ 3259, 3194, 1612, 1570, 763, 686. HRMS (ESI) *m*/*z* calcd for C_13_H_11_ClN_5_ [M + H]^+^ 272.0703, found 272.0697.

##### *N*-(4-Methoxy-2-nitrophenyl)-1-phenyl-1*H*-tetrazol-5-amine (**3l′**)

*3 min.* (204 mg, 73% yield), orange solid, purified by recrystallization in ethanol. mp: 185–187 °C. ^1^H NMR (DMSO-*d*_6_, 500 MHz) δ 9.80 (s, 1H), 8.14 (d, *J* = 9.0 Hz, 1H), 7.76–7.64 (m, 5H), 7.61 (d, *J* = 3.0 Hz, 1H), 7.46 (dd, *J* = 3.0 Hz, *J* = 9.0 Hz, 1H), 3.81 (s, 3H). ^13^C{^1^H} NMR (DMSO-*d*_6_, 125 MHz) δ 155.0, 152.3, 138.9, 133.0, 130.8, 130.7, 128.8, 125.2, 124.2, 123.4, 109.6, 56.4. IR (KBr): ν/cm^–1^ 2958, 2927, 1732, 1635, 1604, 1531, 1284, 752, 605. HRMS (ESI) *m*/*z* calcd for C_14_H_12_N_6_O_3_ [M + H]^+^ 313.1004, found 313.1044.

##### Mixture of 1-(4-Methoxyphenyl)-*N*-phenyl-1*H*-tetrazol-5-amine (**3m**) and *N*-(4-Methoxyphenyl)-1-phenyl-1*H*-tetrazol-5-amine (**3m′**)^19e^

*3 min.* (70 mg, 59% yield, 0.5 mmol scale), white solid, purified by recrystallization in ethanol. ^1^H NMR (DMSO-*d*_6_, 500 MHz) δ 9.14 (s, 1H), 8.32 (s, 1H), 7.63–7.61 (m, 2H), 7.57–7.54 (m, 2H), 7.35–7.24 (m, 5H), 7.19–7.16 (m, 2H), 6.99 (t, *J* = 7.5 Hz, 1H), 6.88–6.83 (m, 2H), 3.86 (s, 3H), 3.71 (s, 3H). ^13^C{^1^H} NMR (DMSO-*d*_6_, 125 MHz) δ 160.0, 154.0, 152.6, 152.2, 139.5, 132.6, 128.4, 127.1, 125.2, 121.6, 121.2, 119.7, 119.6, 117.8, 117.8, 114.7, 113.7, 113.6, 55.3, 54.8. IR (KBr): ν/cm^–1^ 3259, 3194, 1612, 1570, 1527, 686.

##### Mixture of *N*-Phenyl-1-(*p*-tolyl)-1*H*-tetrazol-5-amine (**3n**) and 1-Phenyl-*N*-(*p*-tolyl)-1*H*-tetrazol-5-amine (**3n′**)^25^

*3 min*. (147 mg, 66% yield), yellow solid, purified by recrystallization in ethanol. ^1^H NMR (DMSO-*d*_6_, 500 MHz) δ 9.27 (s, 1H), 9.23 (s, 1H), 7.66–7.61 (m, 6H), 7.54–7.49 (m, 5H), 7.46–7.44 (m, 2H), 7.32–7.29 (m, 2H), 7.13–7.10 (m, 2H), 6.99 (t, *J* = 7.5 Hz, 1H), 2.43 (s, 3H), 2.25 (s, 3H). ^13^C{^1^H} NMR (DMSO-*d*_6_, 125 MHz) δ 152.0, 151.9, 139.5, 139.5, 132.7, 130.6, 130.1, 130.0, 129.6, 129.6, 128.8, 128.4, 125.2, 125.2, 121.7, 118.1, 118.0, 117.8, 20.4, 20.0. IR (KBr): ν/cm^–1^ 3232, 3184, 1608, 1580, 1527, 1496, 1230, 765, 694.

##### Mixture of *N*-Phenyl-1*H*-tetrazol-5-amine (**3o**) and 1-Phenyl-1*H*-tetrazol-5-amine (**3o′**)^19a^

*30 min*. (114 mg, 67% yield), white solid, purified by recrystallization in ethanol. ^1^H NMR (DMSO-*d*_6_, 500 MHz) δ 9.04 (s, 1H), 7.82 (d, *J* = 7.5 Hz, 2H), 7.57–7.44 (m, 6H), 7.24 (t, *J* = 7.5 Hz, 2H), 7.02 (t, *J* = 7.5 Hz, 1H). ^13^C{^1^H} NMR (DMSO-*d*_6_, 125 MHz) δ 156.7, 154.7, 138.3, 134.1, 128.7, 128.2, 128.0, 123.1, 122.7, 120.8. IR (KBr): ν/cm^–1^ 3427, 3319, 1624, 1583, 1562, 1488, 1107, 744, 662.

### Synthesis of Bis-5-aminotetrazoles (**5a**–**5c**)

#### General Procedure for Bicomponent and Bidirectional Condition

To a 10 mL microwave reactor vial were added the appropriate bisthiourea (1.0 mmol, 1.0 equiv) and sodium azide (6.0 mmol, 6.0 equiv). The mixture was dissolved in 5 mL of DMF, followed by the addition of Bi(NO_3_)_3_.5H_2_O (2.0 mmol, 2.0 equiv) and triethylamine (6.0 mmol, 6.0 equiv). The vial was capped, and the resulting solution was stirred for 2 min to ensure complete homogeneity. The reaction mixture was subsequently taken into a microwave reactor and heated at 125 °C with 150 W of power within the time indicated in each case. Afterward, the mixture was cooled down and filtered through a pad of Celite to retain a dark precipitate, which was washed with 10 mL of acetonitrile. The joined organic phase was concentrated, and the residue obtained was purified, providing the respective product 5.

#### General Procedure for the Multicomponent Condition

To a 10 mL microwave reactor vial, were added the appropriate diamine (1.0 mmol, 1.0 equiv), phenyl isothiocyanate (2.0 mmol, 2.0 equiv), and sodium azide (6.0 mmol, 6.0 equiv). The mixture was dissolved in 5 mL of CH_3_CN, followed by the addition of Bi(NO_3_)_3_·5H_2_O (2.0 mmol, 2.0 equiv) and triethylamine (6.0 mmol, 6.0 equiv). The vial was capped, and the resulting solution was stirred for 2 min to ensure complete homogeneity. The reaction mixture was subsequently taken into a microwave reactor and heated at 125 °C with 150 W of power within the time indicated in each case. Afterward, the mixture was cooled down and filtered through a pad of Celite to retain a dark precipitate, which was washed with 10 mL of acetonitrile. The combined organic phase was concentrated, and the residue obtained was purified, providing the respective product **5**.

##### 1,1′-(Propane-1,3-diyl)bis(*N*-phenyl-1*H*-tetrazol-5-amine) (**5a**)

*5 min.* (141 mg, 39% yield), white solid, purified by recrystallization in ethanol. mp: 237–238 °C. ^1^H NMR (DMSO-*d*_6_, 500 MHz) δ 9.22 (s, 2H), 7.63 (dd, J = 9.0 Hz, *J* = 1.0 Hz, 4H), 7.34 (dd, *J* = 9.0 Hz, *J* = 7.5 Hz, 4H), 7.00 (tt, *J* = 7.5 Hz, *J* = 1.2 Hz, 2H), 4.43 (t, *J* = 5.0 Hz, 4H), 2.44 (quint, *J* = 5.0 Hz, 2H). ^13^C{^1^H} NMR (DMSO-*d*_6_, 125 MHz) δ 152.7, 140.3, 129.3, 122.2, 118.1, 43.1, 27.9. IR (KBr): ν/cm^–1^ 3321, 3132, 1620, 1581, 1531, 1118, 690. HRMS (ESI) *m*/*z* calcd for C_17_H_19_N_10_ [M + H]^+^ 363.1794, found 363.1789.

##### 1,1′-(Butane-1,4-diyl)bis(*N*-phenyl-1*H*-tetrazol-5-amine) (**5b**)

*3 min.* (199 mg, 53% yield, white solid, purified by recrystallization in ethanol. mp: 269–270 °C. ^1^H NMR (DMSO-*d*_6_, 500 MHz) δ: 9.22 (s, 2H), 7.63 (d, *J* = 5.0 Hz, 4H), 7.34 (t, *J* = 5.0 Hz, 4H), 7.00 (t, *J* = 5.0 Hz, 2H), 4.38 (sl, 4H), 1.84 (sl, 4H). ^13^C{^1^H} NMR (DMSO-*d*_6_, 125 MHz) δ 152.6, 140.3, 129.3, 122.2, 118.0, 45.0, 26.0. IR (KBr): ν/cm^–1^ 3325, 3124, 3055, 2966, 1616, 1577, 1523, 740. HRMS (ESI) *m*/*z* calcd for C_18_H_21_N_10_ [M + H]^+^ 377.1951, found 377.1947.

##### 1,1′-(Hexane-1,6-diyl)bis(*N*-phenyl-1*H*-tetrazol-5-amine) (**5c**)

*3 min.* (246 mg, 61% yield), white solid, purified by recrystallization in ethanol. mp: 233–235 °C. ^1^H NMR (DMSO-*d*_6_, 500 MHz) δ 9.20 (s, 2H), 7.63 (dd, *J* = 9.0 Hz, *J* = 1.0 Hz, 4H), 7.33 (dd, *J* = 8.5, *J* = 7.5 Hz, 4H), 6.99 (tt, 4H, *J* = 7.5 Hz), 4.31 (t, *J* = 5.0 Hz, 4H), 1.79 (quint, *J* = 5.0 Hz, 4H), 1.34 (m, 4H). ^13^C{^1^H} NMR (DMSO-*d*_6_, 125 MHz) δ 152.6, 140.4, 129.3, 122.2, 118.0, 45.5, 28.7, 25.7. IR (KBr): ν/cm^–1^ 3309, 3043, 2931, 1620, 1577, 1523, 744, 686. HRMS (ESI) *m*/*z* calcd for C_20_H_25_N_10_ [M + H]^+^ 405.2264, found 405.2257.

#### Synthesis of **11**

To a 10 mL microwave reactor vial, *N*-(phenylcarbamothioyl)benzamide **10** (1.0 mmol, 1.0 equiv) and sodium azide (3.0 mmol, 3.0 equiv) were added. The mixture was dissolved in 5.0 mL of DMF, followed by the addition of Bi(NO_3_)_3_·5H_2_O (1.0 mmol, 1.0 equiv) and triethylamine (3.0 mmol, 3.0 equiv). The vial was capped, and the solution was stirred for 1 min to ensure complete homogeneity. The reaction mixture was subsequently taken into a microwave reactor and heated at 125 °C with 150 W of power over 10 min. Afterward, the mixture was cooled down and filtered through a pad of Celite to retain a dark precipitate, which was washed with 10 mL of acetonitrile. The combined organic phase was concentrated under vacuum, and the residue obtained was purified, providing the respective product 11.^38^ (124 mg, 47% yield), white solid, purified by recrystallization in ethanol. mp: 165–168 °C °C. ^1^H NMR (DMSO-*d*_6_, 500 MHz) δ 10.24 (s, 1H), 7.95 (d, *J* = 5.0 Hz, 2H), 7.78 (d, *J* = 5.0 Hz, 2H), 7.59 (t, *J* = 5.0 Hz, 1H), 7.53 (t, *J* = 5.0 Hz, 2H), 7.35 (t, *J* = 5.0 Hz, 2H), 7.10 (t, *J* = 5.0 Hz, 1H). ^13^C{^1^H} NMR (DMSO-*d*_6_, 125 MHz) δ: 166.0, 139.6, 135.4, 131.9, 129.0, 128.8, 128.0, 124.1, 120.8. IR (KBr): ν/cm^–1^ 3344, 3051, 1654, 1600, 1531, 1438, 690, 648.

### Reaction Scope for Substituted Isothiocyanates

#### General Procedure for Bicomponent Condition—Method A

To a 10 mL microwave reactor vial, the appropriate thiourea (0.5 mmol, 1.0 equiv) and sodium azide (1.5 mmol, 3.0 equiv) were added. The mixture was dissolved in 2.5 mL of CH_3_CN, followed by the addition of Bi(NO_3_)_3_·5H_2_O (0.5 mmol, 1.0 equiv) and triethylamine (1.5 mmol, 3.0 equiv). The vial was capped, and the solution was stirred for 1 min to ensure complete homogeneity. The reaction mixture was subsequently taken into a microwave reactor and heated at 125 °C with 150 W of power within the time indicated in each case. Afterward, the mixture was cooled and filtered through a pad of Celite to retain a dark precipitate, which was washed with 15 mL of acetonitrile. The combined organic phase was concentrated under vacuum, and the residue obtained was purified, providing the respective product **3.**

##### *N*-(4-Methoxyphenyl)-1-phenyl-1*H*-tetrazol-5-amine (**3m′**)^19e^

*15 min* (35 mg, 27% yield), pale yellow solid, purified by dissolving in ethanol and clouded with water. ^1^H NMR (DMSO-*d*_6_, 500 MHz) δ 9.17 (s, 1H), 7.63 (d, *J* = 7.8 Hz, 2H), 7.57 (d, *J* = 8.9 Hz, 2H), 7.31 (t, *J* = 7.8 Hz, 2H), 7.18 (d, *J* = 8.9 Hz, 2H), 6.99 (t, *J* = 7.3 Hz, 1H), 3.86 (s, 3H). ^13^C{^1^H} NMR (DMSO-*d*_6_, 125 MHz) δ 160.4, 152.5, 139.9, 128.7, 127.5, 125.6, 121.9, 118.1, 115.0, 55.6. IR (KBr): ν/cm^–1^ 3252, 3202, 1616, 1535, 1516, 1501, 1250, 873, 748.

##### Mixture of *N*-Phenyl-1-(*p*-tolyl)-1*H*-tetrazol-5-amine (**3n**) and 1-Phenyl-*N*-(*p*-tolyl)-1*H*-tetrazol-5-amine (**3n′**)^25^

*15 min*. (44.7 mg, 36% yield, quantified by NMR), pale yellow solid, purified by dissolving in ethanol, and clouded with water. ^1^H NMR (DMSO-*d*_6_, 500 MHz) δ 9.22 (s, 1H), 9.18 (s, 1H), 7.66–7.62 (m, 6H), 7.54–7.49 (m, 5H), 7.46–7.45 (m, 2H), 7.33–7.30 (m, 2H), 7.12 (d, *J* = 8.2 Hz, 2H), 7.00 (t, *J* = 7.5 Hz, 1H), 2.43 (s, 3H), 2.26 (s, 3H). ^13^C{^1^H} NMR (DMSO-*d*_6_, 125 MHz) δ: 152.0, 151.9, 139.5, 139.5, 132.7, 130.6, 130.1, 130.0, 129.6, 129.6, 128.8, 128.4, 125.2, 125.2, 121.7, 118.1, 118.0, 117.8, 20.4, 20.0.

##### Residual 1-(4-Methylphenyl)-3-phenylurea (**6a**)^44,45^

(8.3 mg, 7% yield, quantified by NMR). ^1^H NMR (DMSO-*d*_6_, 500 MHz) δ: 8.58 (s, 1H), 8.52 (s, 1H), 7.43 (m, 2H), 7.34–7.32 (m, 2H), 7.27 (t, *J* = 8.0 Hz, 2H), 7.07 (d, *J* = 8.0 Hz, 2H), 6.95 (t, *J* = 7.5 Hz, 1H), 2.24 (s, 3H). ^13^C{^1^H} NMR (DMSO-*d*_6_, 125 MHz) δ: 152.5, 139.8, 137.1, 130.6, 129.1, 121.7, 118.3, 118.1, 20.3.

##### *N*,1-Bis(4-chlorophenyl)-1*H*-tetrazol-5-amine (**3p**)^19e^

*15 min* (109 mg, 73% yield), white solid, purified by dissolving in ethanol and clouded with water, mp: 199–201 °C. ^1^H NMR (DMSO-*d*_6_, 500 MHz) δ 9.47 (s, 1H), 7.73 (s, 4H), 7.66 (d, *J* = 8.9 Hz, 2H), 7.38 (d, *J* = 8.9 Hz, 2H). ^13^C{^1^H} NMR (DMSO-*d*_6_, 125 MHz) δ: 152.2, 138.7, 134.8, 131.7, 129.9, 128.6, 127.8, 125.8, 119.8. IR (KBr): ν/cm^–1^ 3267, 3094, 1609, 1566, 1489, 1408, 1234, 1087, 802.

##### *N*-(4-Chlorophenyl)-1-(*O*-tolyl)-1*H*-tetrazol-5-amine (**3q**)^46^

*15 min* (105 mg, 73% yield), white solid, purified by dissolving in ethanol and clouded with water, mp: 168–175 °C. ^1^H NMR (CDCl_3_, 500 MHz) δ 7.55 (t, *J* = 7.6 Hz, 1H), 7.52 (d, *J* = 8.8 Hz, 2H), 7.49 (d, *J* = 7.6 Hz, 1H), 7.45 (t, *J* = 7.6 Hz, 1H), 7.34 (d, *J* = 7.6 Hz, 1H), 7.31 (d, *J* = 8.8 Hz, 2H), 6.12 (s, 1H), 2.17 (s, 3H). ^13^C{^1^H} NMR (CDCl_3_, 125 MHz) δ: 152.1, 136.8, 136.6, 132.5, 131.8, 131.0, 129.5, 128.6, 128.1, 127.4, 119.4, 17.6. IR (KBr): ν/cm^–1^ 3244, 3186, 1616, 1566, 1531, 1493, 1384, 1092, 767.

##### Mixture of *N*-(4-Chlorophenyl)-1-(4-methoxyphenyl)-1*H*-tetrazol-5-amine (**3r**) and 1-(4-Chlorophenyl)-*N*-(4-methoxyphenyl)-1*H*-tetrazol-5-amine (**3r′**)^22c^

*15 min*. (86.0 mg, 57% yield, quantified by NMR), yellow solid, purified by dissolving in ethanol and clouded with water, mp: 161–165 °C. ^1^H NMR of **3r** (DMSO-*d*_6_, 500 MHz) δ 9.34 (s, 1H), 7.68 (d, *J* = 8.8 Hz, 2H), 7.57 (d, *J* = 8.8 Hz, 2H), 7.37 (d, *J* = 8.8 Hz, 2H), 7.18 (d, *J* = 8.8 Hz, 2H), 3.86 (s, 3H). ^13^C{^1^H} NMR of 3r (DMSO-*d*_6_, 125 MHz): δ 160.4, 152.3, 138.9, 128.6, 127.6, 125.6, 125.4, 119.7, 115.0, 55.6.

^1^H NMR of **3r′** (DMSO-*d*_6_, 500 MHz) δ 7.73–7.71 (m, 4H), 7.51 (d, *J* = 8.9 Hz, 2H), 6.91 (d, *J* = 8.9 Hz, 2H), 3.73 (s, 3H). ^13^C{^1^H} NMR of **3r′** (DMSO-*d*_6_, 125 MHz) δ 154.8, 129.9, 127.8, 120.3, 114.0, 55.2.

##### Residual 1-(4-Chlorophenyl)-3-(4-methoxyphenyl)urea (**6b**)^44,47^

15.9 mg, 11% yield, quantified by NMR. ^1^H NMR (DMSO-*d*_6_, 500 MHz) δ: 8.70 (s, 1H), 8.48 (s, 1H), 7.47 (d, *J* = 8.7 Hz, 2H), 7.34–7.33 (m, 2H), 7.30 (d, *J* = 8.7 Hz, 2H), 6.87 (d, *J* = 8.8 Hz, 2H), 3.71 (s, 3H). ^13^C{^1^H} NMR (DMSO-*d*_6_, 125 MHz) δ: 154.6, 152.6, 138.9, 132.5, 128.6, 125.1, 120.2, 119.6, 55.2.

##### *N*,1-Bis(*p*-tolyl)-1*H*-tetrazol-5-amine (**3s**)^19b^

*15 min* (86 mg, 65% yield), white solid, purified by dissolving in ethanol and clouded with water, mp: 206–210 °C. ^1^H NMR (DMSO-*d*_6_, 500 MHz) δ 9.10 (s, 1H), 7.50–7.53 (m, 4H), 7.45 (d, *J* = 8.1 Hz, 2H), 7.12 (d, *J* = 8.2 Hz, 2H), 2.43 (s, 3H), 2.25 (s, 3H). ^13^C{^1^H} NMR (DMSO-*d*_6_, 125 MHz) δ: 152.4, 139.8, 137.3, 130.9, 130.5, 130.3, 129.1, 125.4, 118.4, 20.8, 20.3. IR (KBr): v/cm^–1^ 3229, 3179, 1605, 1562, 1508, 1385, 1231, 1080, 810.

##### *N*,1-Bis(4-methoxyphenyl)-1*H*-tetrazol-5-amine (**3t**)^19e^

*15 min* (61.4 mg, 41% yield, quantified by NMR), white solid, purified by dissolving in ethanol and clouded with water. ^1^H NMR (DMSO-*d*_6_, 500 MHz) δ: 8.95 (s, 1H), 7.55 (d, *J* = 8.9 Hz, 2H), 7.53 (d, *J* = 9.0 Hz, 2H), 7.17 (d, *J* = 8.9 Hz, 2H), 6.90 (d, *J* = 9.0 Hz, 2H), 3.86 (s, 3H), 3.72 (s, 3H). ^13^C{^1^H} NMR (DMSO-*d*_6_, 125 MHz) δ: 160.3, 154.6, 152.9, 133.0, 127.5, 125.6, 120.1, 115.0, 113.9, 55.6, 55.2.

##### Residual 1,3-Bis(4-methoxyphenyl)urea (**6c**)^48^

14.6 mg, 11% yield, quantified by NMR. ^1^H NMR (DMSO-*d*_6_, 500 MHz) δ: 8.34 (s, 2H), 7.33 (d, *J* = 8.9 Hz, 4H), 6.85 (d, *J* = 8.9 Hz, 4H), 3.71 (s, 6H). ^13^C{^1^H} NMR (DMSO-*d*_6_, 125 MHz) δ: 154.3, 152.9, 132.9, 119.9, 55.1.

#### General Procedure for the Three-Component Condition—Method C

To a 10 mL microwave reactor vial were added the appropriate amine (0.5 mmol, 1.0 equiv), isothiocyanate (0.5 mmol, 1.0 equiv), and sodium azide (1.5 mmol, 3.0 equiv). The mixture was dissolved in 2.5 mL of CH_3_CN, followed by the addition of Bi(NO_3_)_3_·5H_2_O (0.5 mmol, 1.0 equiv) and triethylamine (1.5 mmol, 3.0 equiv). The vial was capped, and the solution was stirred for 1 min to ensure complete homogeneity. The reaction mixture was subsequently taken into a microwave reactor and heated at 125 °C with 150 W of power within the time indicated in each case. Afterward, the mixture was cooled and filtered through a pad of Celite to retain a dark precipitate, which was washed with 20 mL of ethyl acetate. The combined organic phase was concentrated under vacuum, and the residue obtained was purified, providing the respective product **3.**

##### *N*,1-Bis(*p*-tolyl)-1*H*-tetrazol-5-amine (**3s**)^19b^

*15 min* (71 mg, 52% yield), white solid, purified by dissolving in ethanol and clouded with water, mp: 206–210 °C. IR (KBr): v/cm^–1^ 3229, 3179, 1605, 1562, 1508, 1385, 1231, 1080, 810.

##### *N*,1-Bis(4-methoxyphenyl)-1*H*-tetrazol-5-amine (**3t**)^19e^

*15 min* (38 mg, 25% yield), pale brown solid, purified by flash column chromatography (10–30% EtOAc in hexane) and recrystallized in ethanol. mp: 155–158 °C.

## Data Availability

The data underlying this study are available in the published article and its online Supporting Information.

## Supporting Information Available

Details of all experimental and spectral data: microwave reactor; summary of thioureas and bisthioureas used in this study; optimization studies; NMR, HRMS, and IR spectra (PDF)
