# Supplementary material for: Three-Component Synthesis of 1-Substituted 5-Aminotetrazoles Promoted by Bismuth Nitrate
Source: J Org Chem. 2024 Sep 13;89(19):14279–90. doi: 10.1021/acs.joc.4c01727 (PMC11459472; doi:10.1021/acs.joc.4c01727)
Supplement: Supplementary file 1 — jo4c01727_si_001.pdf [file jo4c01727_si_001.pdf]

SUPPLEMENTARY MATERIAL

Three-Component Synthesis of 1-Substituted 5-Aminotetrazoles Promoted by Bismuth Nitrate

Iva S. de Jesus[a], Amenson Trindade Gomes[a], Igor Sande[a] and Silvio Cunha[a,b*]

[a]*Instituto de Química, Universidade Federal da Bahia, Campus de Ondina, 40170-115, Salvador, BA, Brazil*

[b]*Instituto Nacional de Ciência e Tecnologia - INCT em Energia e Ambiente, Campus Ondina, Salvador, Bahia, 40170-290, Brazil*

* To whom correspondence should be addressed. E-mail: silviodc@ufba.br

Table of Contents

Microwave reactor......S2
Summary of thioureas used in this study......S2
Summary of bis thioureas used in this study S3
Optimization Studies......S3
NMR, FT-IR, and Spectra S4

Microwave reactor

Microwave heating reactions were performed in a CEM Discover SP using the 10 mL or 50 mL Pyrex pressure vial for closed vessel reactions, under the indicated power automatically to reach and maintain the set temperature, specified in each case, with infrared (IR) temperature control and medium stirring speed using cylindrical stir bars (10×3 mm), default ramp time of 2 minutes.

Summary of thioureas used in this study

Thioureas **1a-1t** were e prepared following the reported experimental procedure.

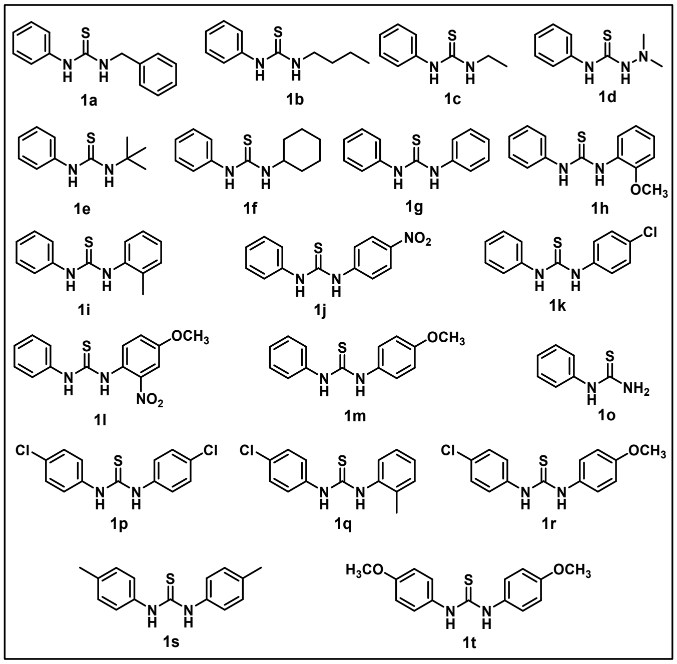

Scheme S1. Thioureas used as starting material during scope study

(1) Hassan, A. A.; Döpp, D. *J. Heterocyclic Chem.* Thermolysis of N, N''-Alkanediyl-bis[N'-organylthiourea] Derivatives**. 2006**, 43, 593-598.

Summary of bis thioureas used in this study

Bis thioureas **4a-4c** were e prepared following the reported experimental procedure.[1]

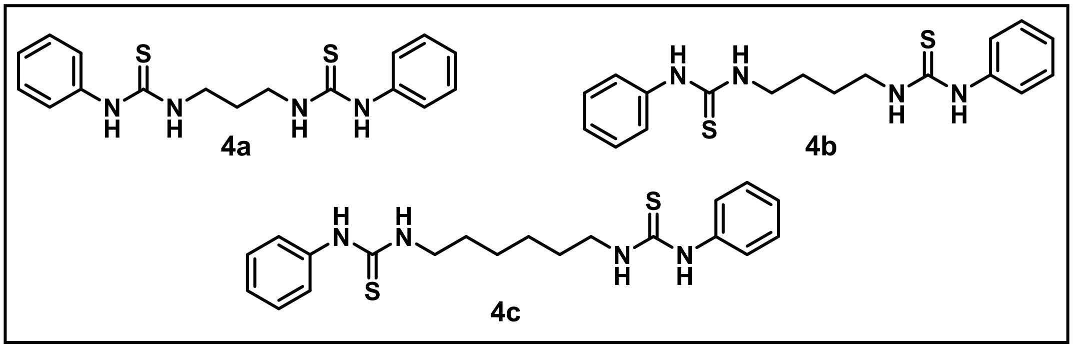

Scheme S2. Bis Thioureas

Optimization Studies

Table S1. Additional control studies.

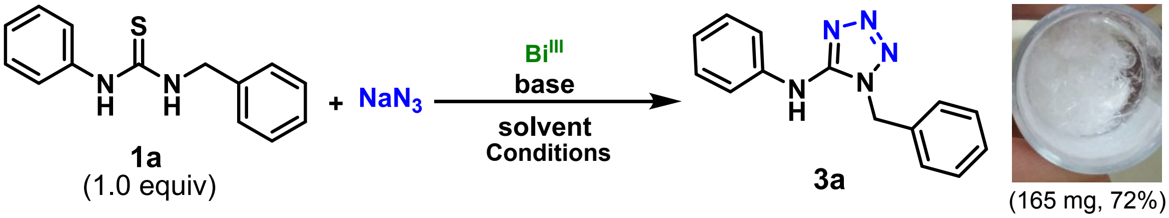

Entry	NaN_3 equiv	Bi^{III} salt	Bi^{III} salt equiv	Solvent	Temp (ºC)	Time (min)	Base	Base equiv.	Yield (%)[a]
1	3	$Bi(NO_3)_3$	1	CH_3CN	rt	1440	Et_3N	3	trace
2	3	$Bi(NO_3)_3$	1	CH_3CN	reflux	1440	Et_3N	3	59
**3**	**3**	**$Bi(NO_3)_3$**	**1**	**CH_3CN**	**125 [b]**	**20**	**Et_3N**	**3**	**72**
4	2	$Bi(NO_3)_3$	1	CH_3CN	125 [b]	20	Et_3N	3	36
5	1	$Bi(NO_3)_3$	1	CH_3CN	125 [b]	20	Et_3N	3	trace
6	3	$Bi(NO_3)_3$	0.5	CH_3CN	125 [b]	20	Et_3N	3	13
7	3	$Bi(NO_3)_3$	1	H_2O	125 [b]	20	Et_3N	3	none[d]
8	3	$Bi(NO_3)_3$	1	H_2O	125 [b]	20	K_2CO_3	3	none[d]
9	3	$Bi(NO_3)_3$	1	DMF	125 [b]	15	Et_3N	3	69
10	3	BiI_3	0.5	CH_3CN	125 [b]	20	Et_3N	3	trace
11	3	BiI_3	1	CH_3CN	125 [b]	20	Et_3N	3	none[e]
12	3	$Bi(NO_3)_3$	1	CH_3CN	125 [b]	20	Et_3N	1	21
13	3	$Bi(NO_3)_3$	1	CH_3CN	125 [b]	20	Et_3N	2	47

[a]Isolated yield; [b]Microwave heating; [c]3.0 equiv. [d]Urea formed; [e]Recovered reagents

NMR, HRMS, and IR Spectra

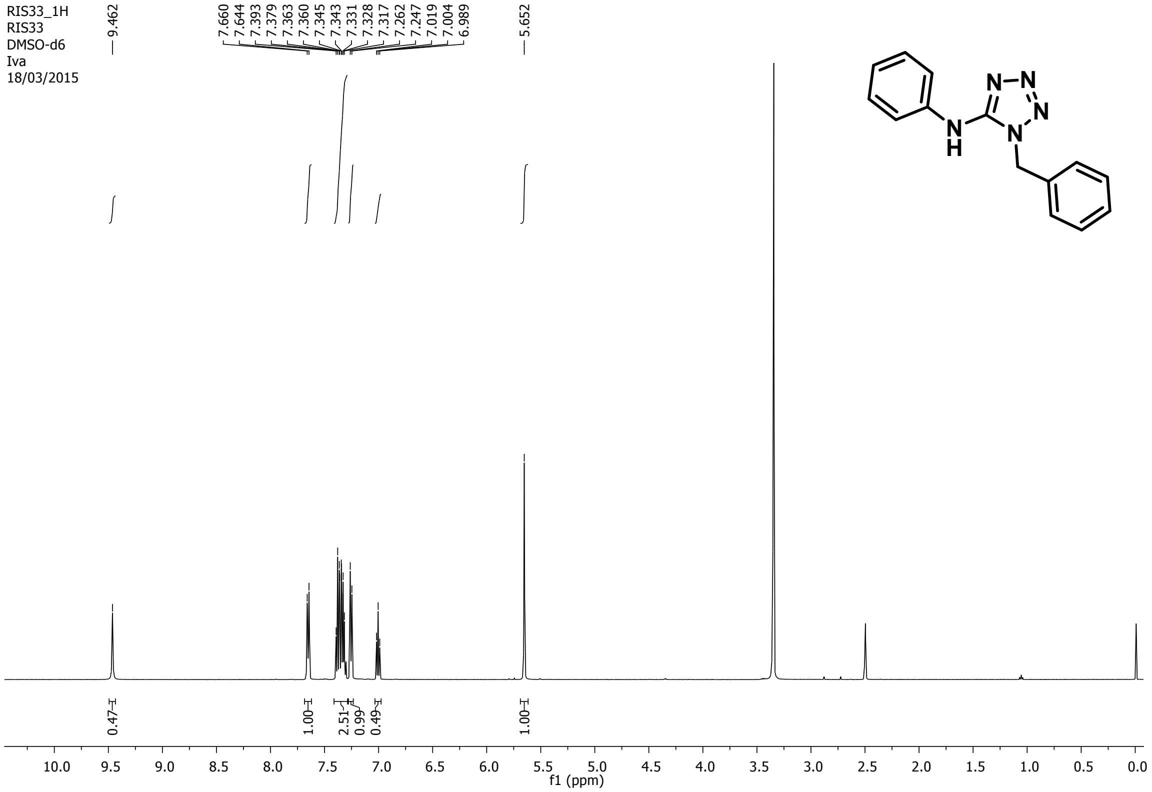

^{1}H NMR (DMSO-d_6, 500 MHz) of 3a.

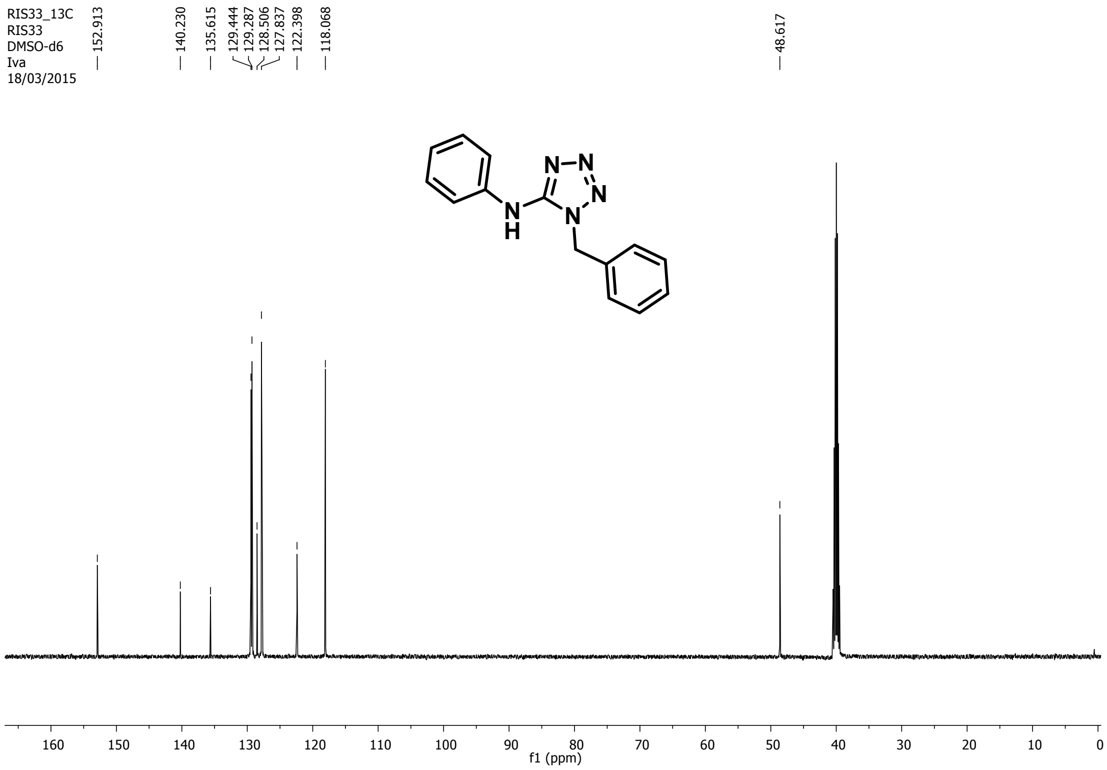

$^{13}C\{^{1}H\}$ NMR (DMSO-d_6, 125 MHz) of 3a

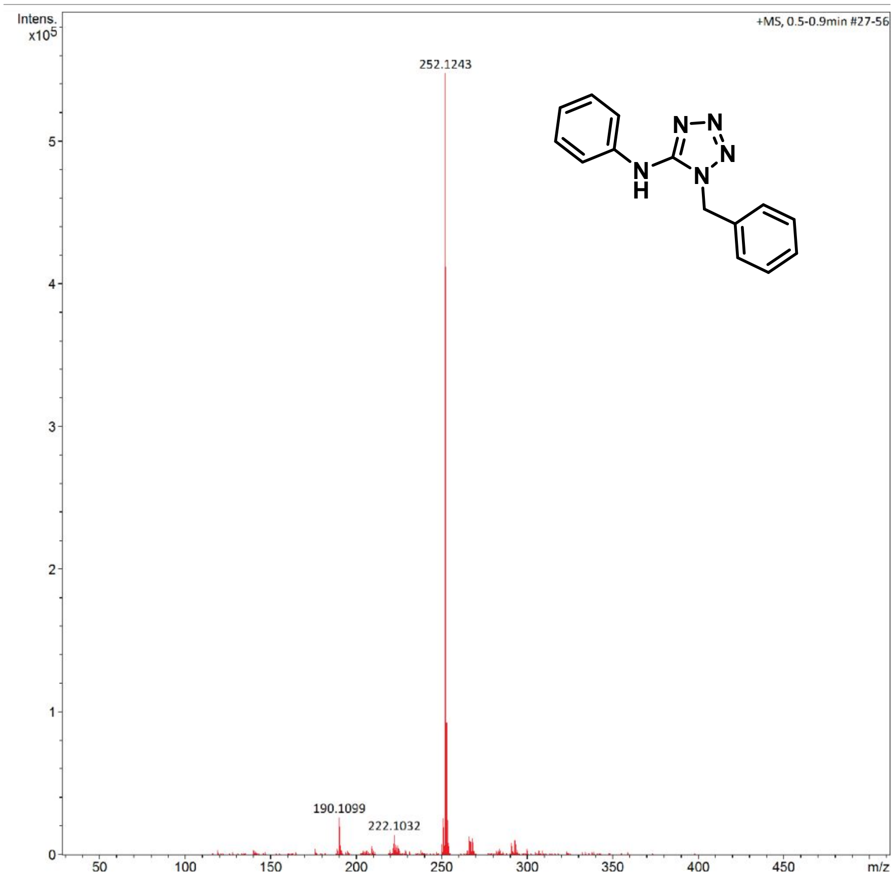

HRMS spectrum of 3a

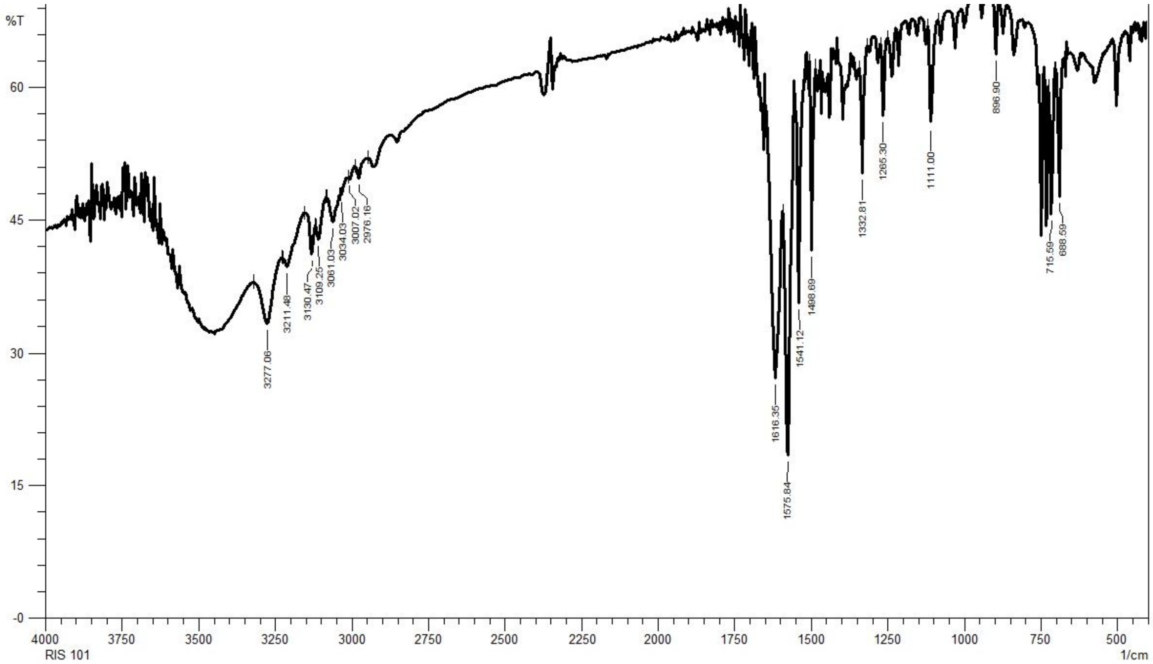

IR spectrum (KBr) of 3a.

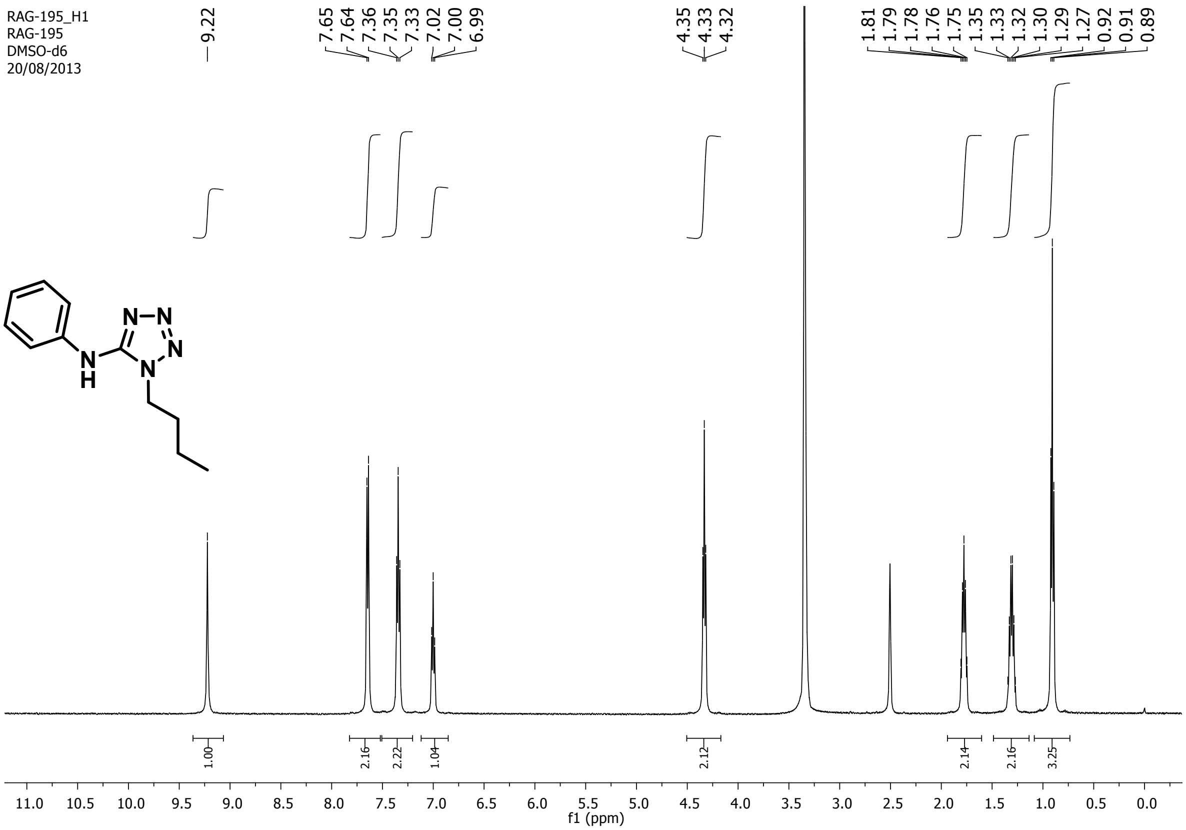

^{1}H NMR (DMSO-d_6, 500 MHz) of 3b.

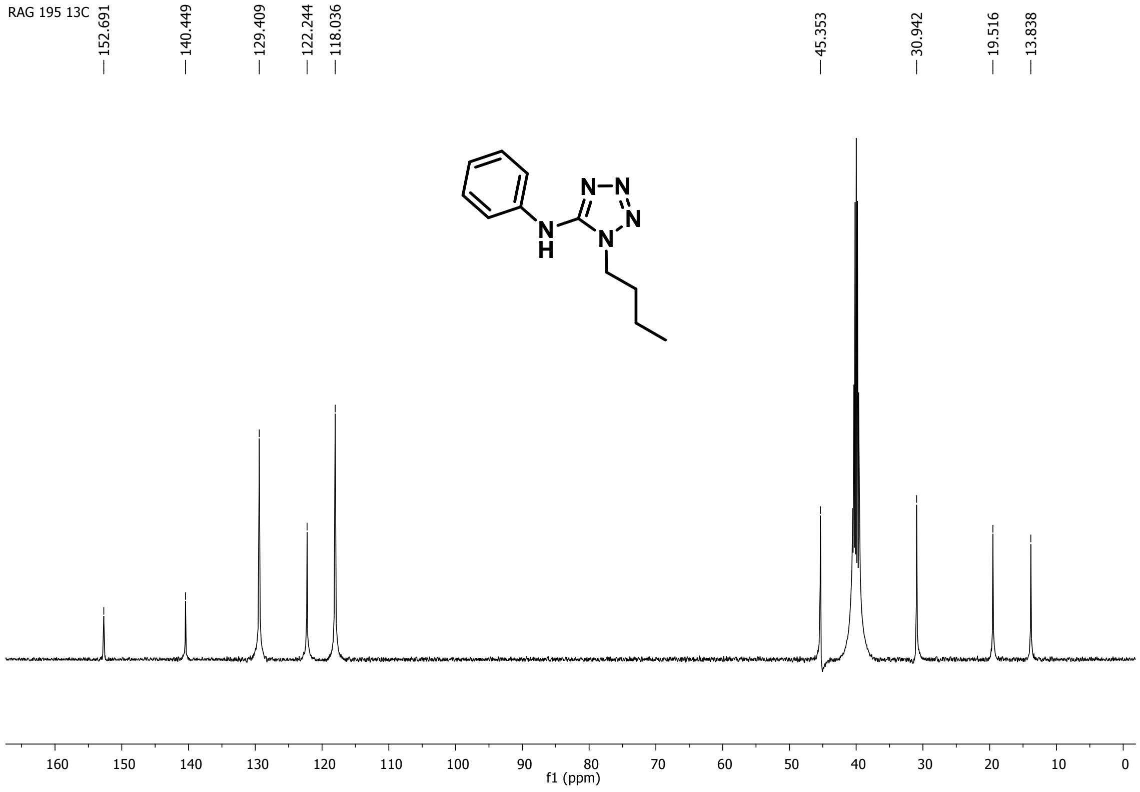

$^{13}C\{^{1}H\}$ NMR (DMSO-d_6, 125 MHz) of 3b

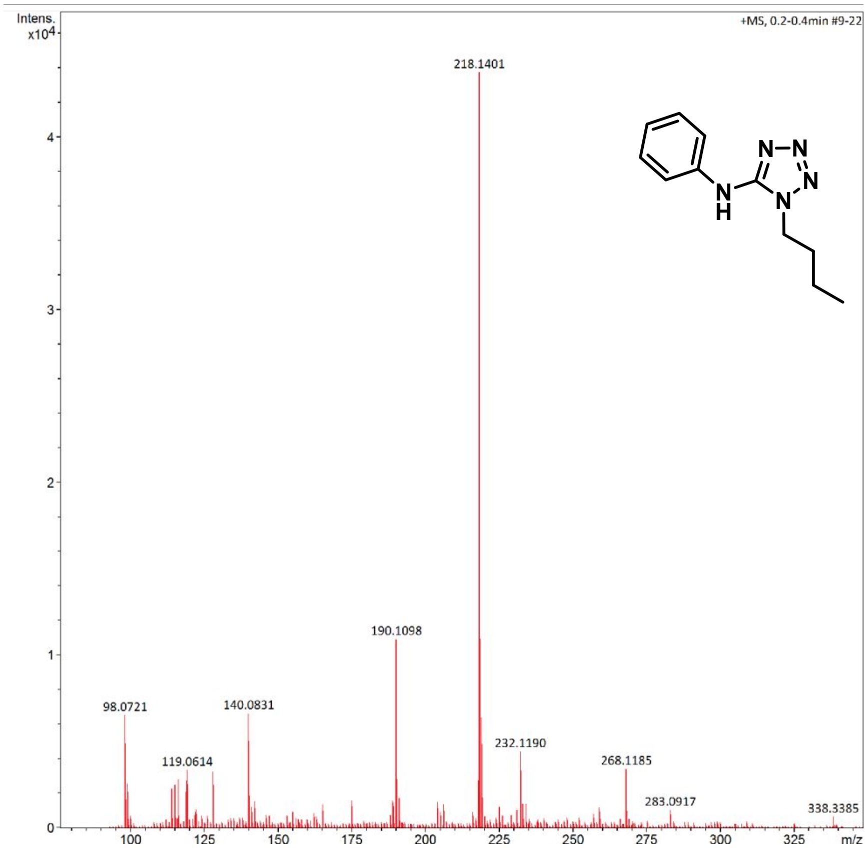

HRMS spectrum of 3b.

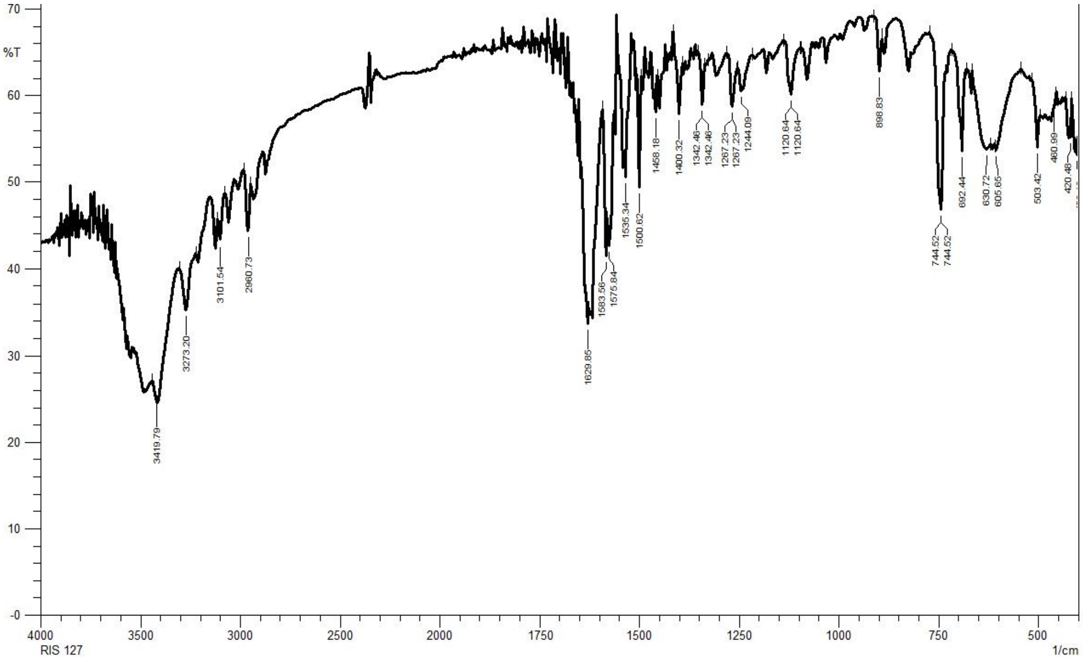

IR spectrum (KBr) of 3b.

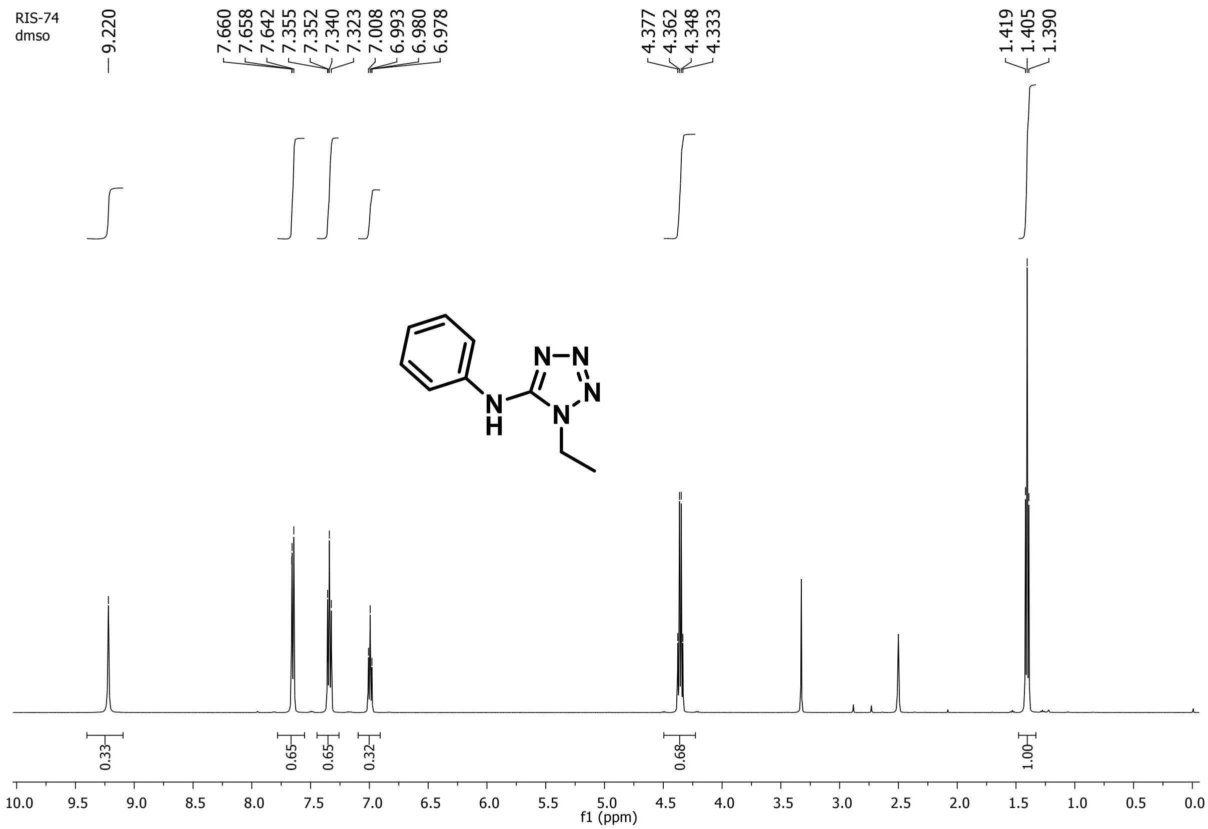

^{1}H NMR (DMSO-d_6, 500 MHz) of 3c.

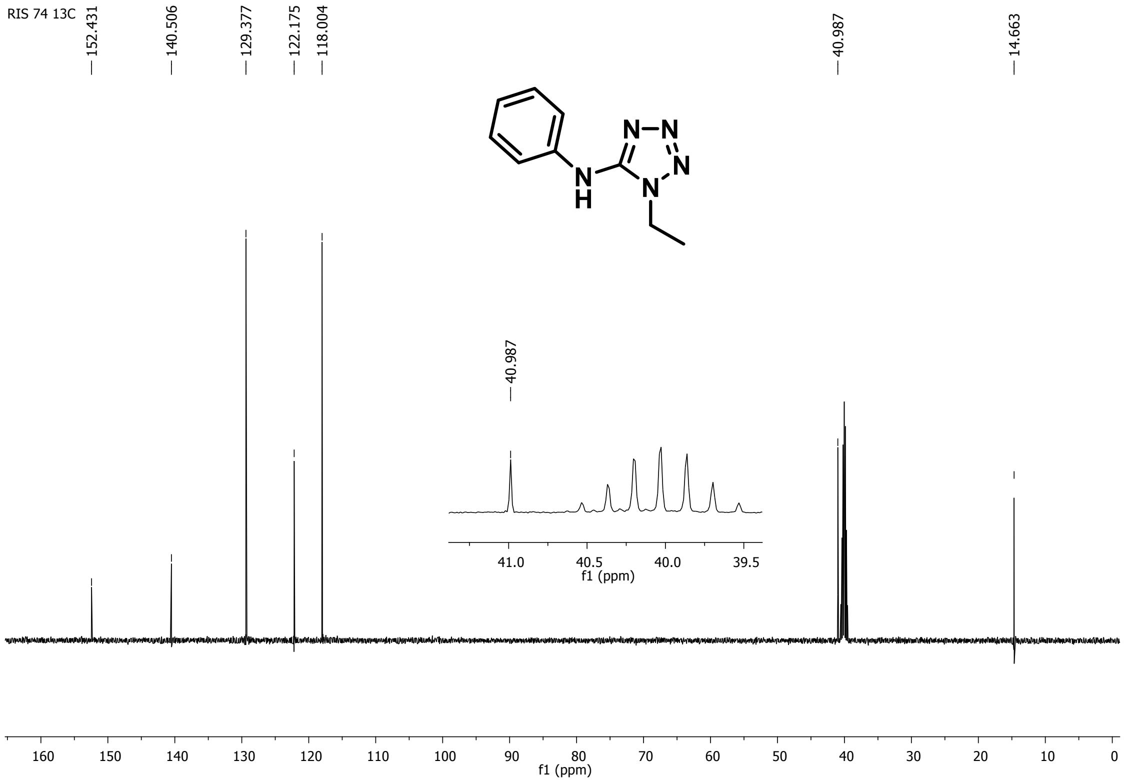

$^{13}C\{^{1}H\}$ NMR (DMSO-d_6, 125 MHz) of 3c.

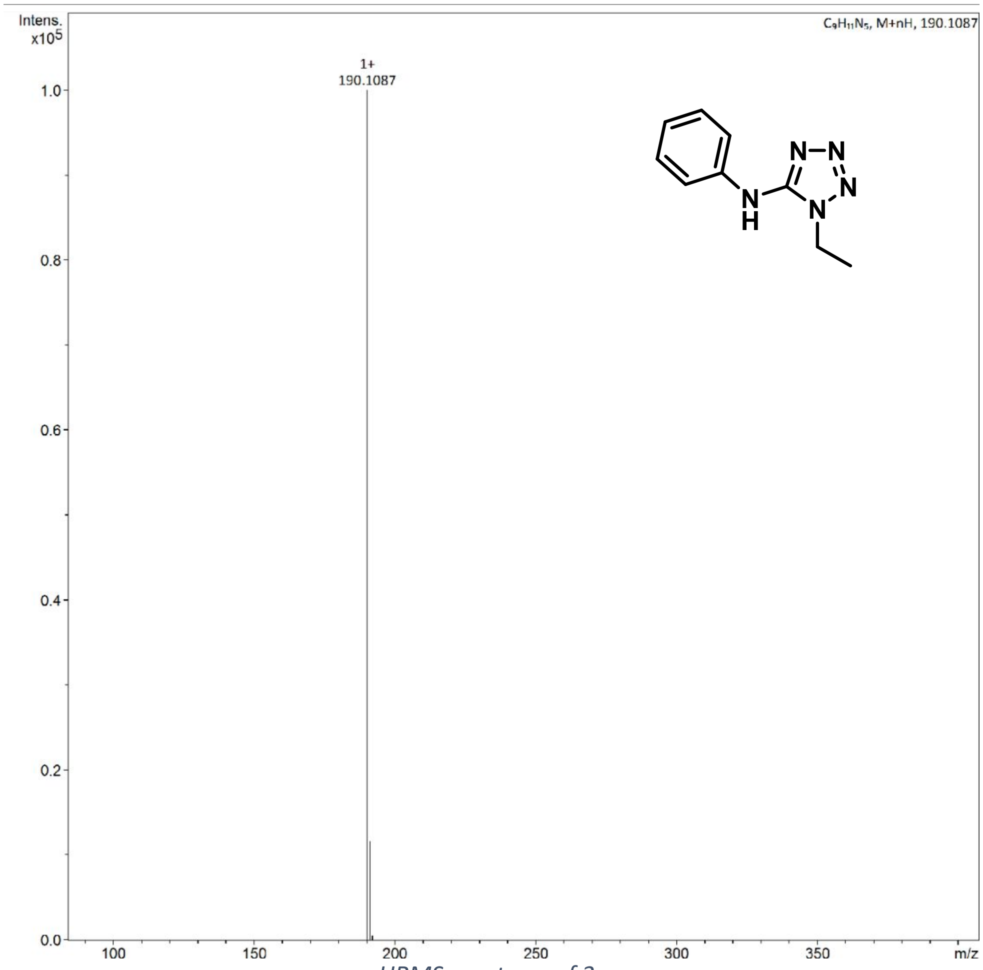

HRMS spectrum of 3c

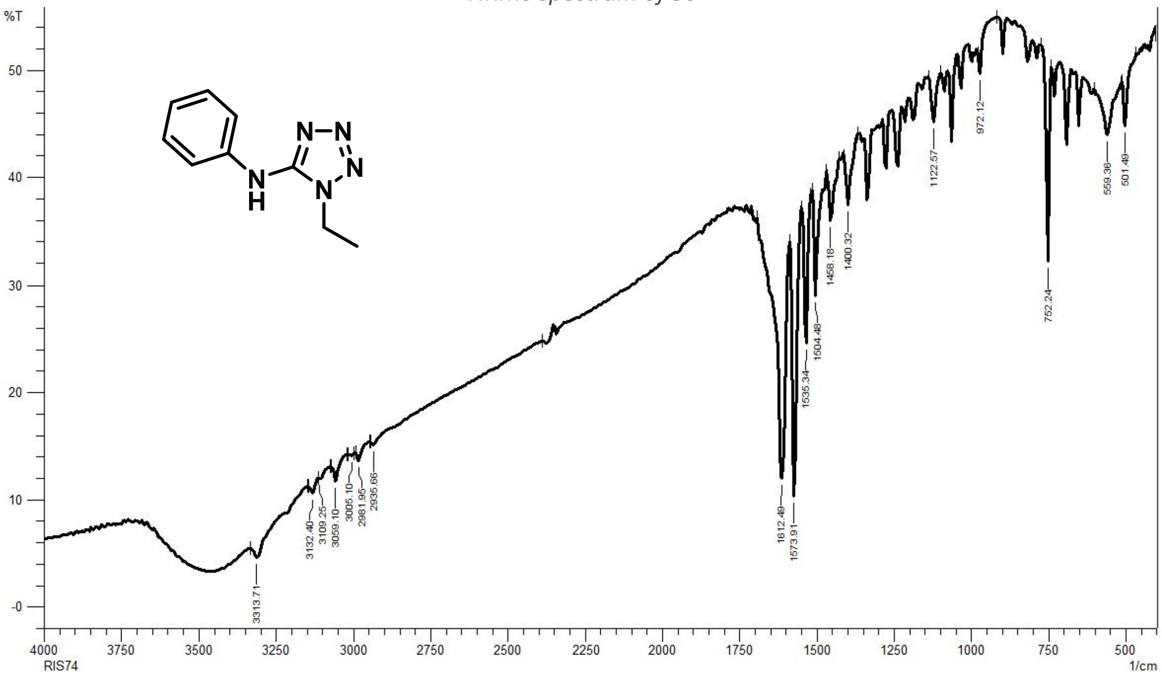

IR spectrum (KBr) of 3c

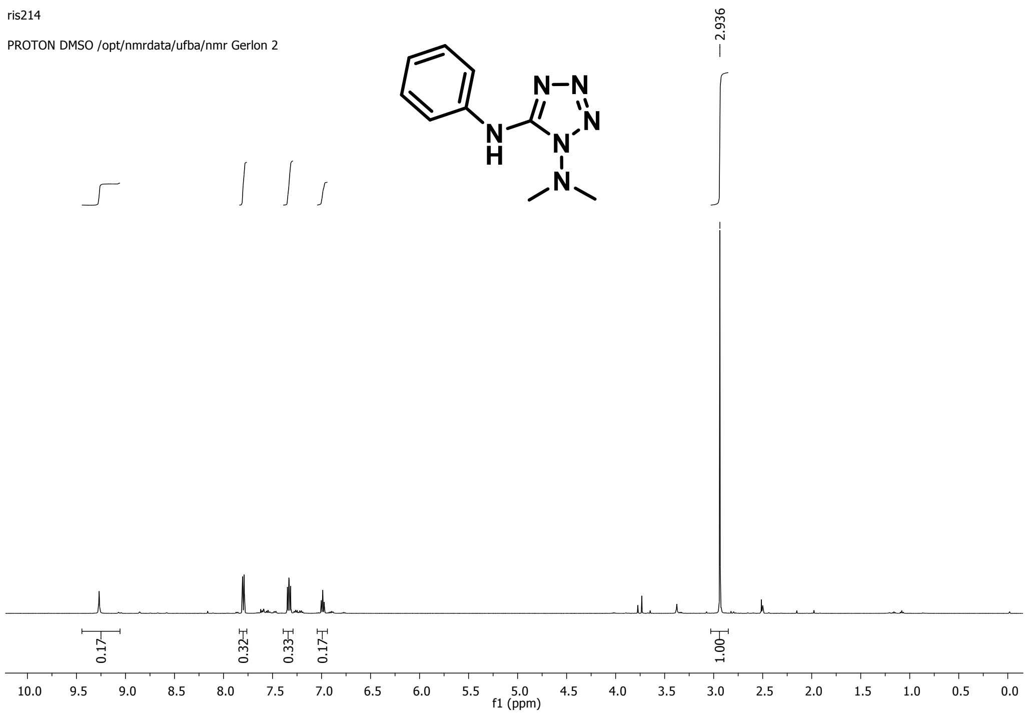

^{1}H NMR (DMSO-d_6, 500 MHz) of 3d.

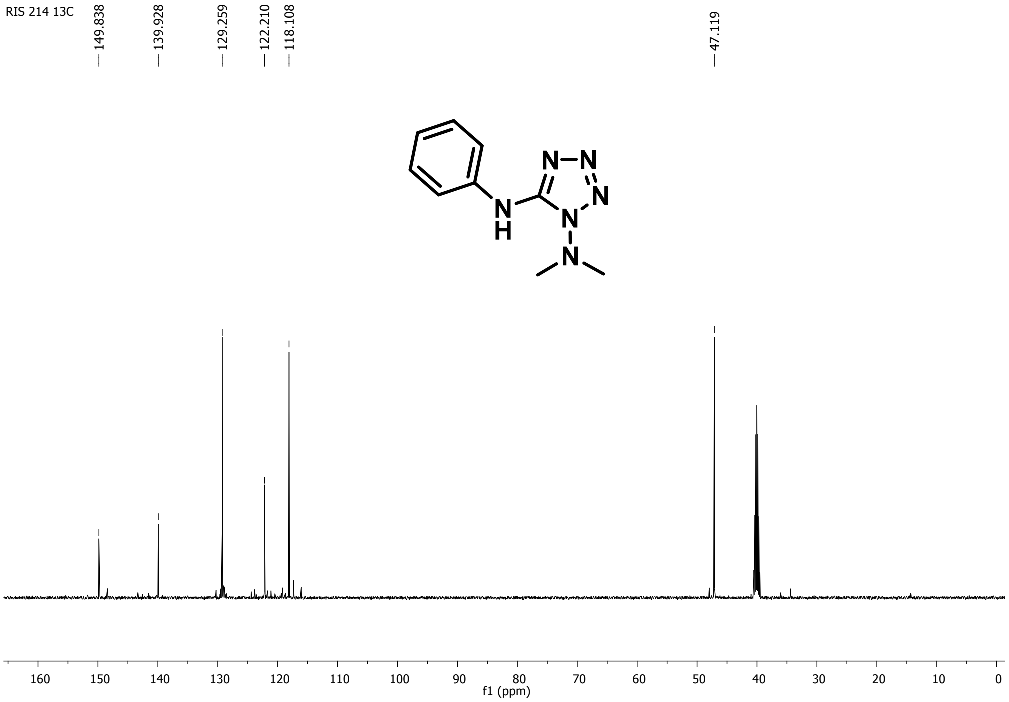

^{13}C{^{1}H} NMR (DMSO-d_6, 125 MHz) of 3d.

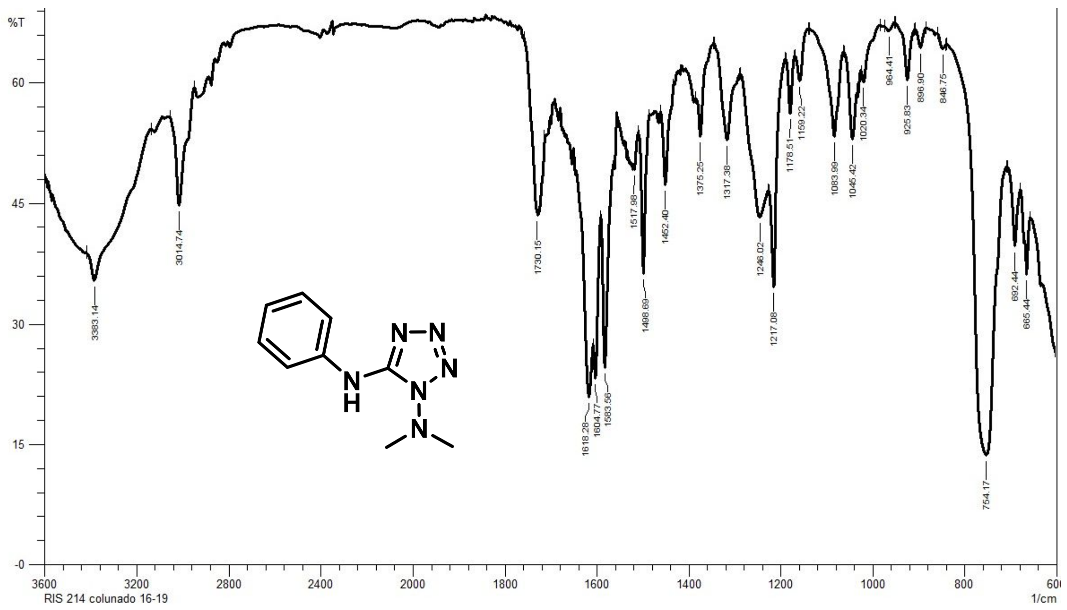

IR spectrum (KBr) of 3d

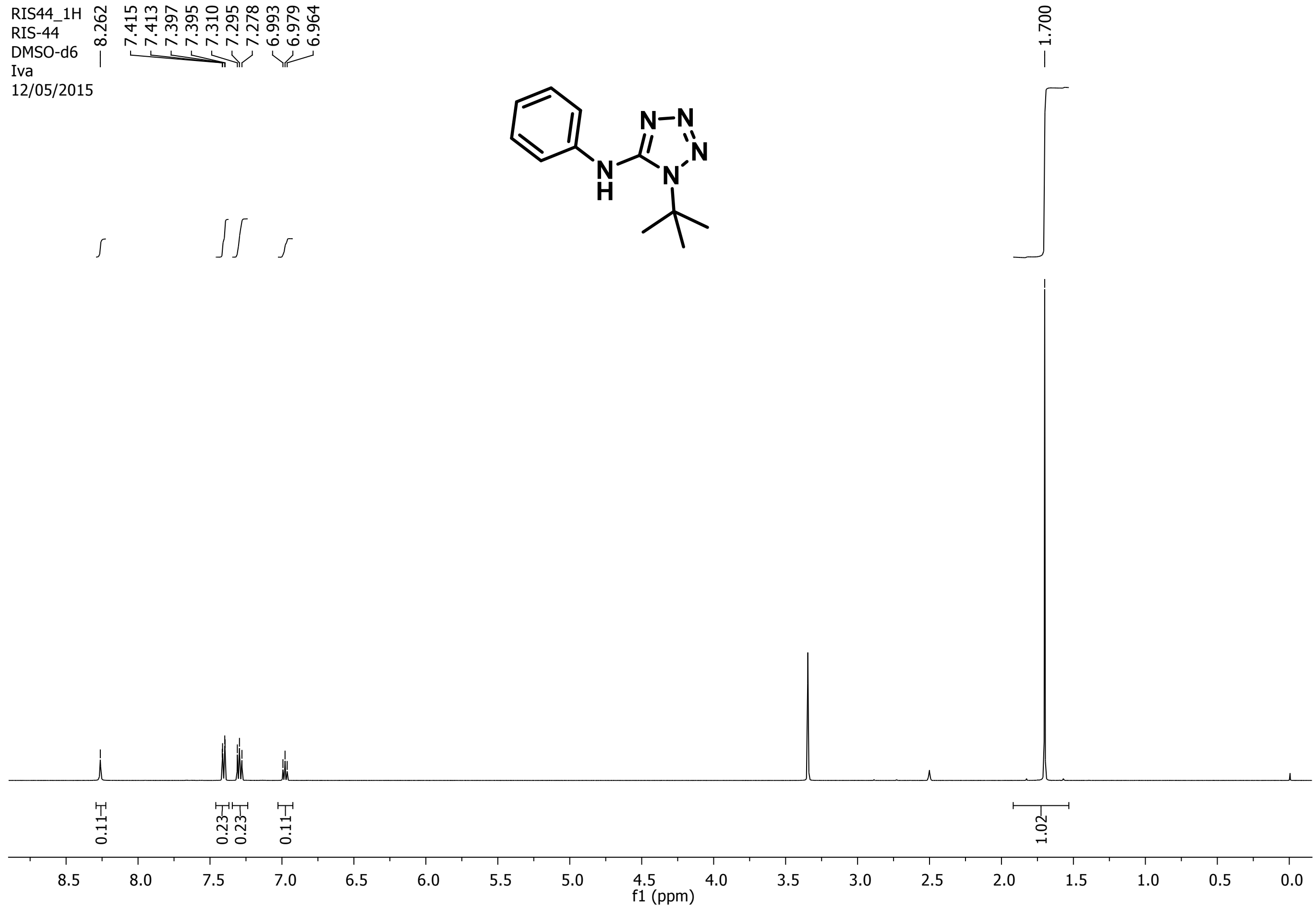

^{1}H NMR (DMSO-d_6, 500 MHz) of 3e.

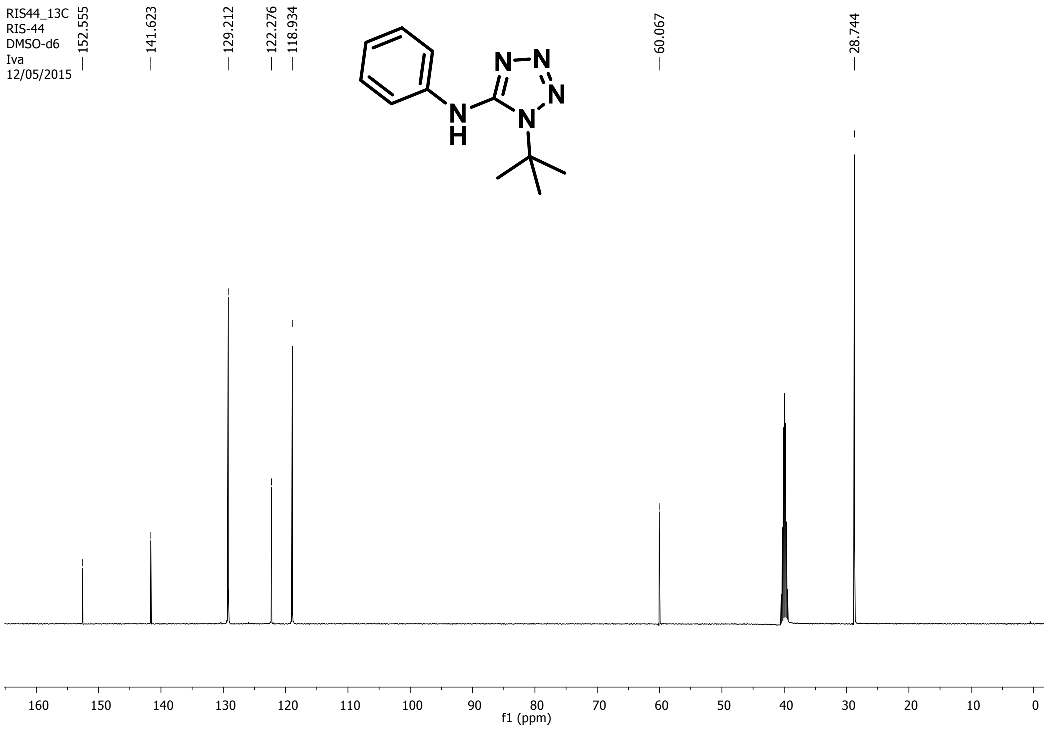

$^{13}C\{^{1}H\}$ NMR (DMSO-d_6, 125 MHz) of 3e.

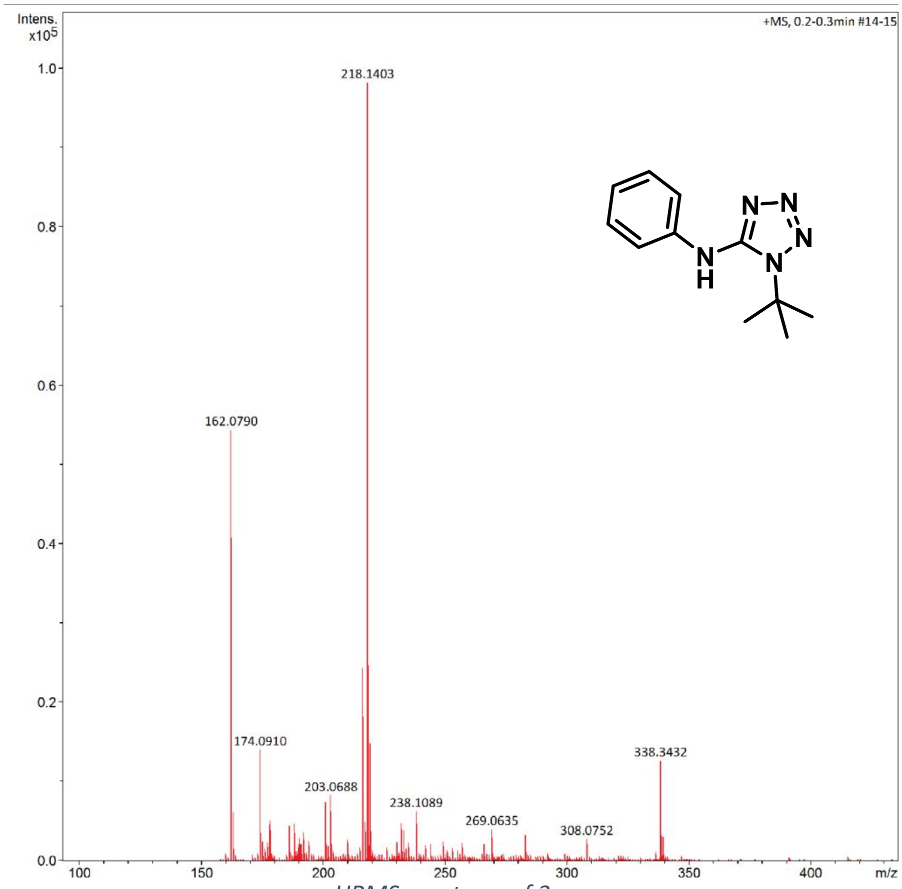

HRMS spectrum of 3e.

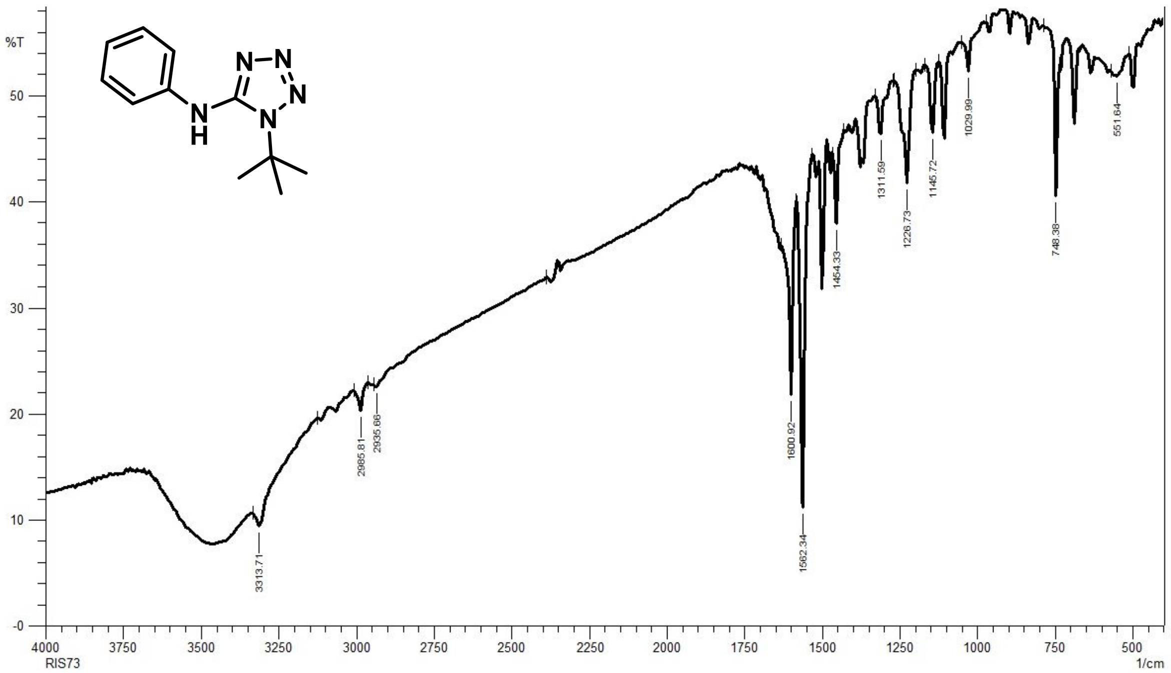

IR spectrum (KBr) of 3e.

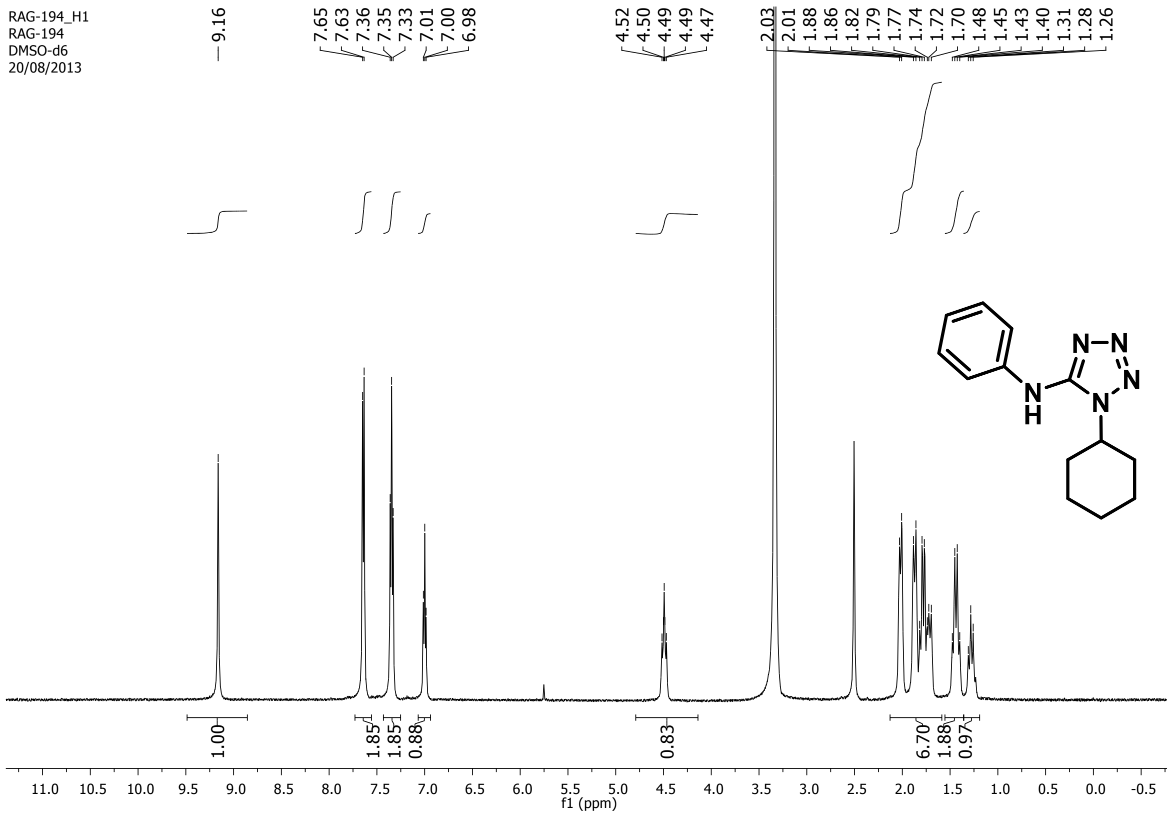

^{1}H NMR (DMSO-d_6, 500 MHz) of 3f.

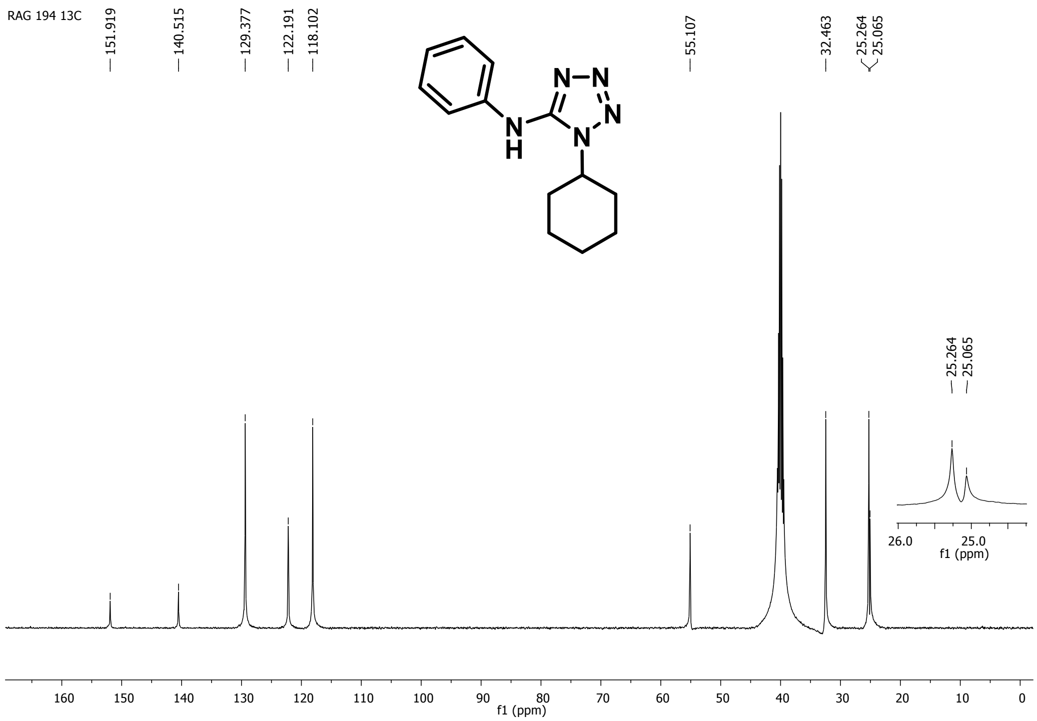

$^{13}C\{^{1}H\}$ NMR (DMSO-d_6, 125 MHz) of 3f.

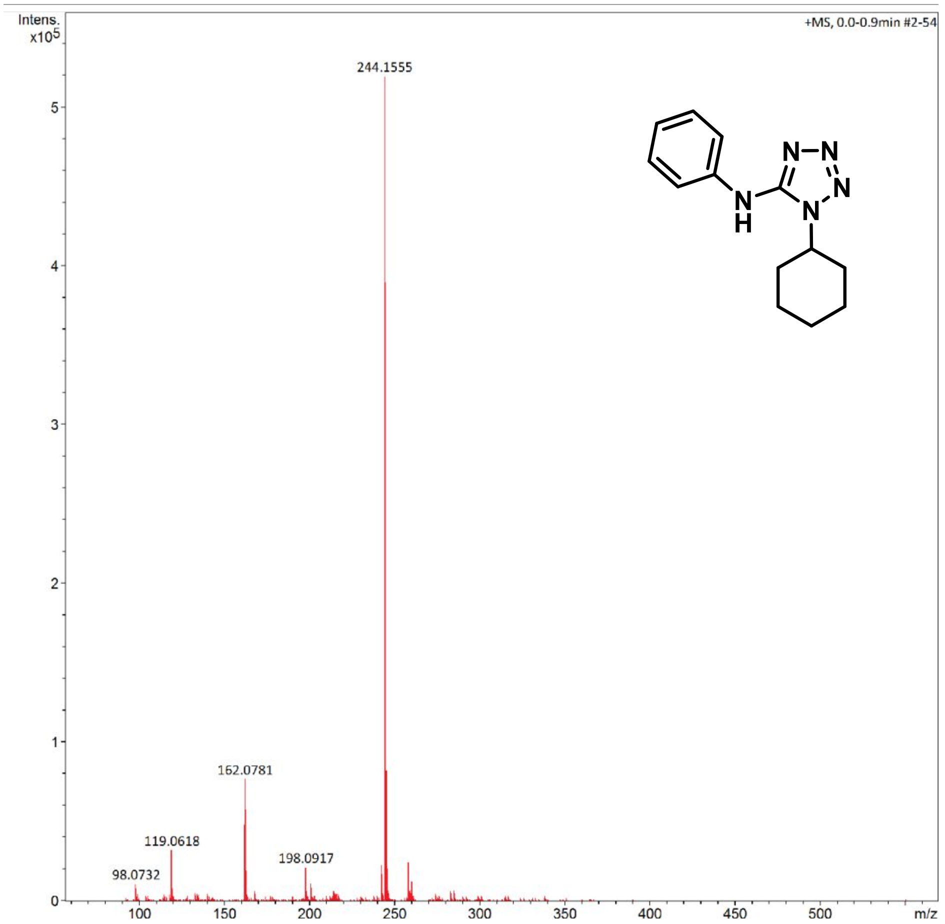

HRMS spectrum of 3f.

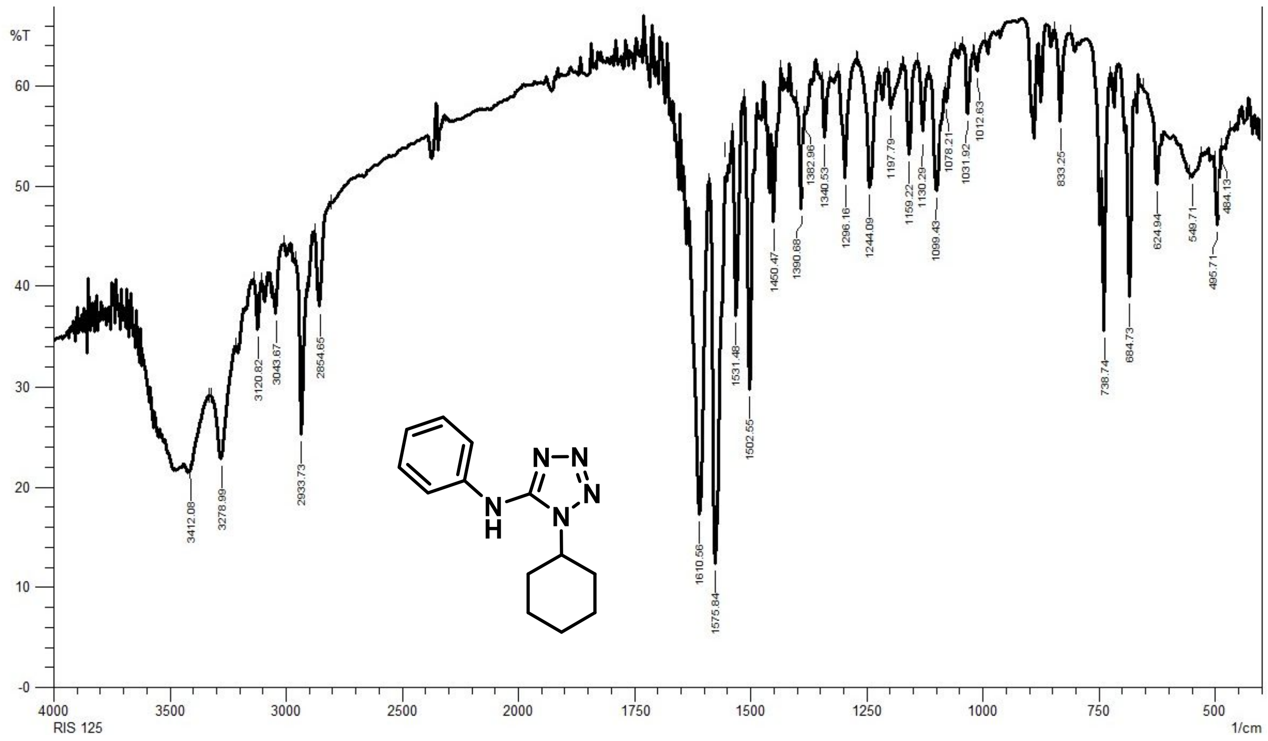

IR spectrum (KBr) of 3f.

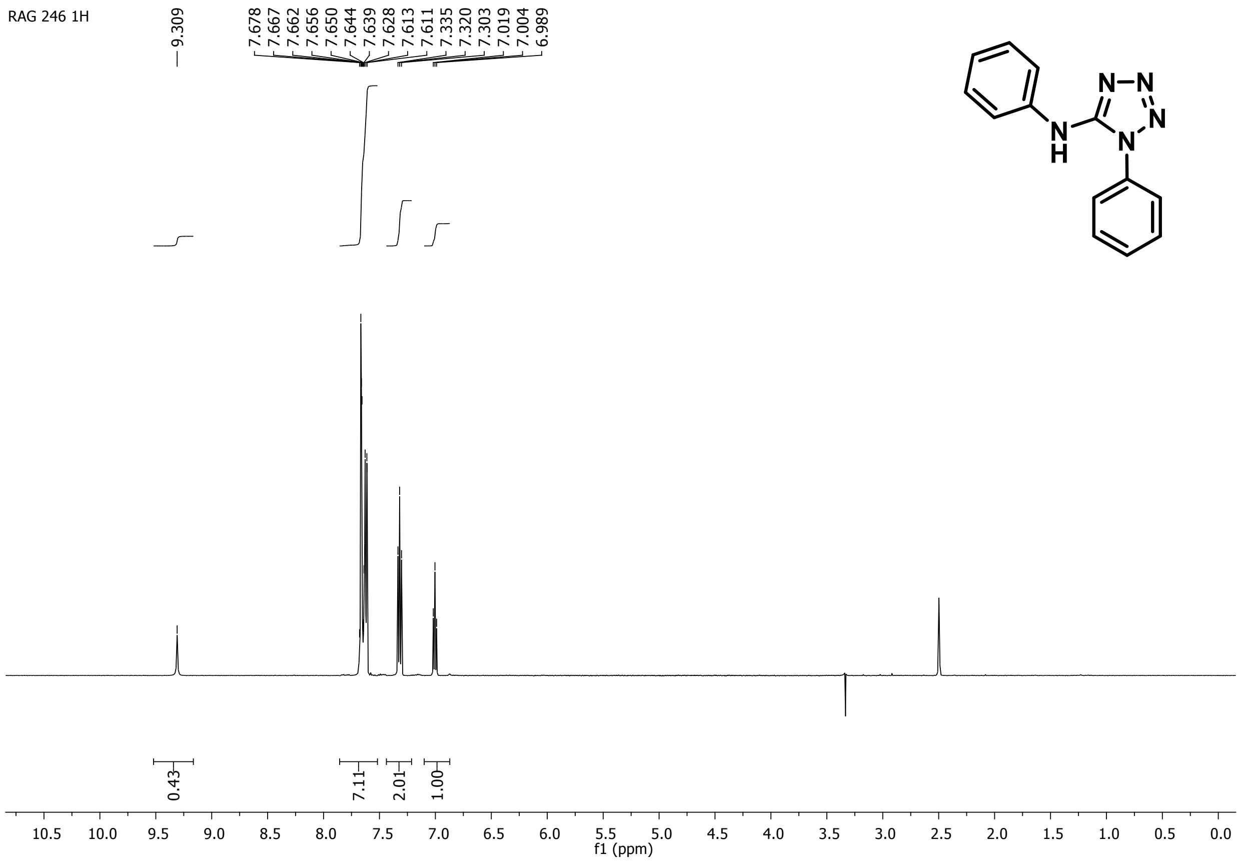

^1H NMR (DMSO-d_6, 500 MHz) of 3g.

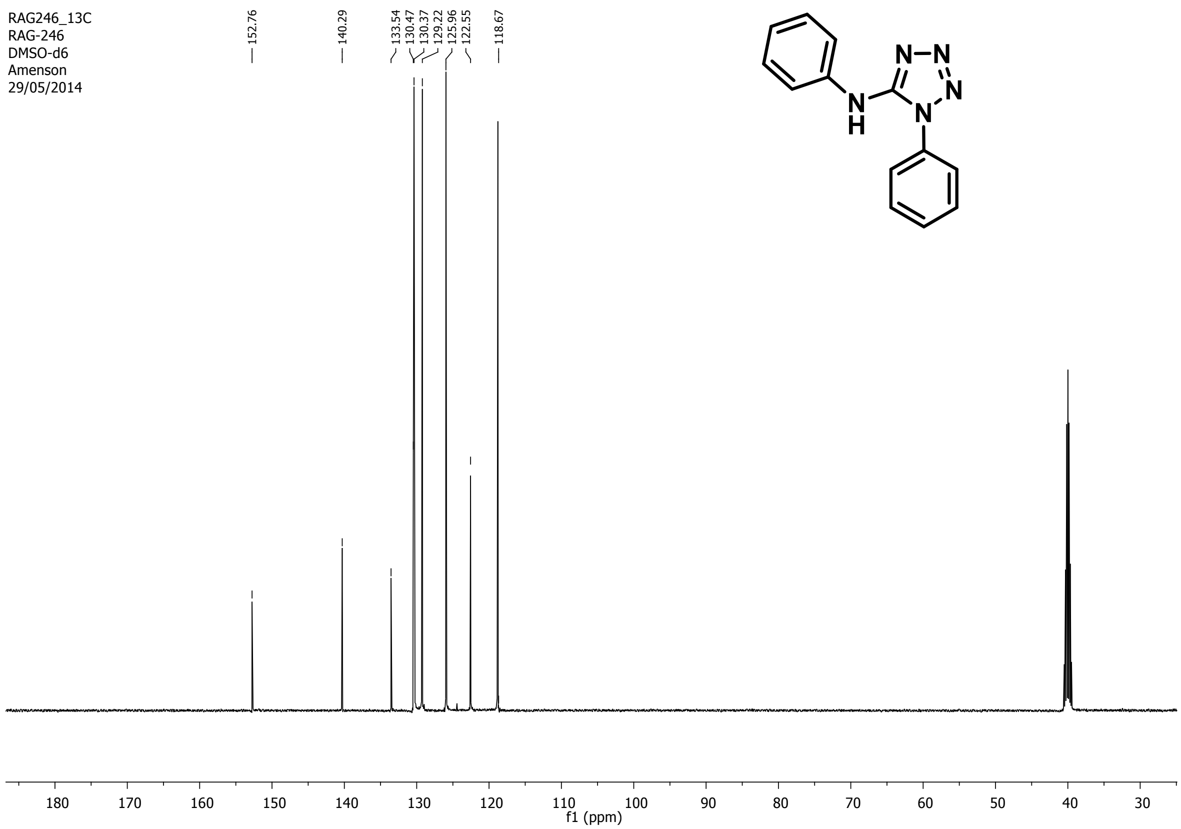

$^{13}C\{^1H\}$ *NMR (DMSO-d_6, 125 MHz) of 3g.*

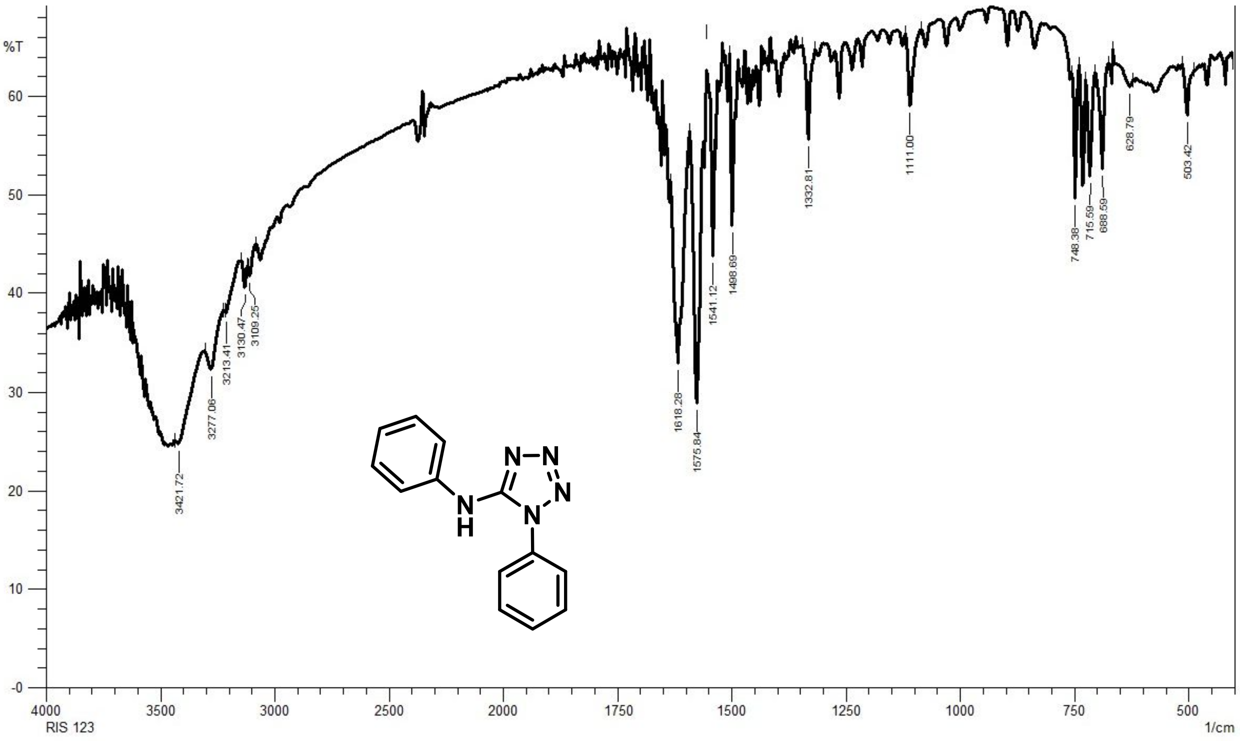

IR spectrum (KBr) of 3g.

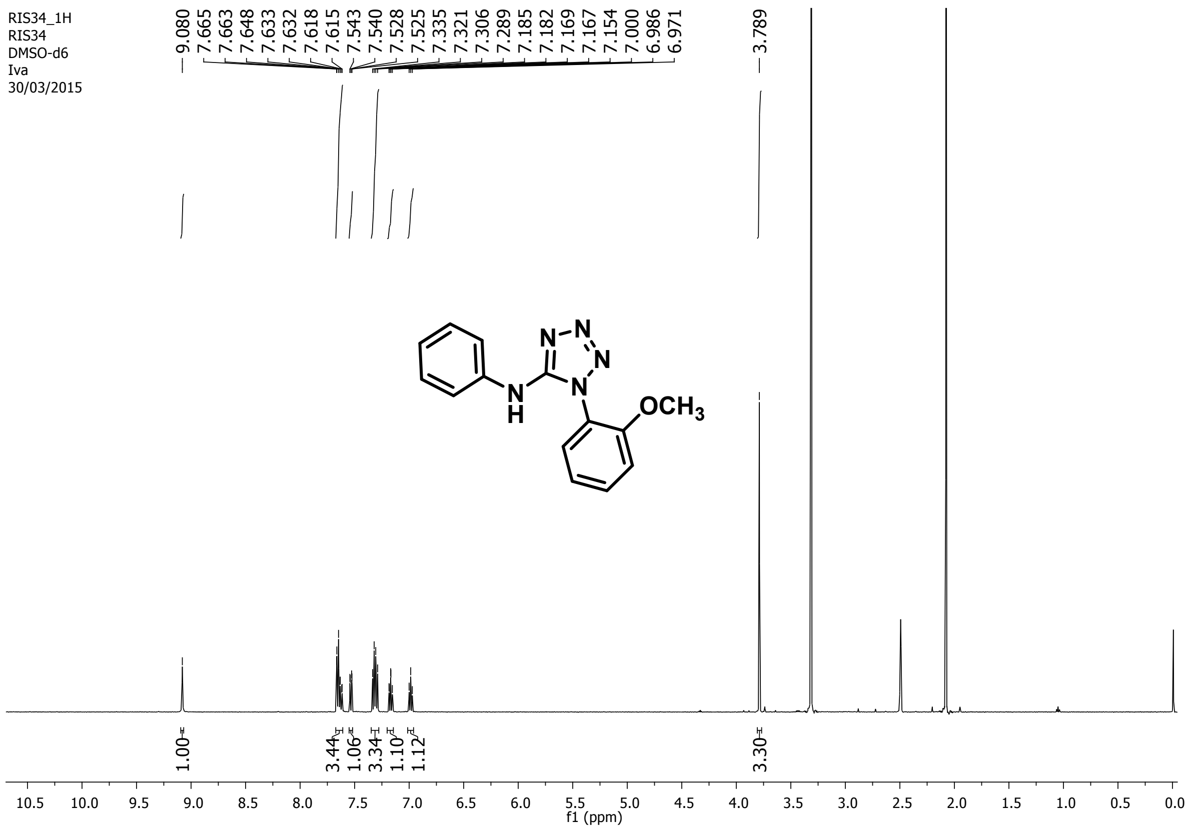

^{1}H NMR (DMSO-d_6, 500 MHz) of 3h.

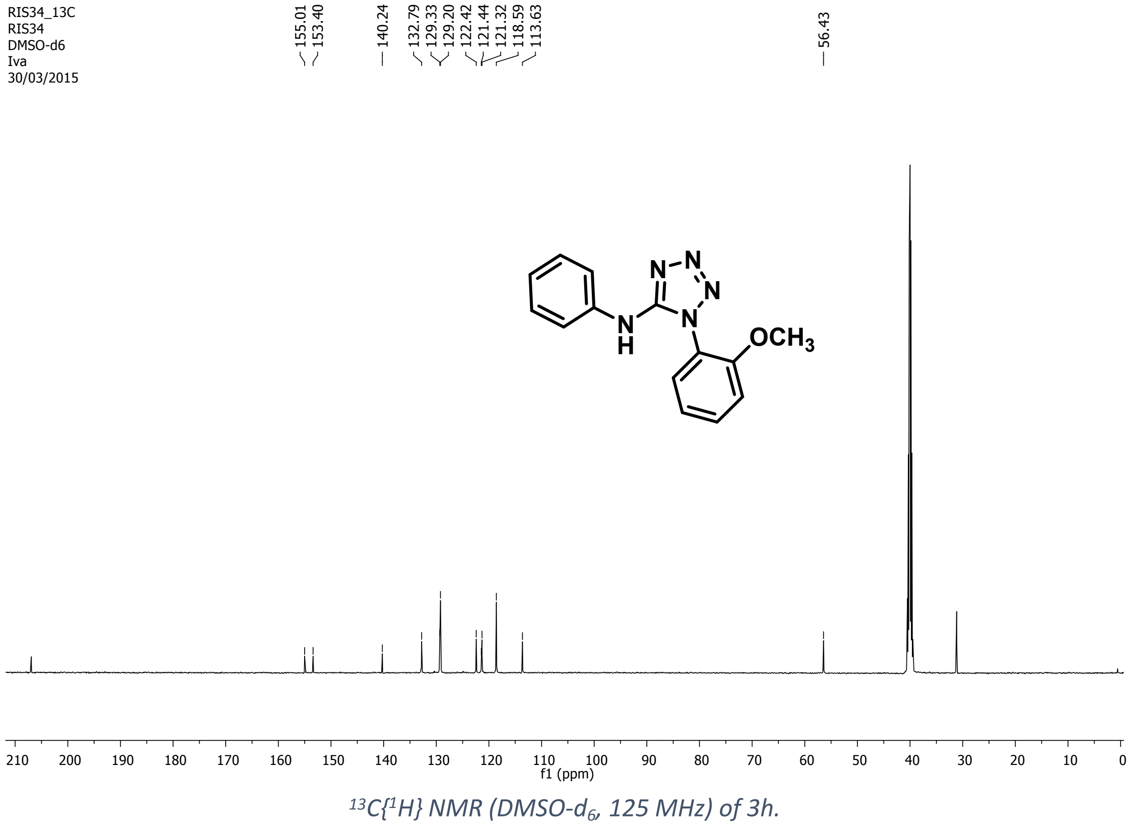

$^{13}C\{^{1}H\}$ NMR (DMSO-d_6, 125 MHz) of 3h.

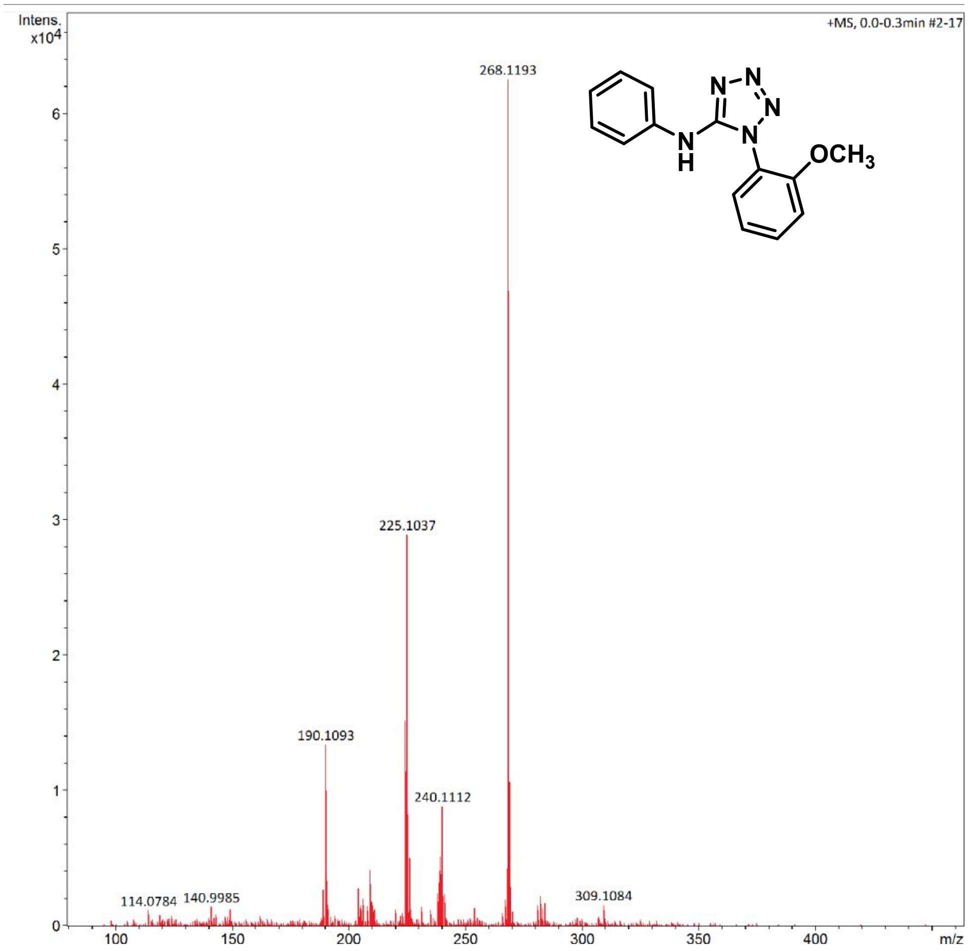

HRMS spectrum of 3h.

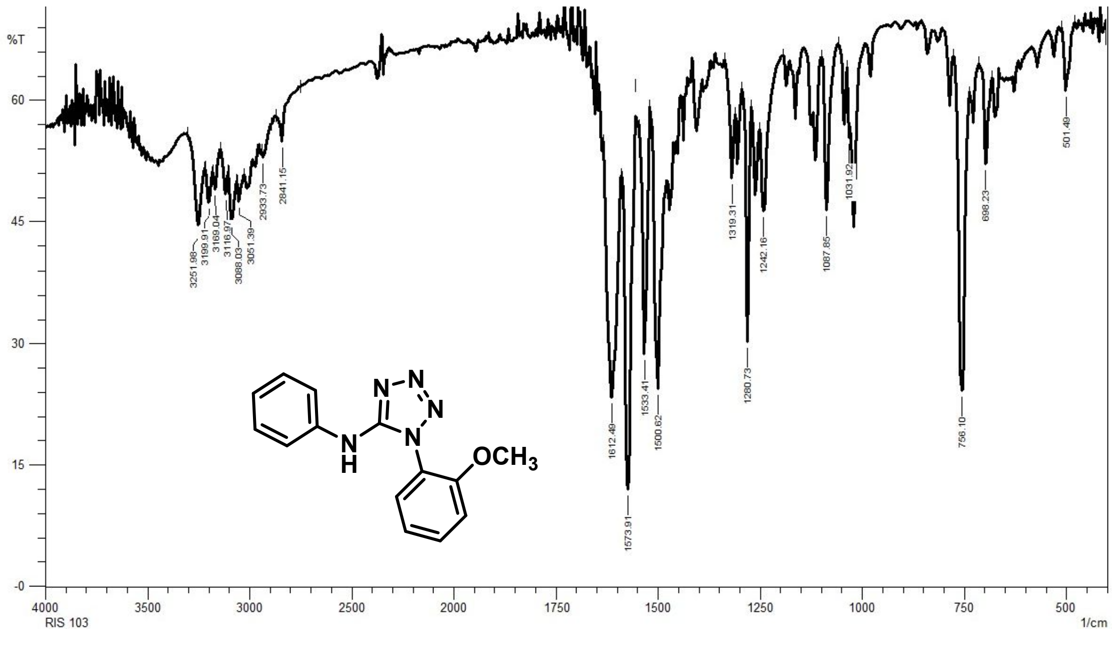

IR spectrum (KBr) of 3h.

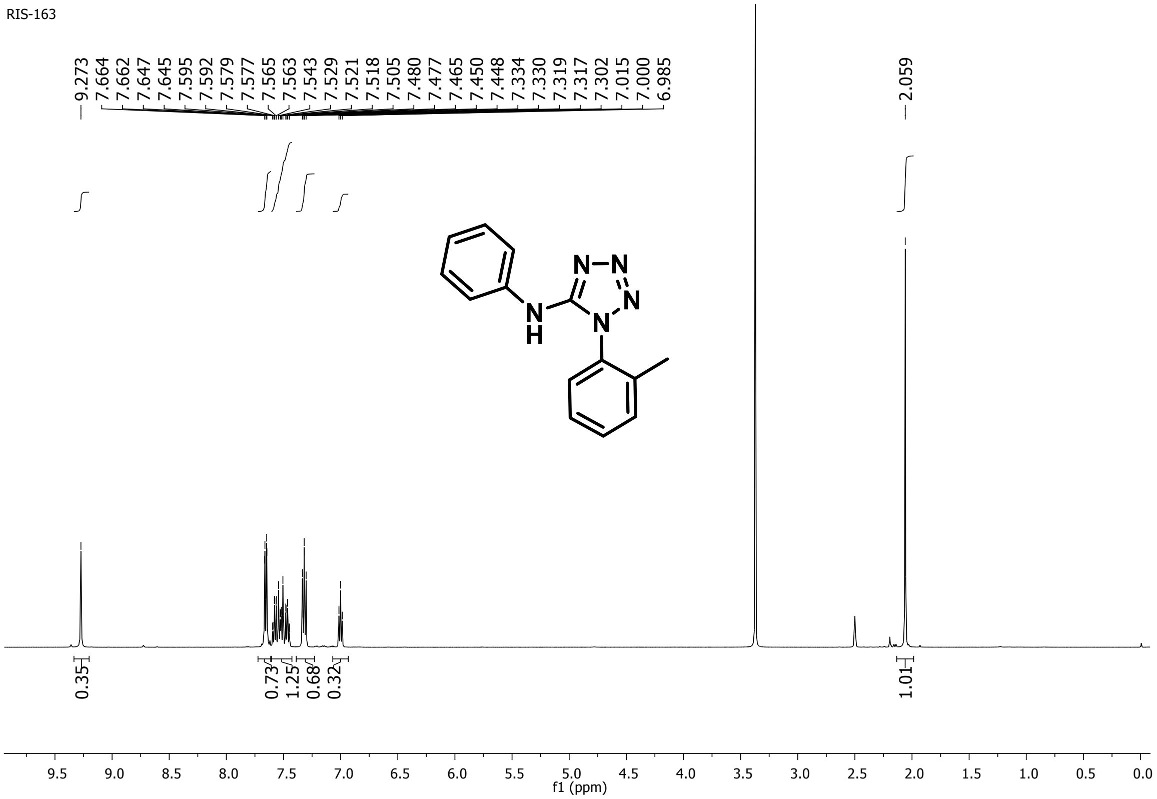

¹H NMR (DMSO-d_6, 500 MHz) of 3i.

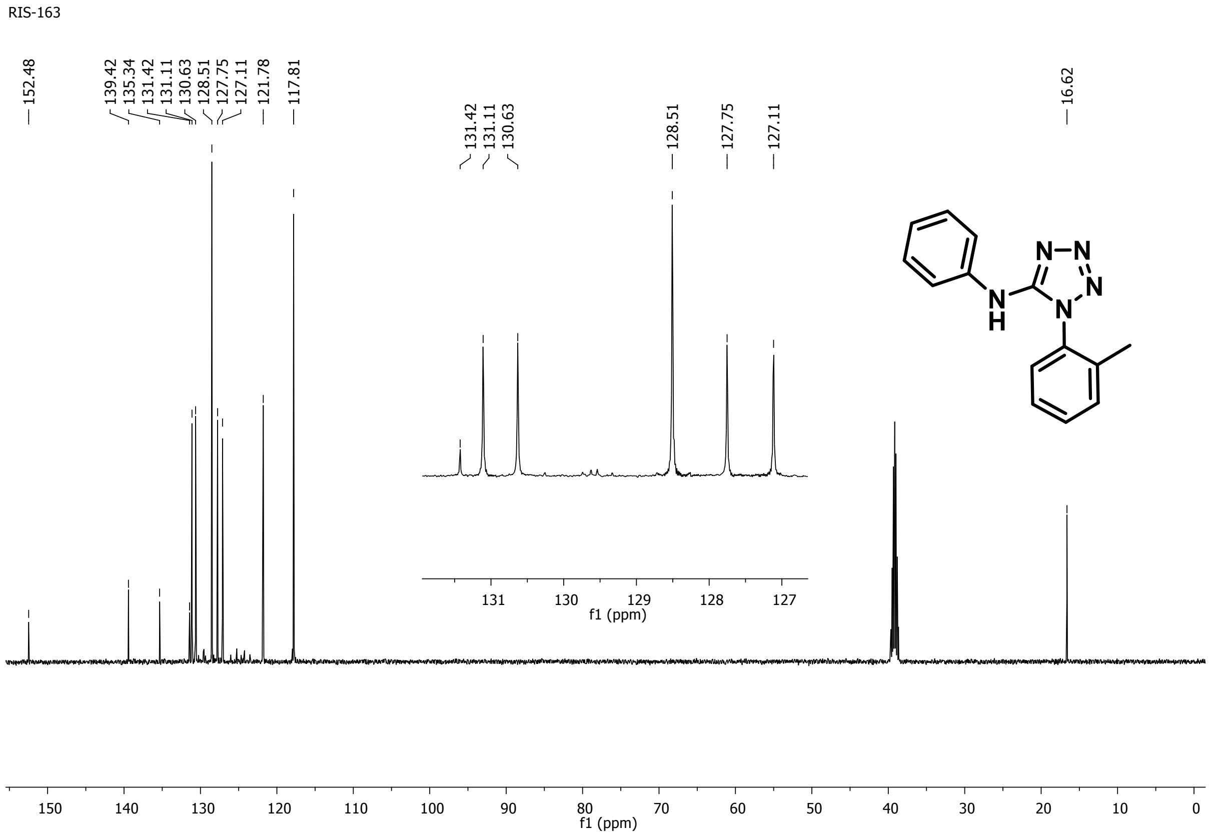

¹³C{¹H} NMR (DMSO-d_6, 125 MHz) of 3i.

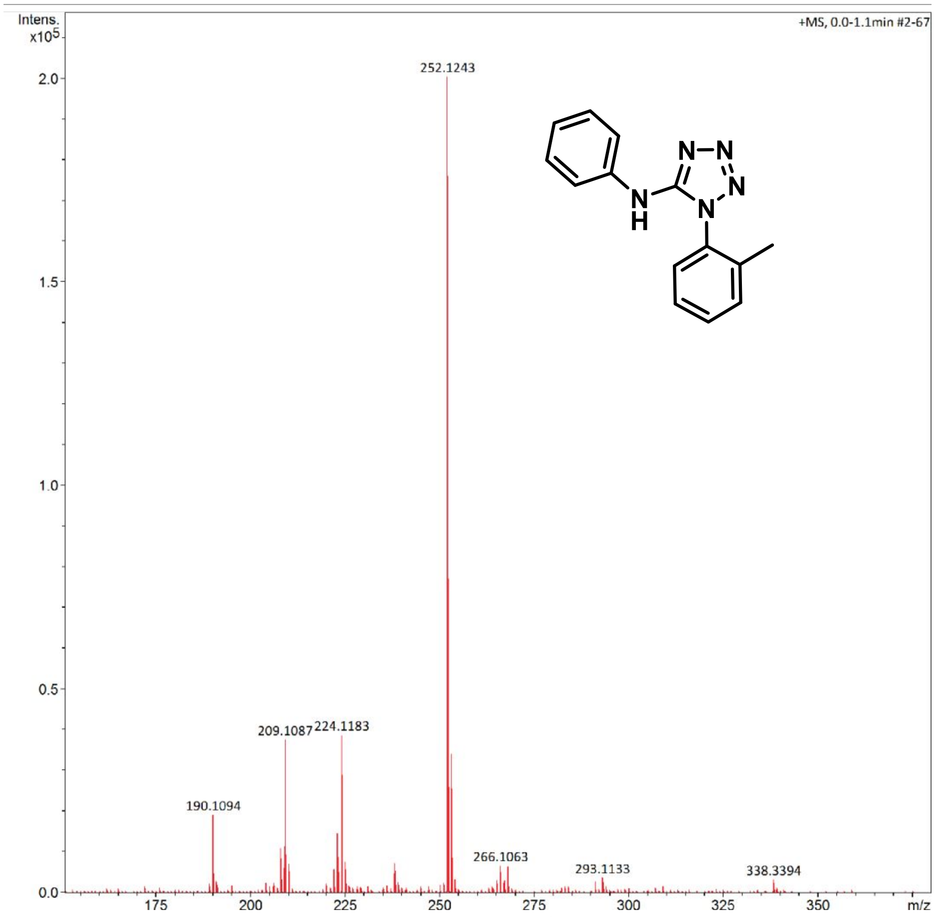

HRMS spectrum of 3i.

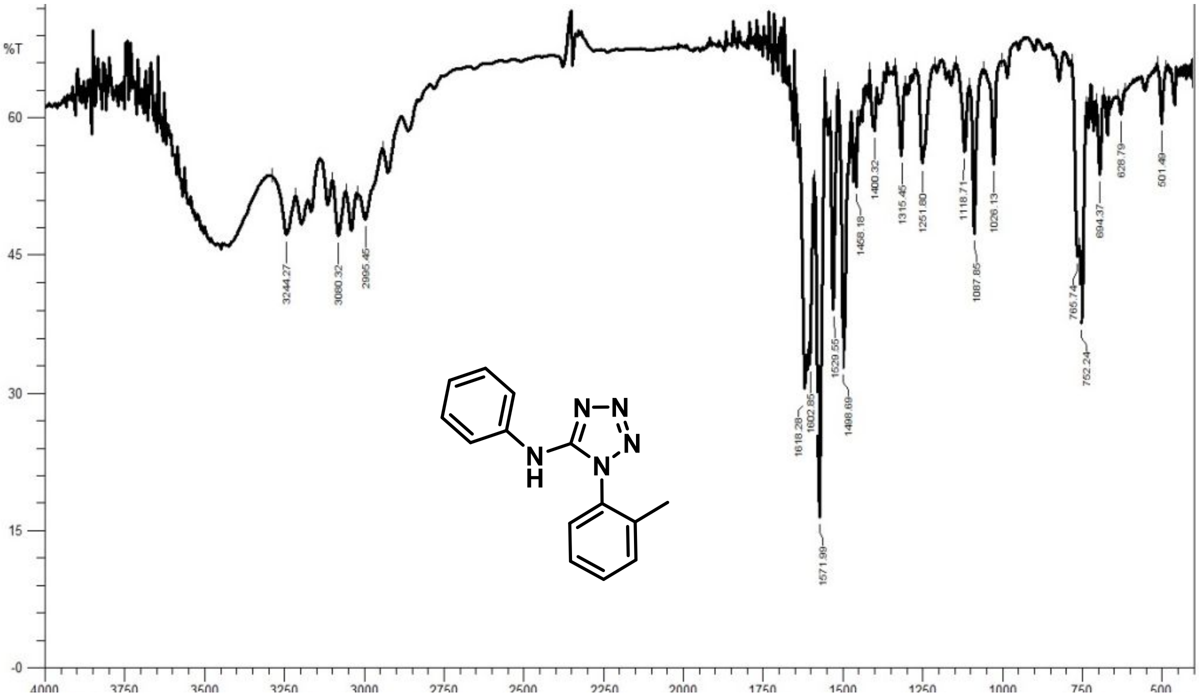

IR spectrum (KBr) of 3i.

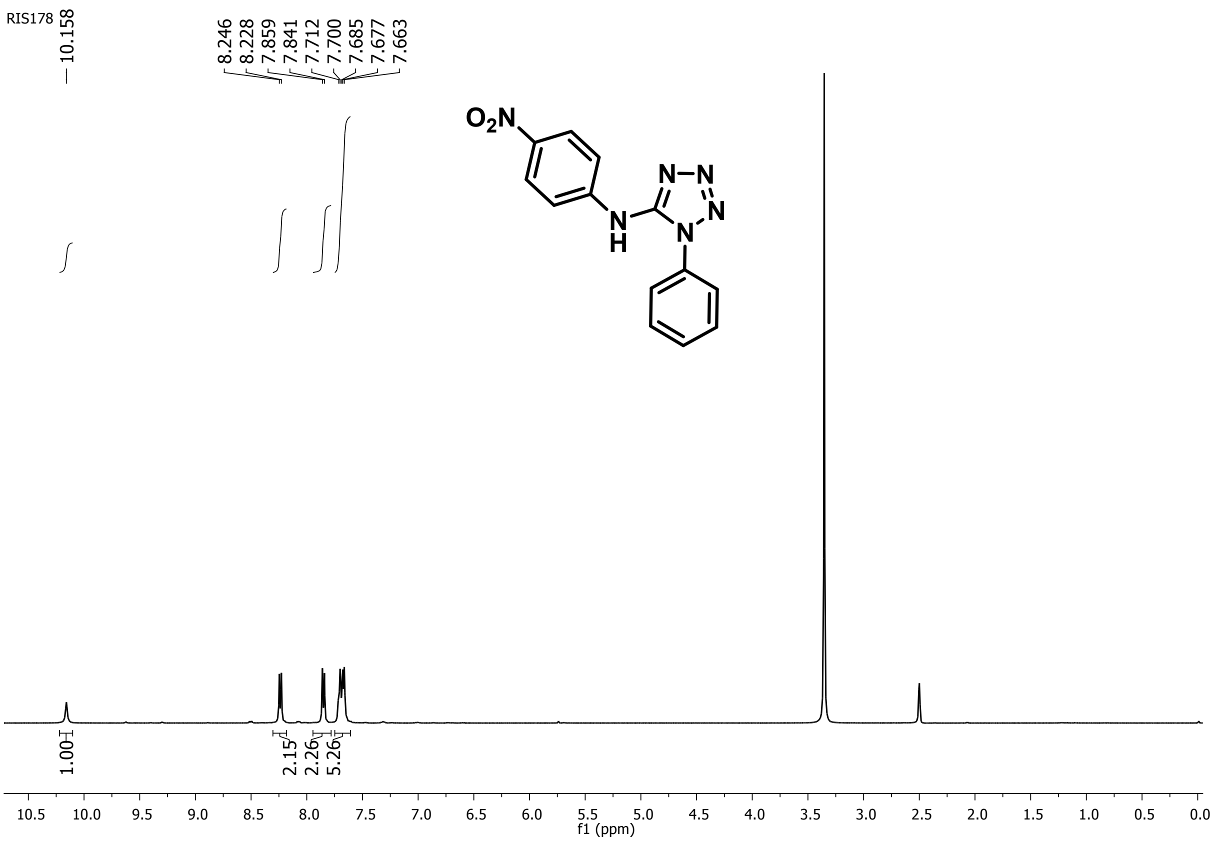

^{1}H NMR (DMSO-d_6, 500 MHz) of 3j'.

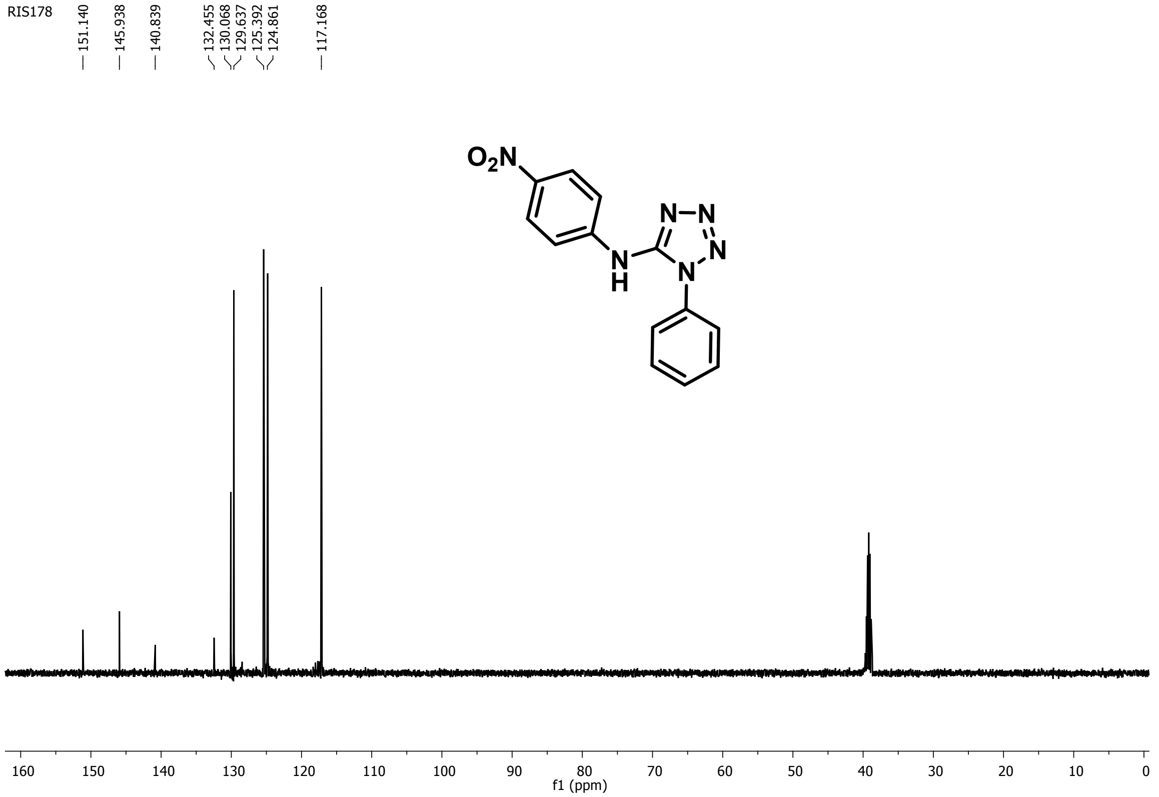

$^{13}C\{^{1}H\}$ NMR (DMSO-d_6, 125 MHz) of 3j'

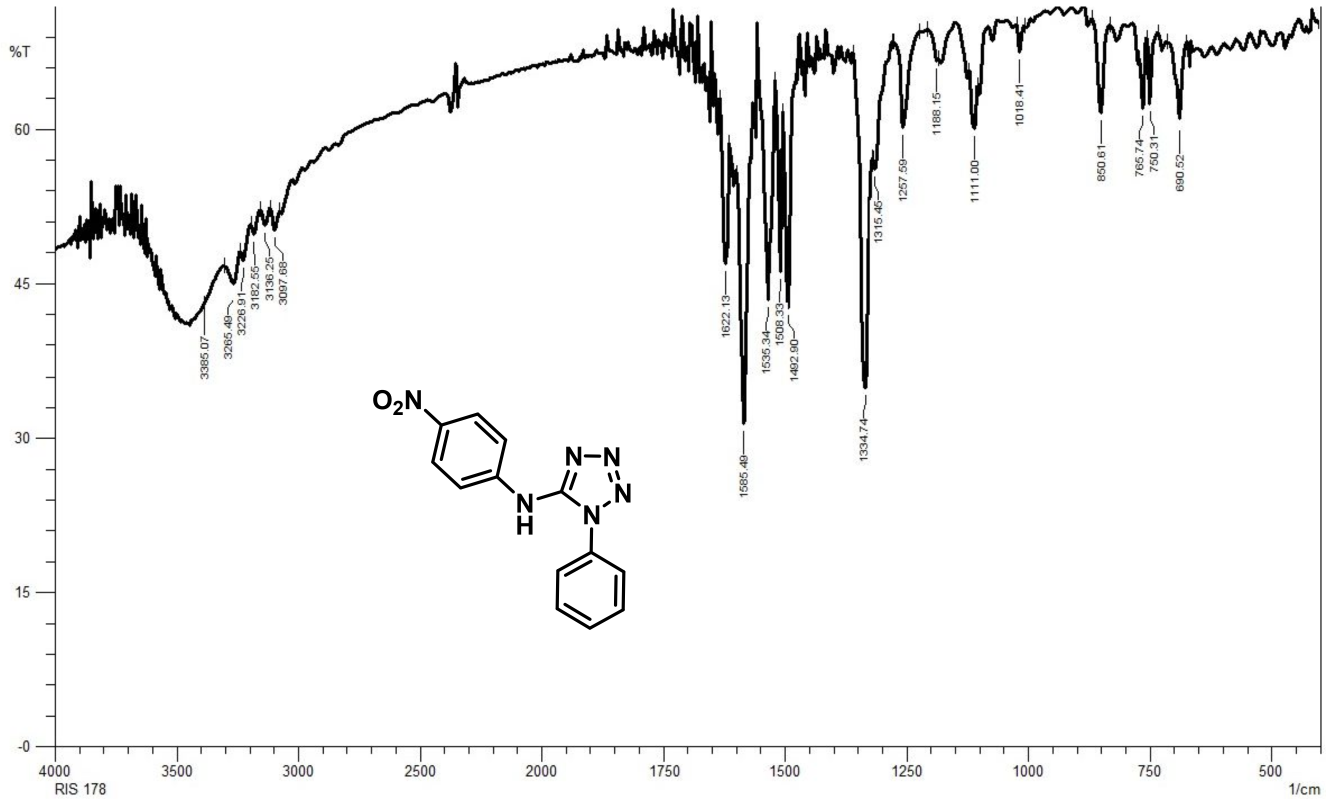

IR spectrum (KBr) of 3j'.

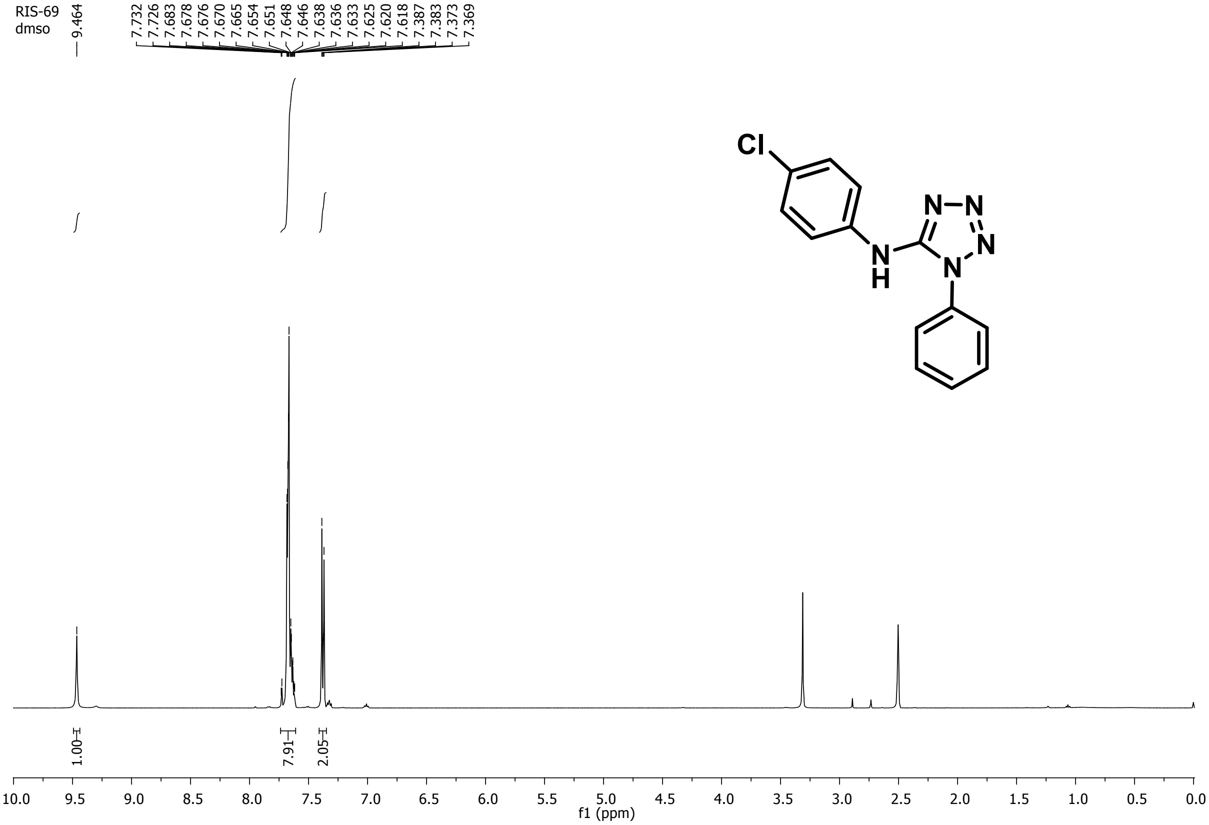

^1H NMR (DMSO-d_6, 500 MHz) of 3k'.

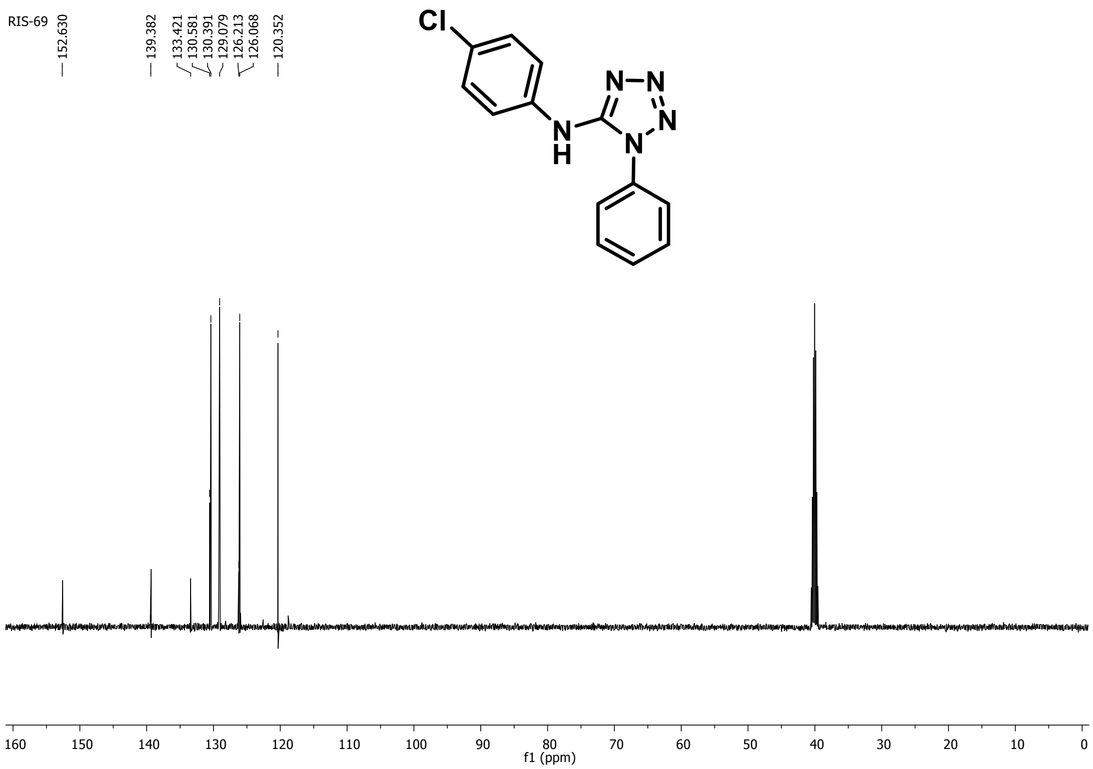

$^{13}C\{^1H\}$ NMR (DMSO-d_6, 125 MHz) of 3k'.

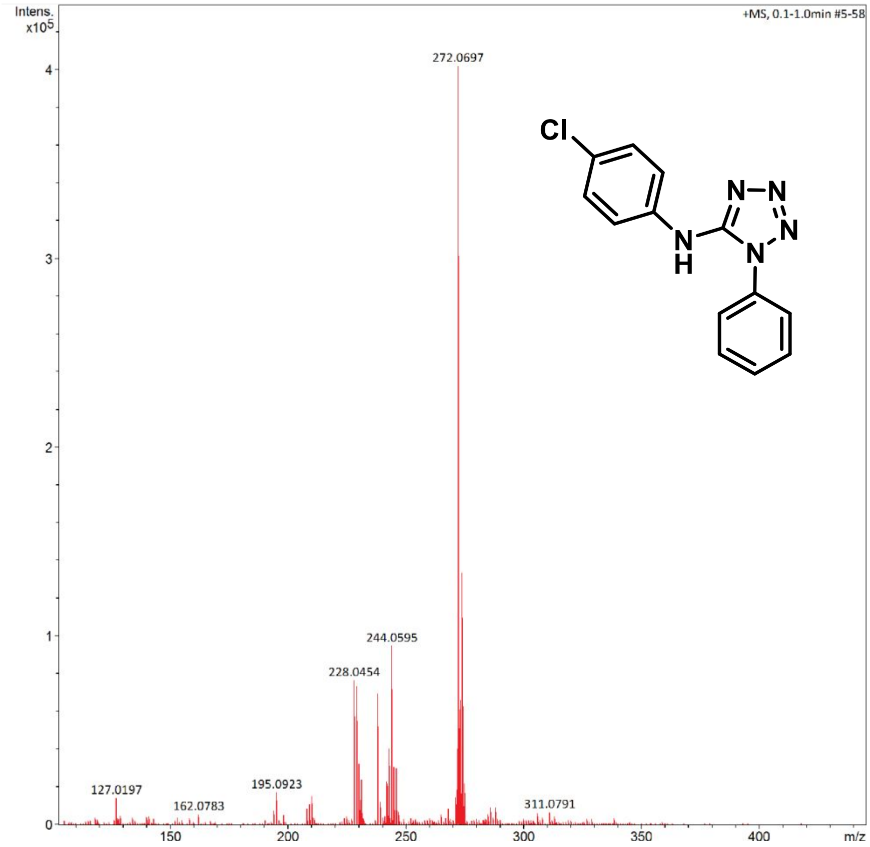

HRMS spectrum of 3k'.

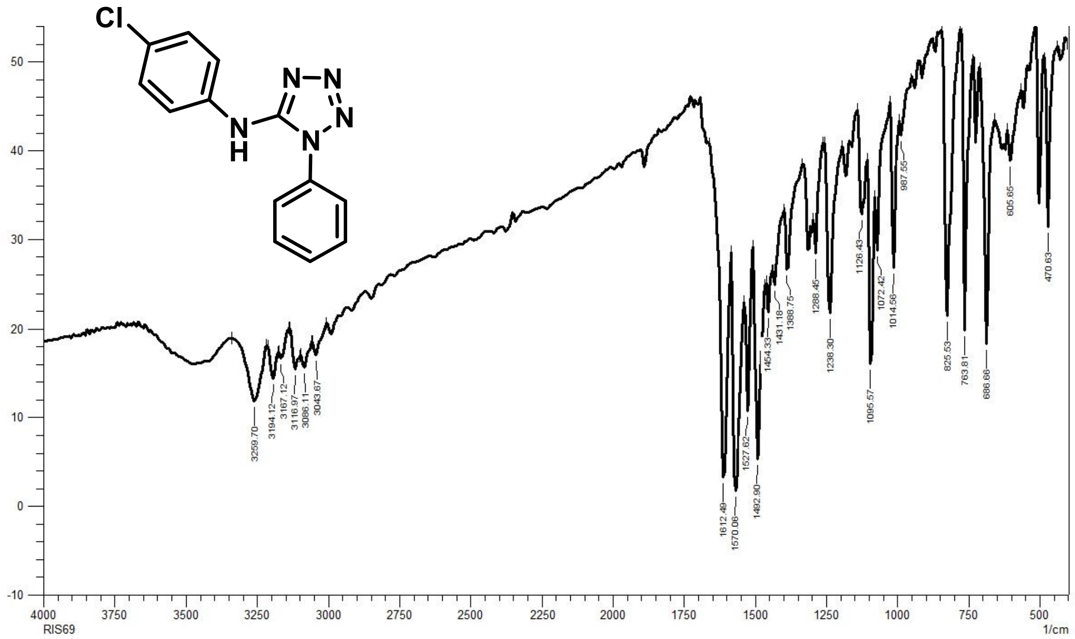

IR spectrum (KBr) of 3k'.

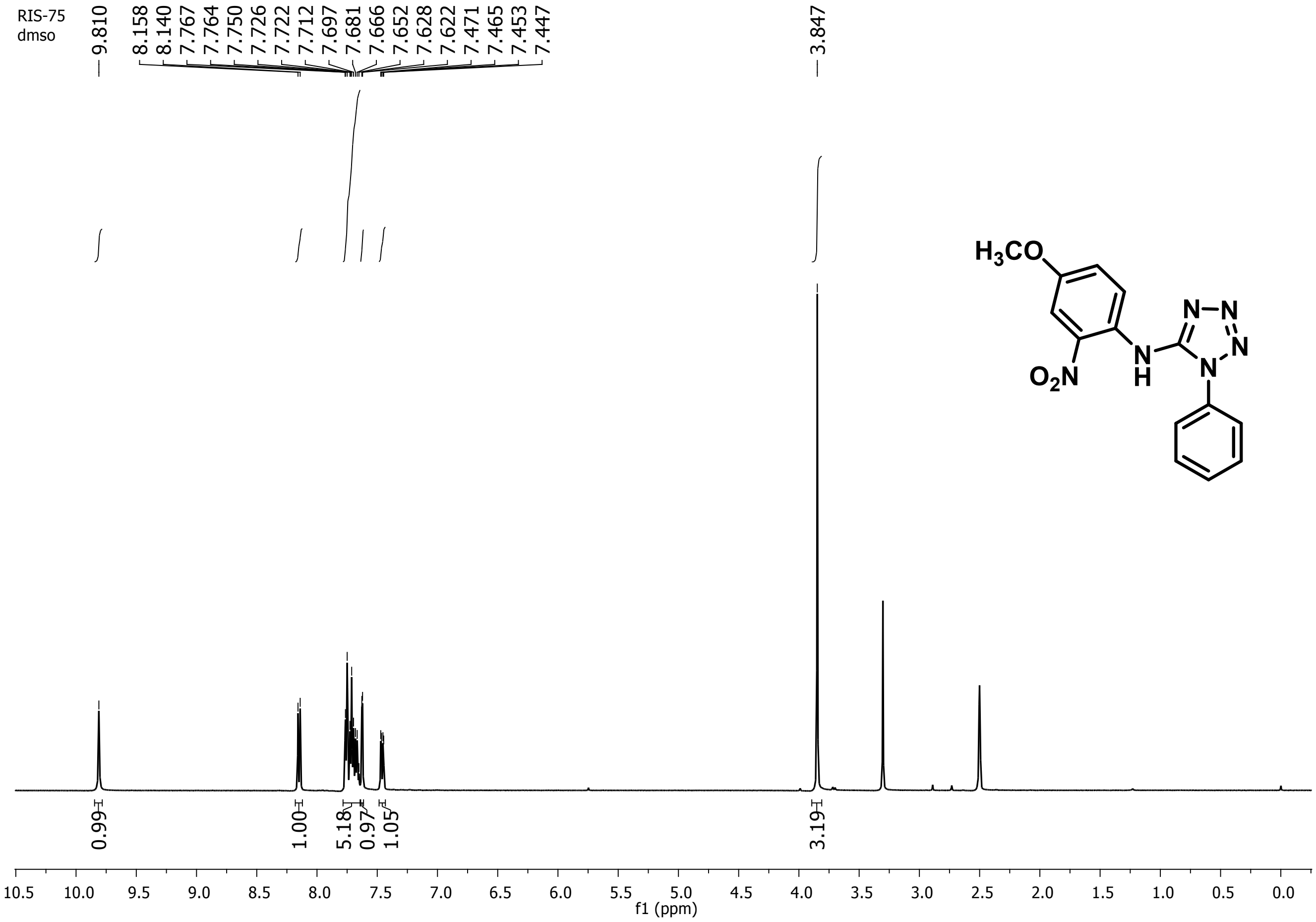

^{1}H NMR (DMSO-d_6, 500 MHz) of 3l'.

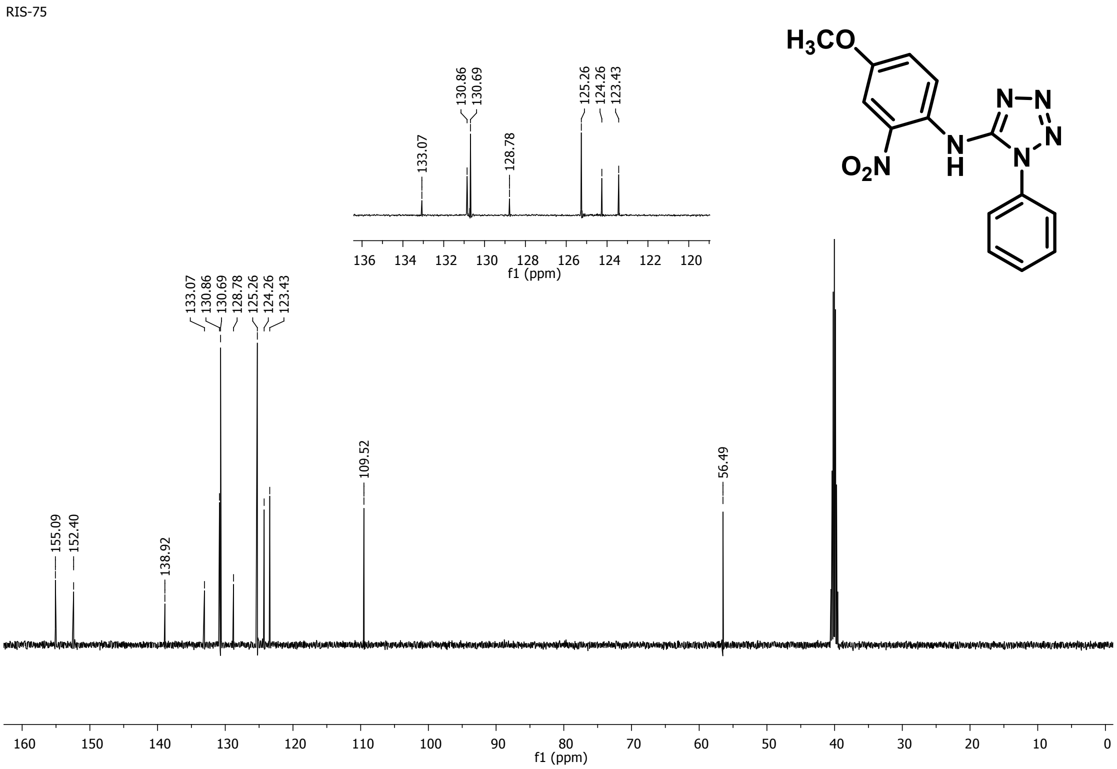

$^{13}C\{^1H\}$ *NMR (DMSO-d_6, 125 MHz) of 3l'.*

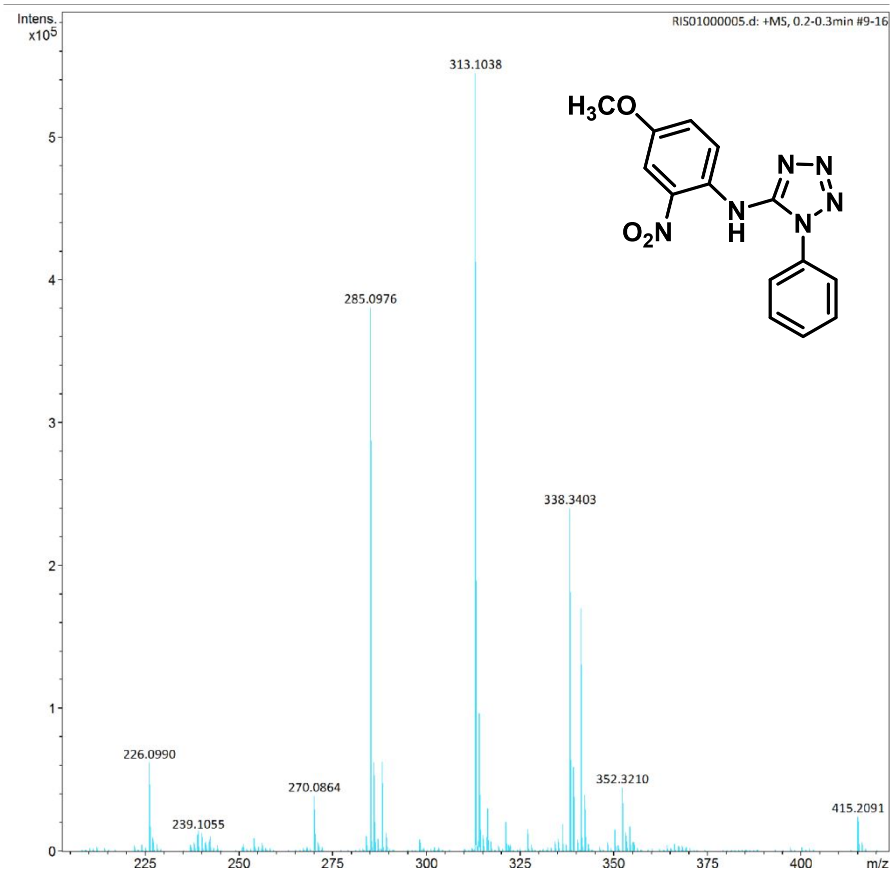

HRMS spectrum of 3l'.

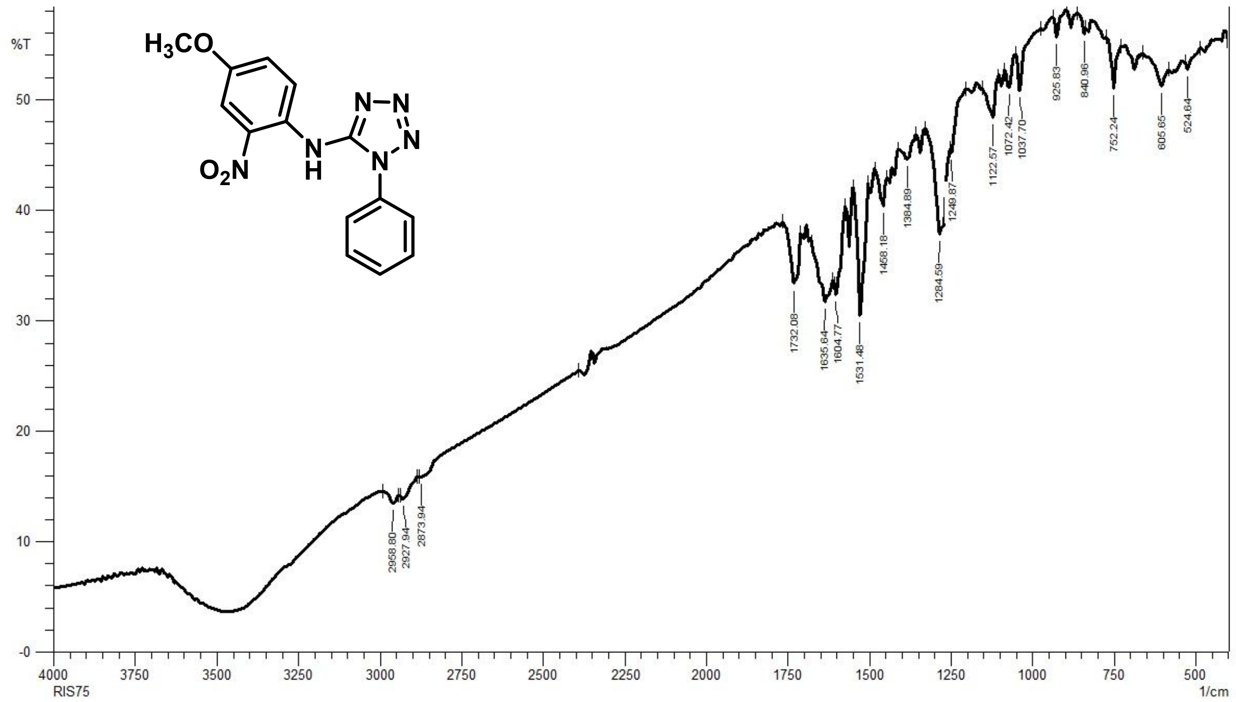

IR spectrum (KBr) of 3l'.

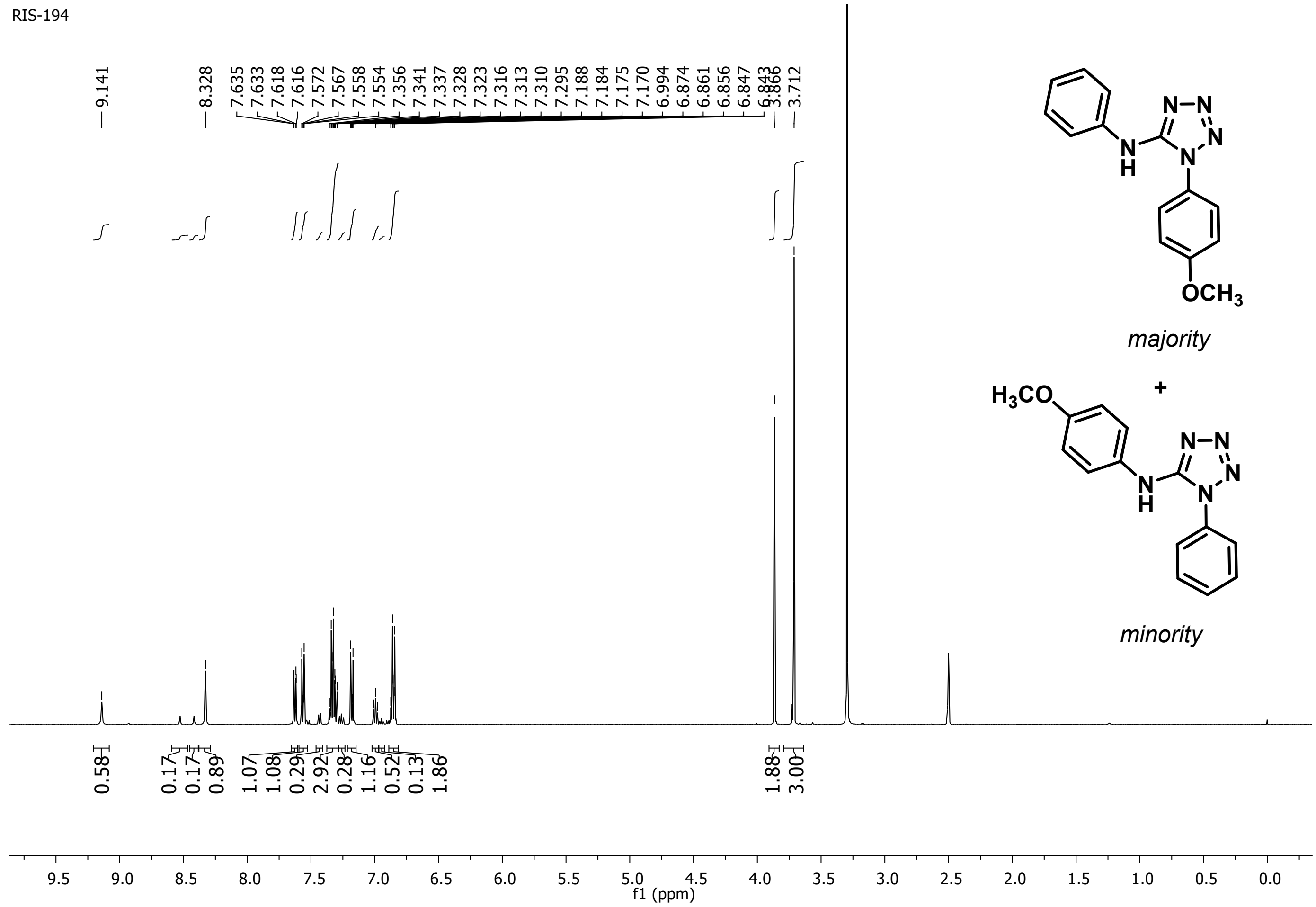

^1H NMR (DMSO-d_6, 500 MHz) of 3m and 3m' (from method C: 03 min).

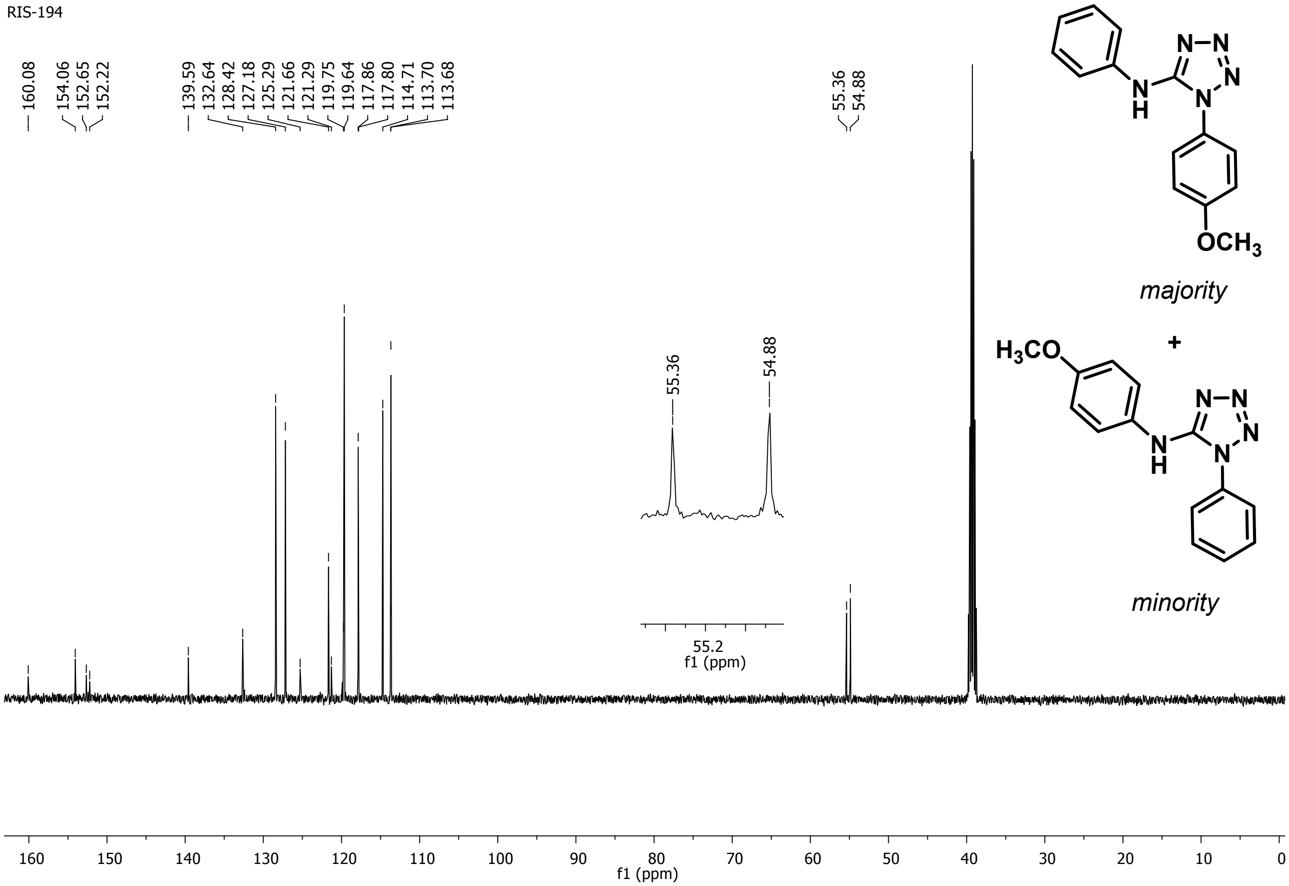

$^{13}C\{^{1}H\}$ NMR (DMSO-d_6, 125 MHz) of 3m and 3m' (from method C: 03 min).

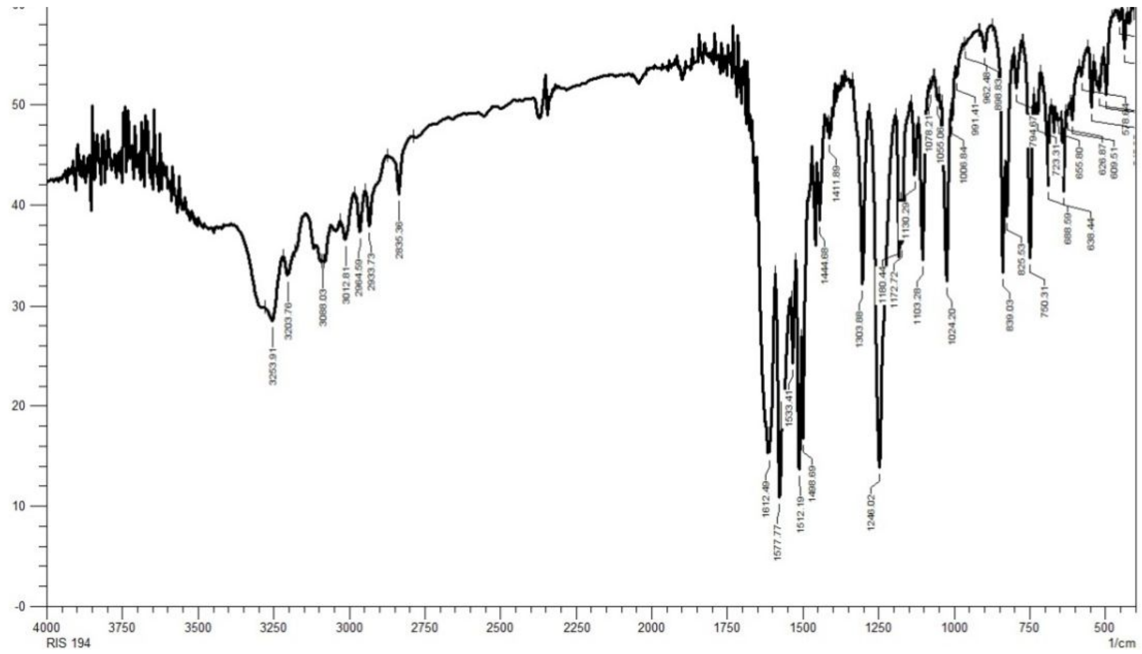

IR spectrum (KBr) of 3m and 3m' (from method C: 03 min).

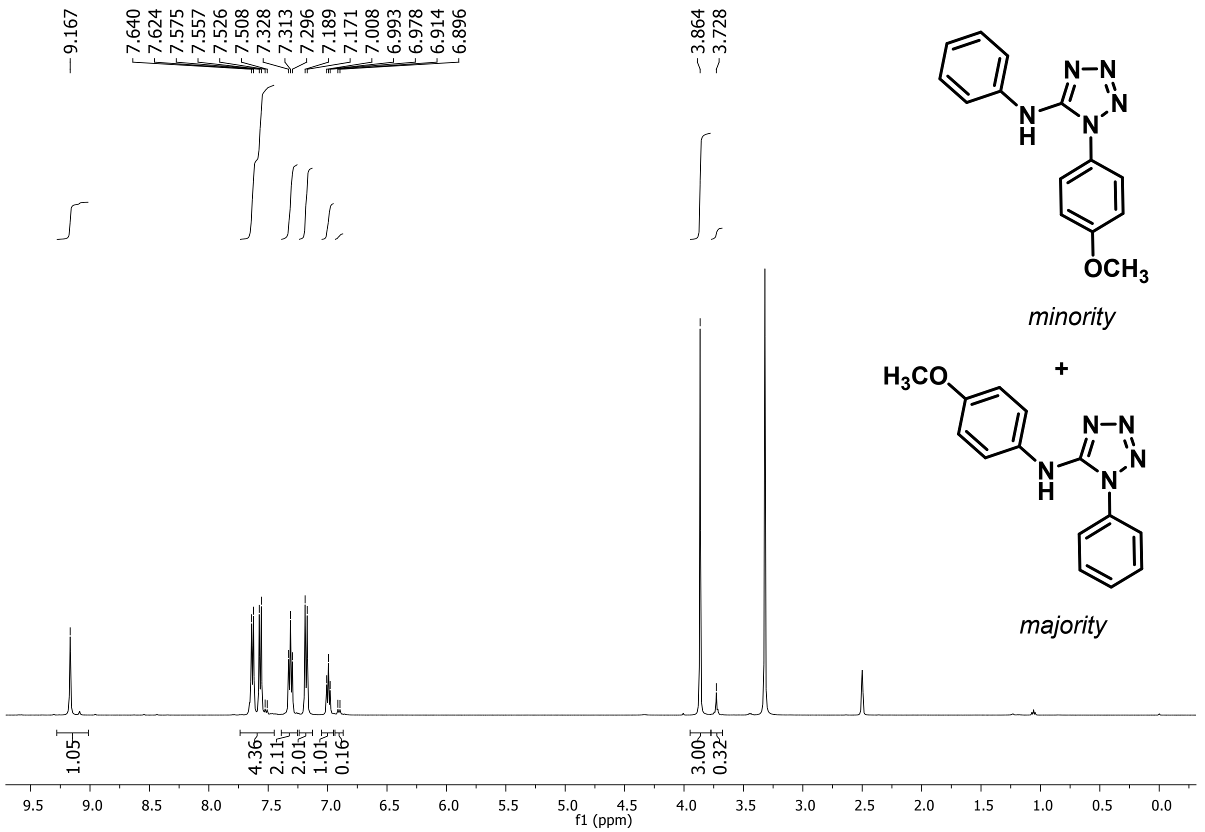

^{1}H NMR (DMSO-d_6, 500 MHz) of 3m and 3m' (from method A: 15 min).

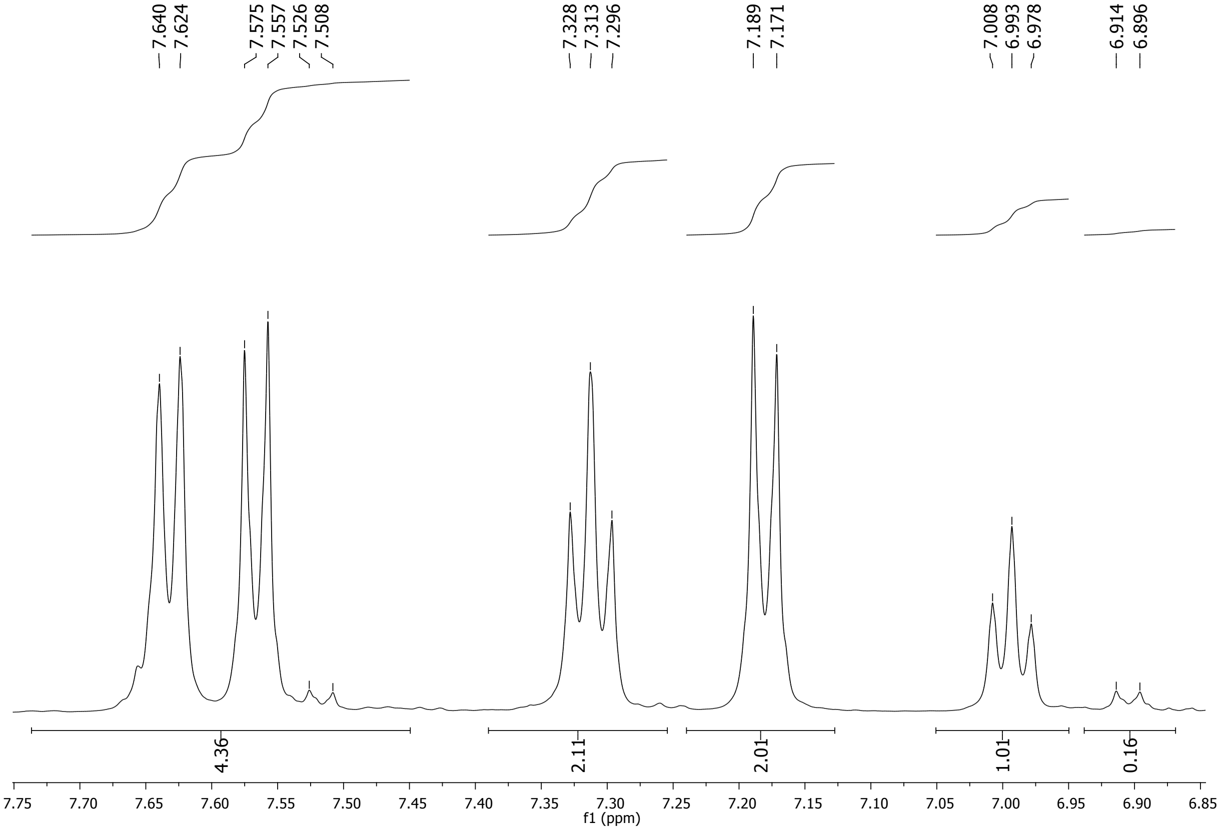

Expansion of ^{1}H NMR (DMSO-d_6, 500 MHz) of 3m and 3m' (from method A: 15 min).

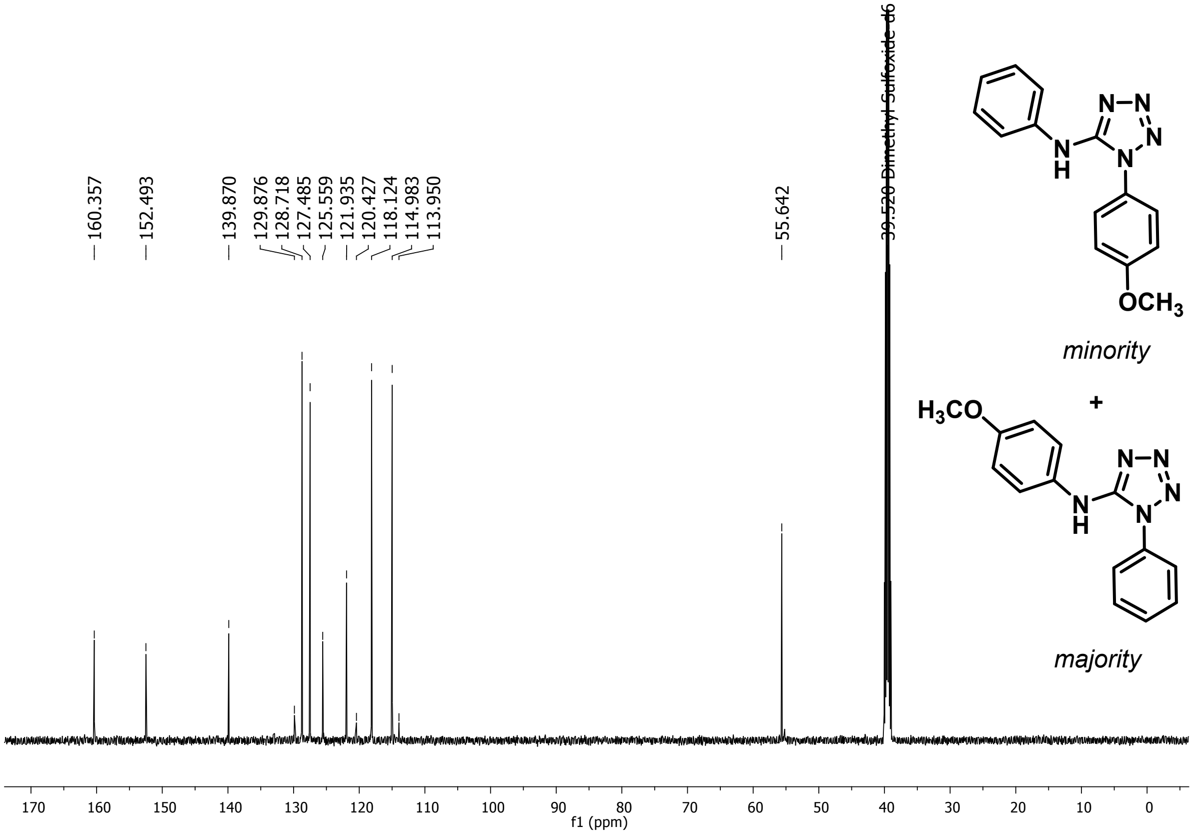

^{13}C{^{1}H} NMR (DMSO-d_6, 125 MHz) of 3m and 3m' (from method A: 15 min).

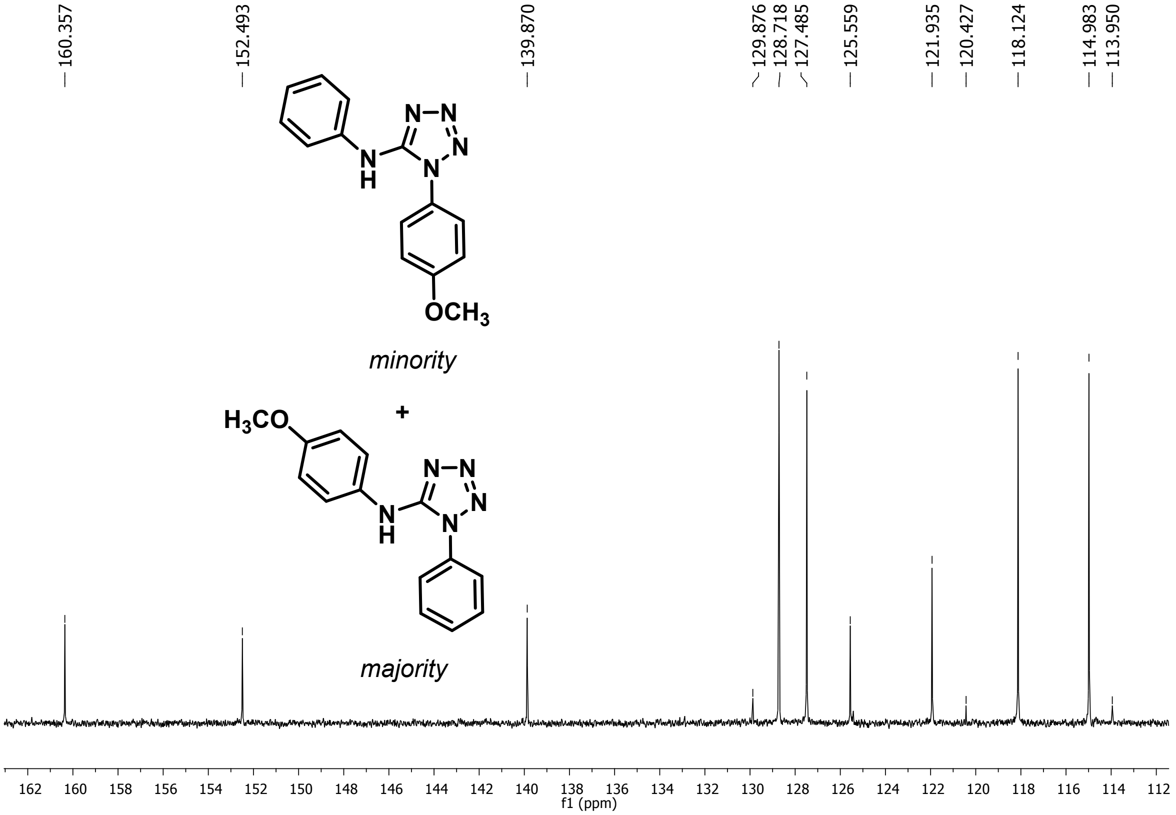

Expansion of ^{13}C{^{1}H} NMR (DMSO-d_6, 125 MHz) of 3m and 3m' (from method A: 15 min).

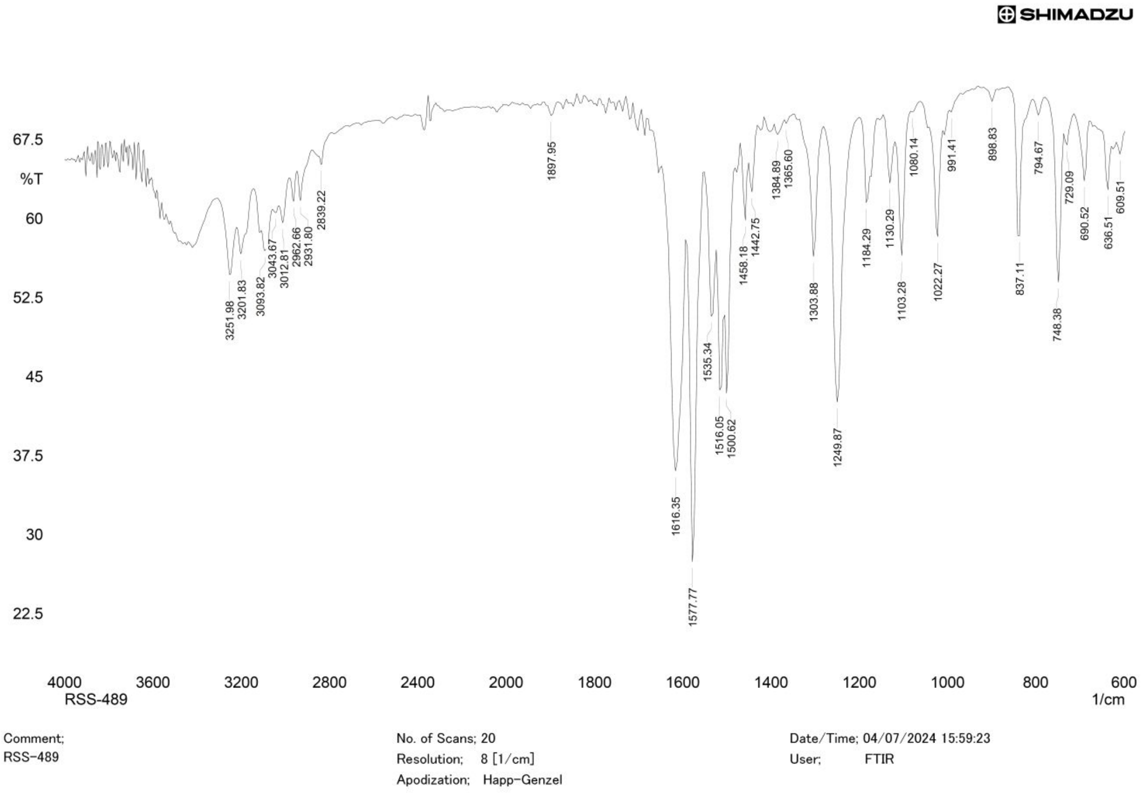

IR spectrum (KBr) of 3m and 3m' (from method A: 15 min).

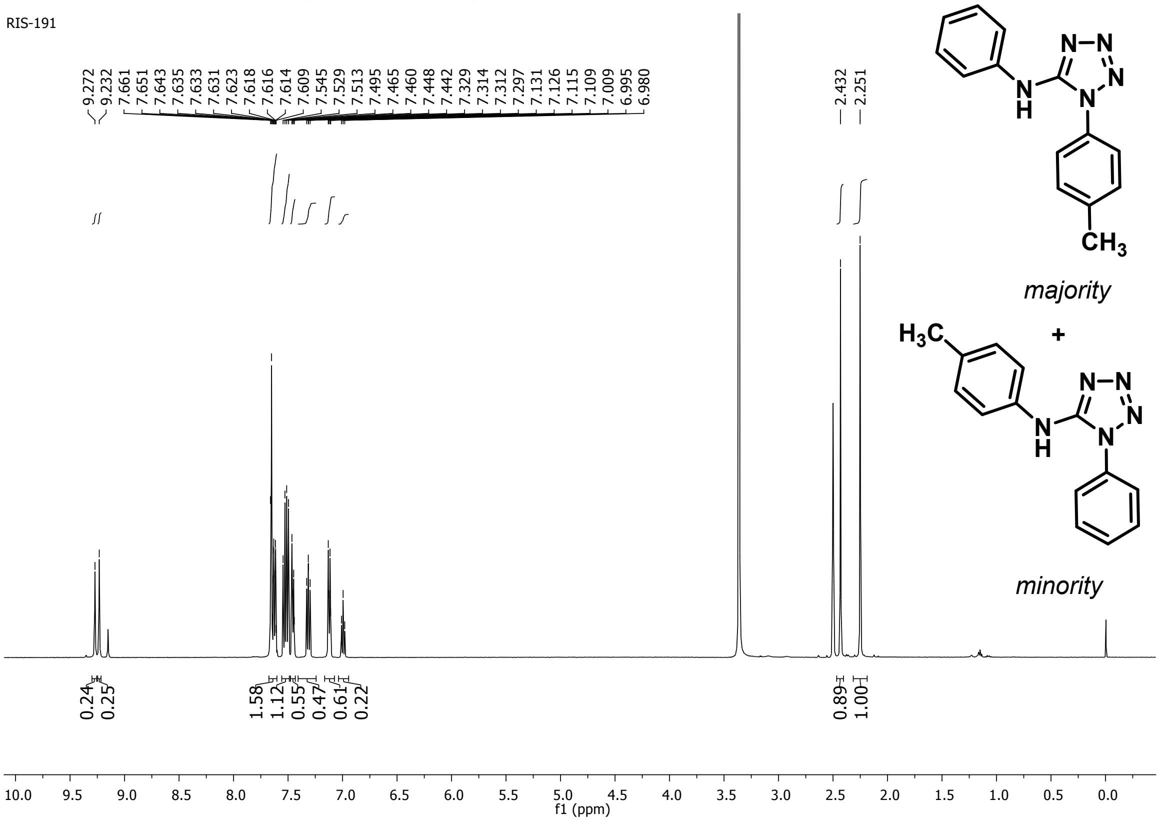

^{1}H NMR (DMSO-d_6, 500 MHz) of 3n and 3n' (from method C: 03 min).

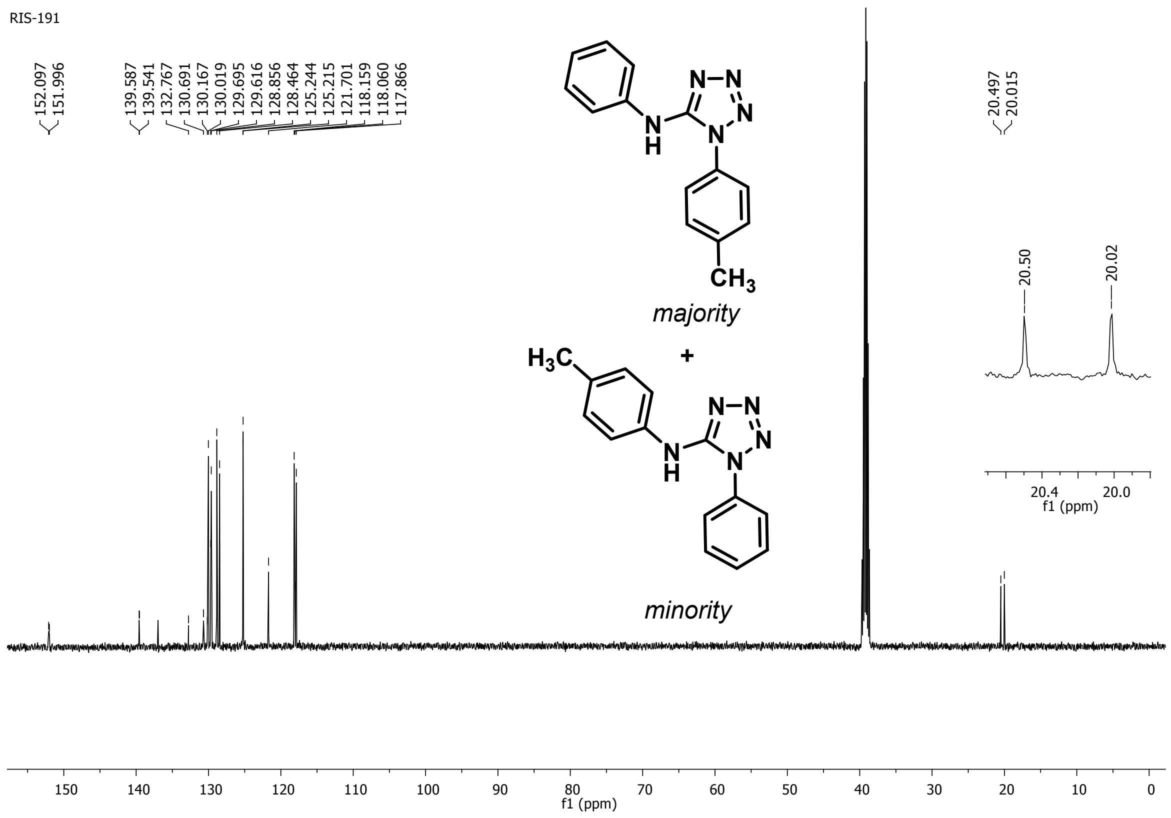

$^{13}C\{^1H\}$ NMR (DMSO-d_6, 125 MHz) of 3n and 3n' (from method C: 03 min).

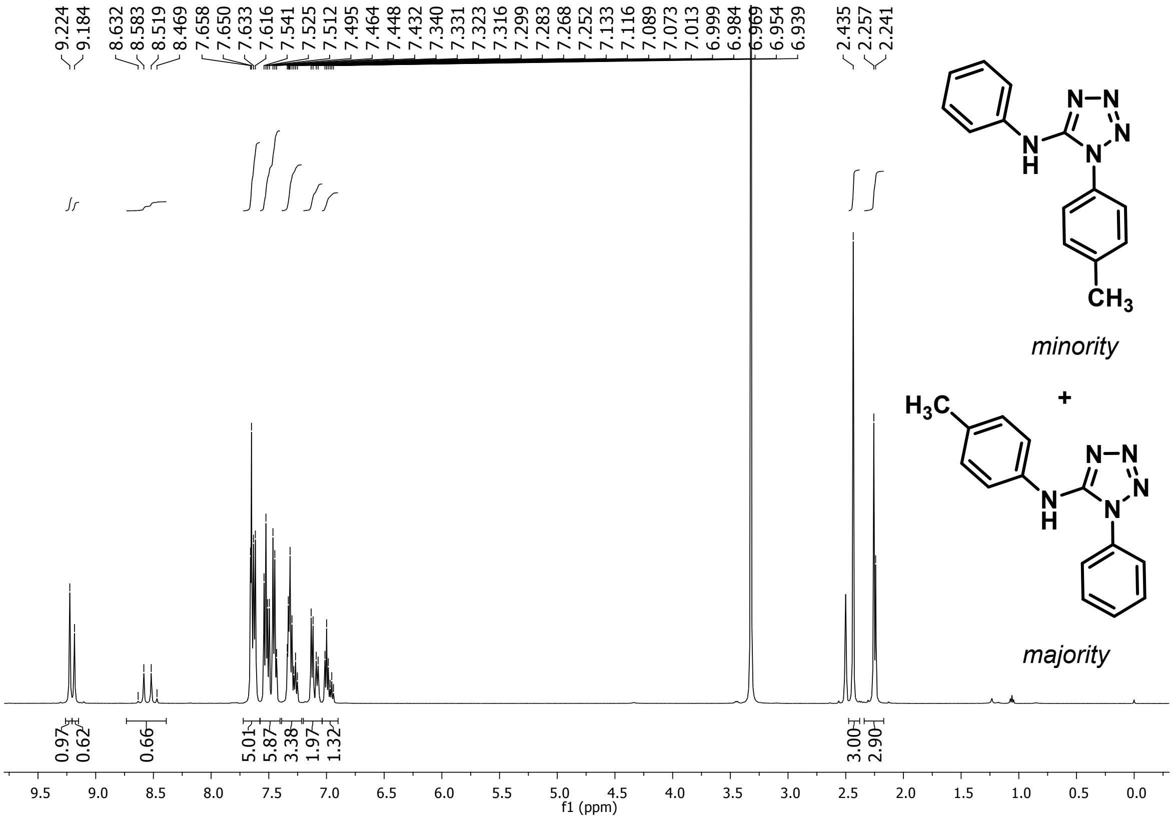

^1H NMR (DMSO-d_6, 500 MHz) of 3n, 3n' and 12 (from method A: 15 min).

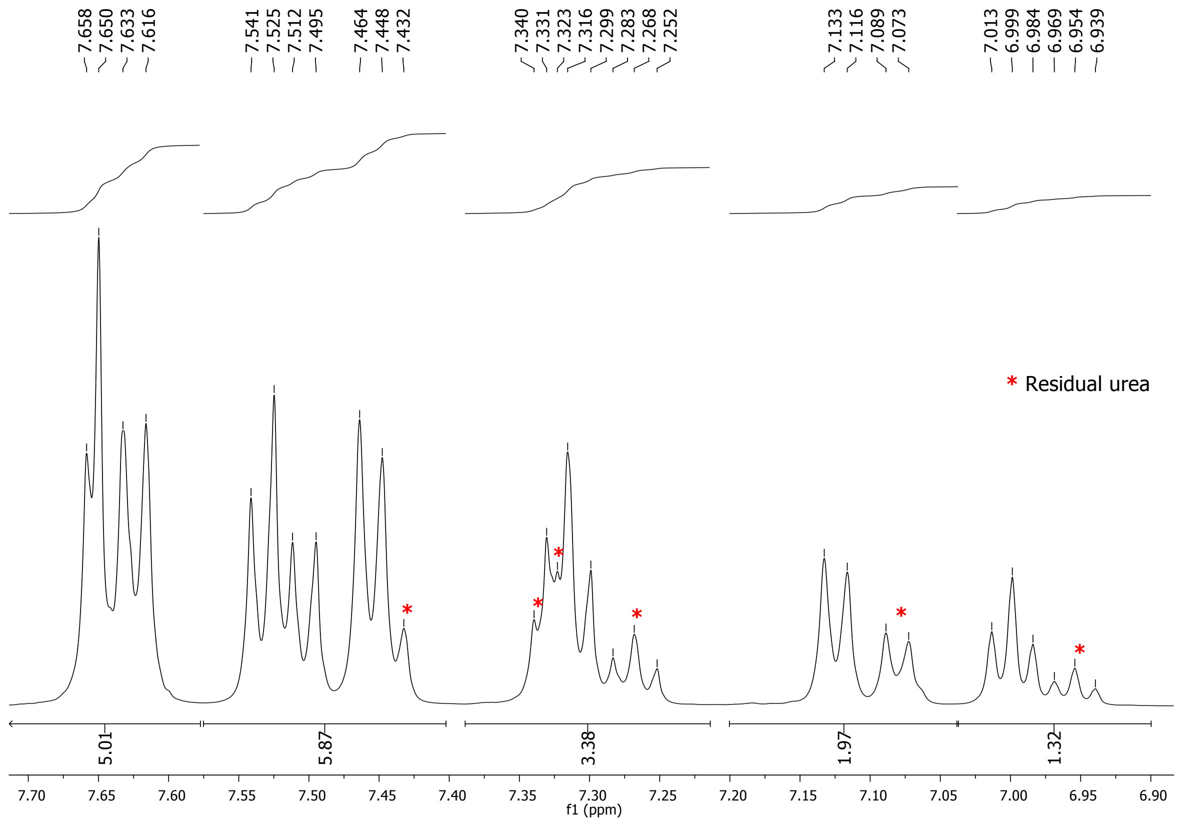

Expansion of ^{1}H NMR (DMSO-d_6, 500 MHz) of 3n, 3n' and 12 (from method A: 15 min).

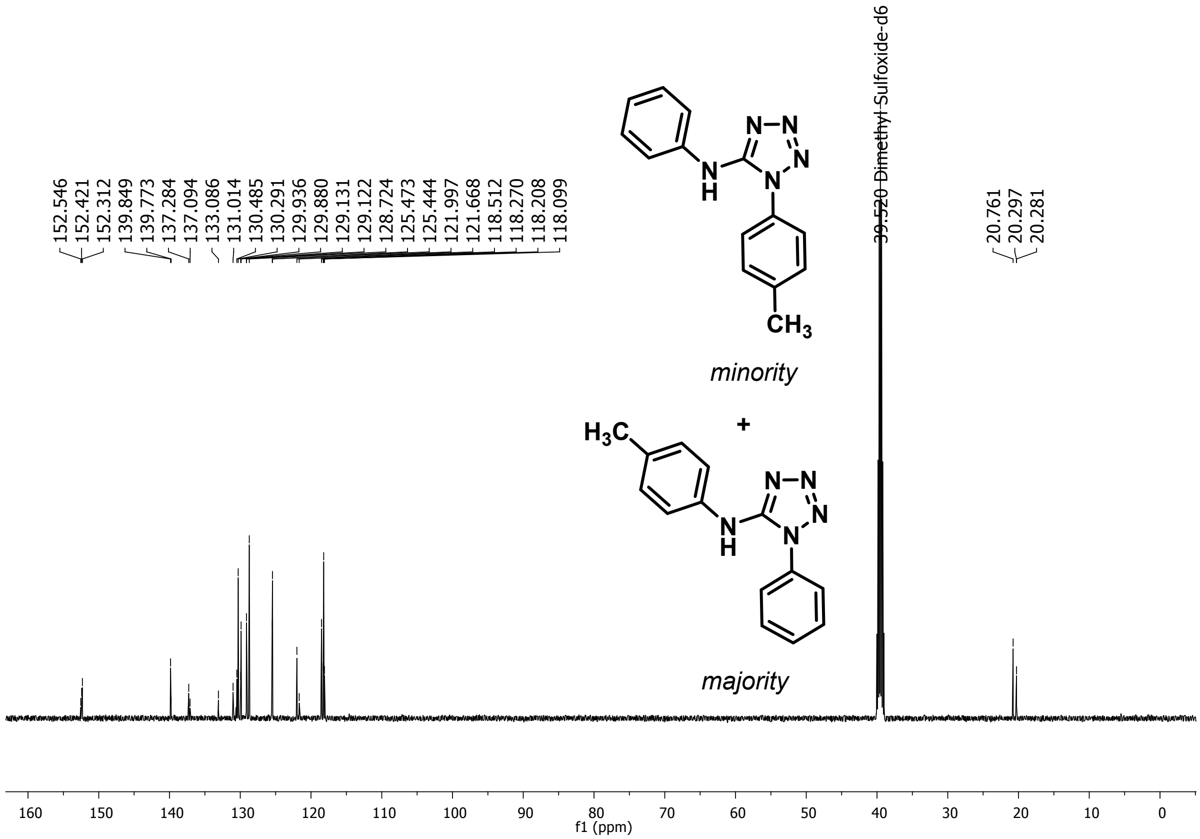

$^{13}C\{^{1}H\}$ NMR (DMSO-d_6, 125 MHz) of 3n, 3n' and 12 (from method A: 15 min).

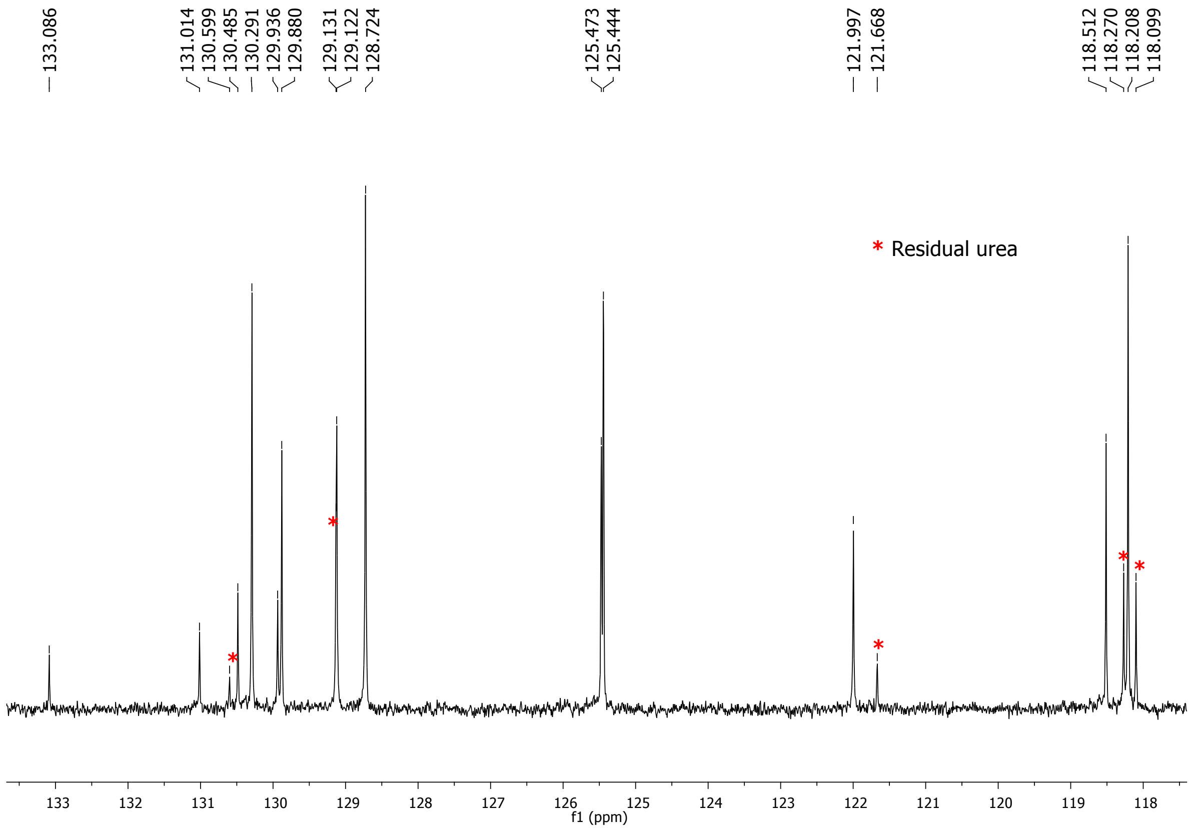

Expansion $^{13}C\{^1H\}$ NMR (DMSO-d_6, 125 MHz) of 3n, 3n' and 12 (from method A: 15 min).

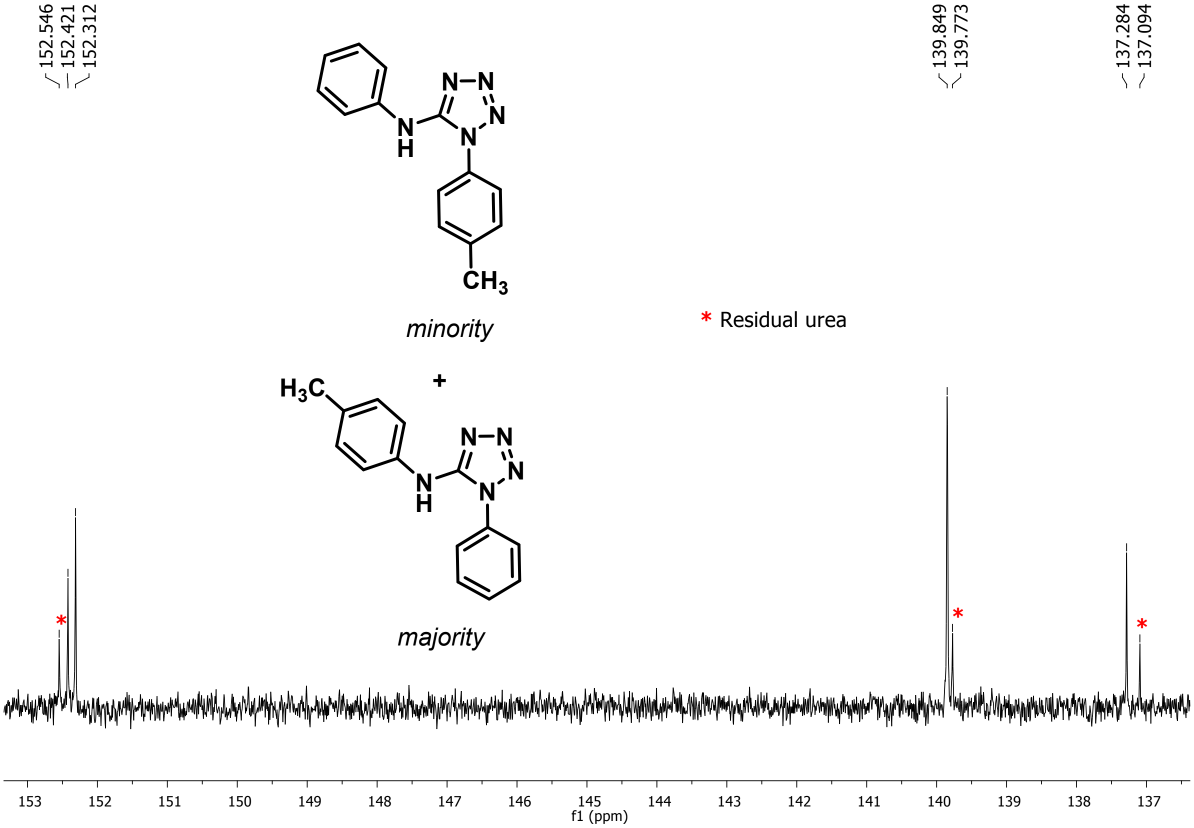

Expansion $^{13}C\{^1H\}$ NMR (DMSO-d_6, 125 MHz) of 3n, 3n' and 12 (from method A: 15 min).

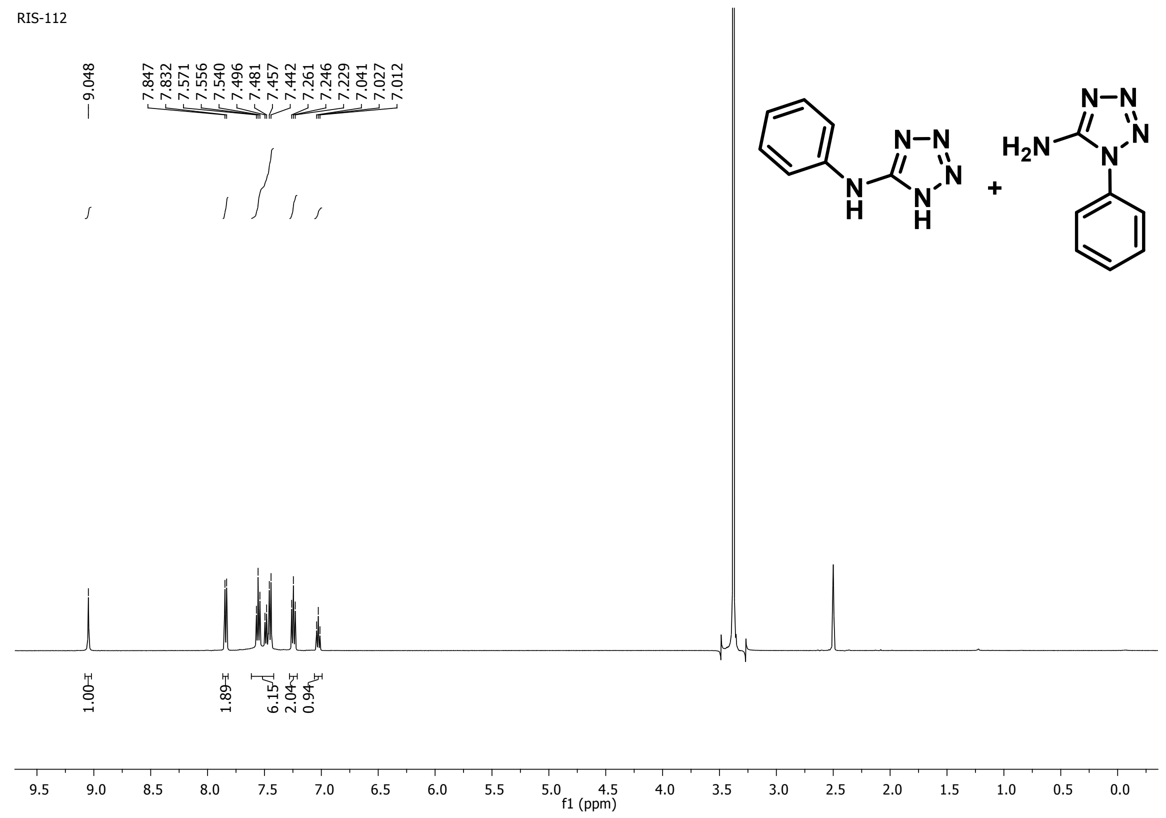

^{1}H NMR (DMSO-d$_6$, 500 MHz) of 3o and 3o'.

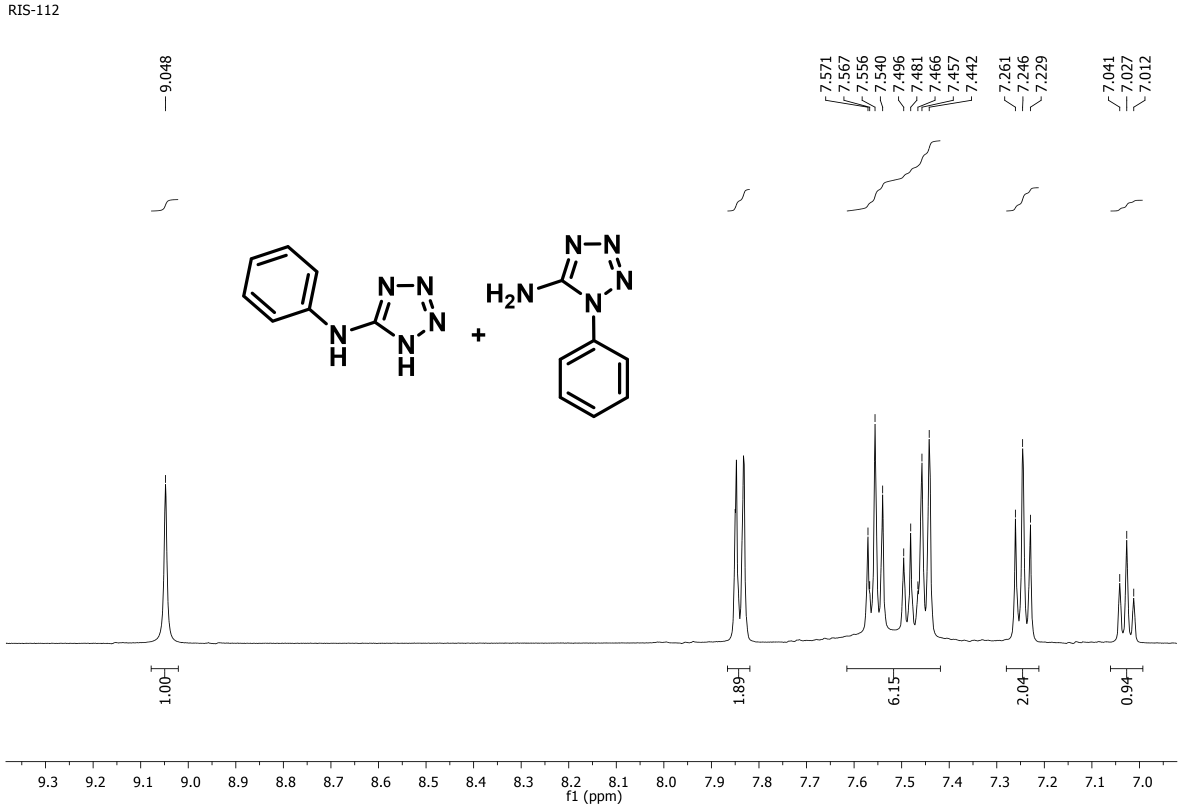

^{1}H NMR (DMSO-d$_6$, 500 MHz) of 3o and 3o'.

RIS-112

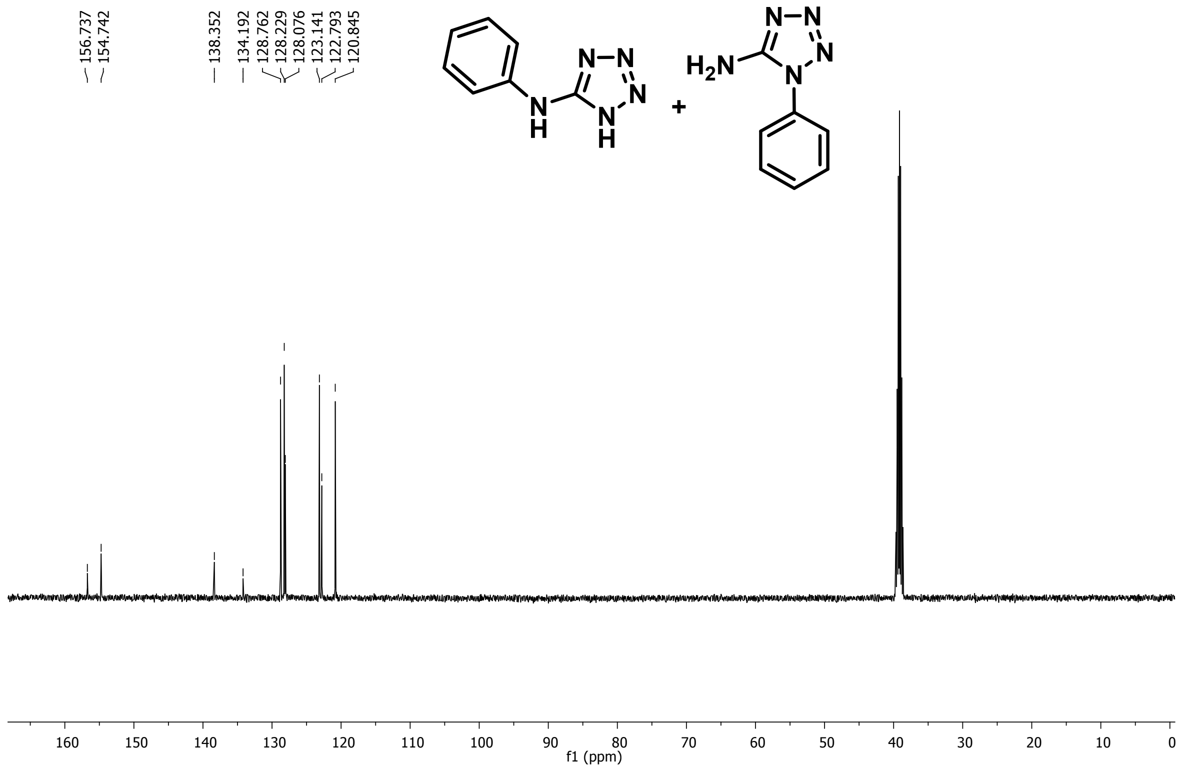

$^{13}C\{^{1}H\}$ NMR (DMSO-d_6, 125 MHz) of 3o and 3o'.

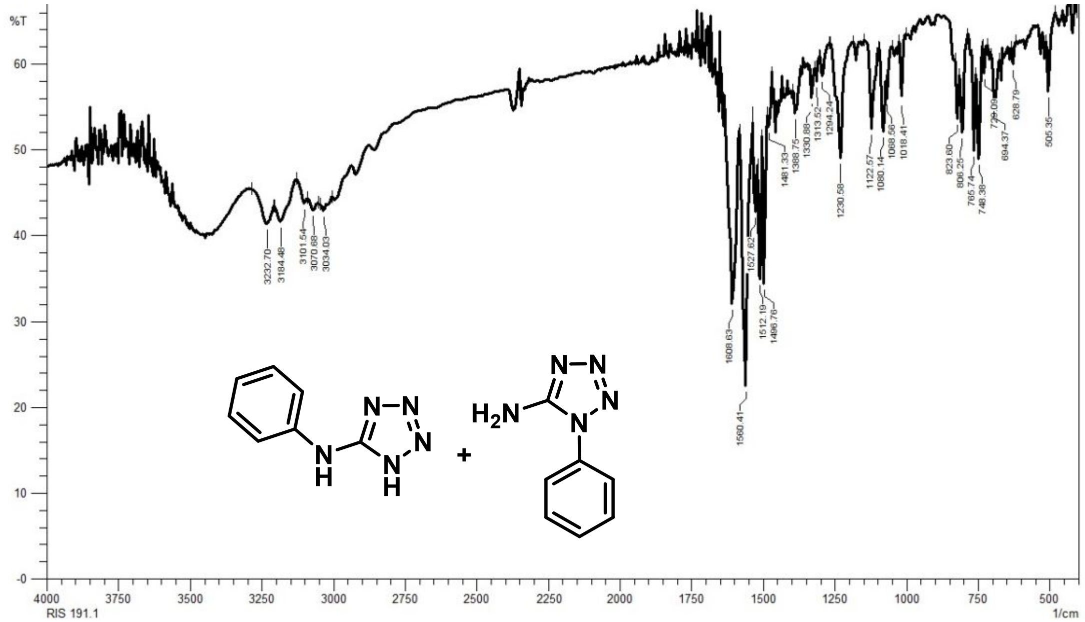

IR spectrum (KBr) of 3o and 3o'.

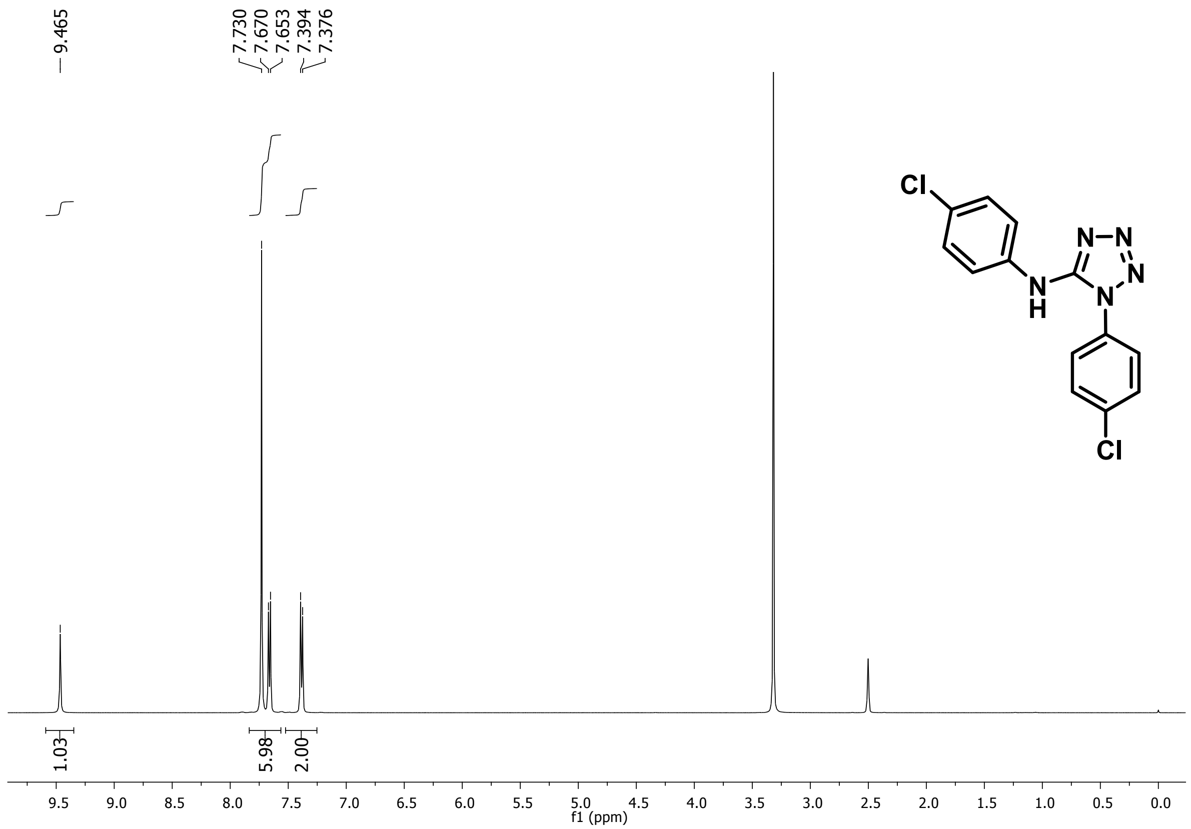

^{1}H NMR (DMSO-d_6, 500 MHz) of 3p.

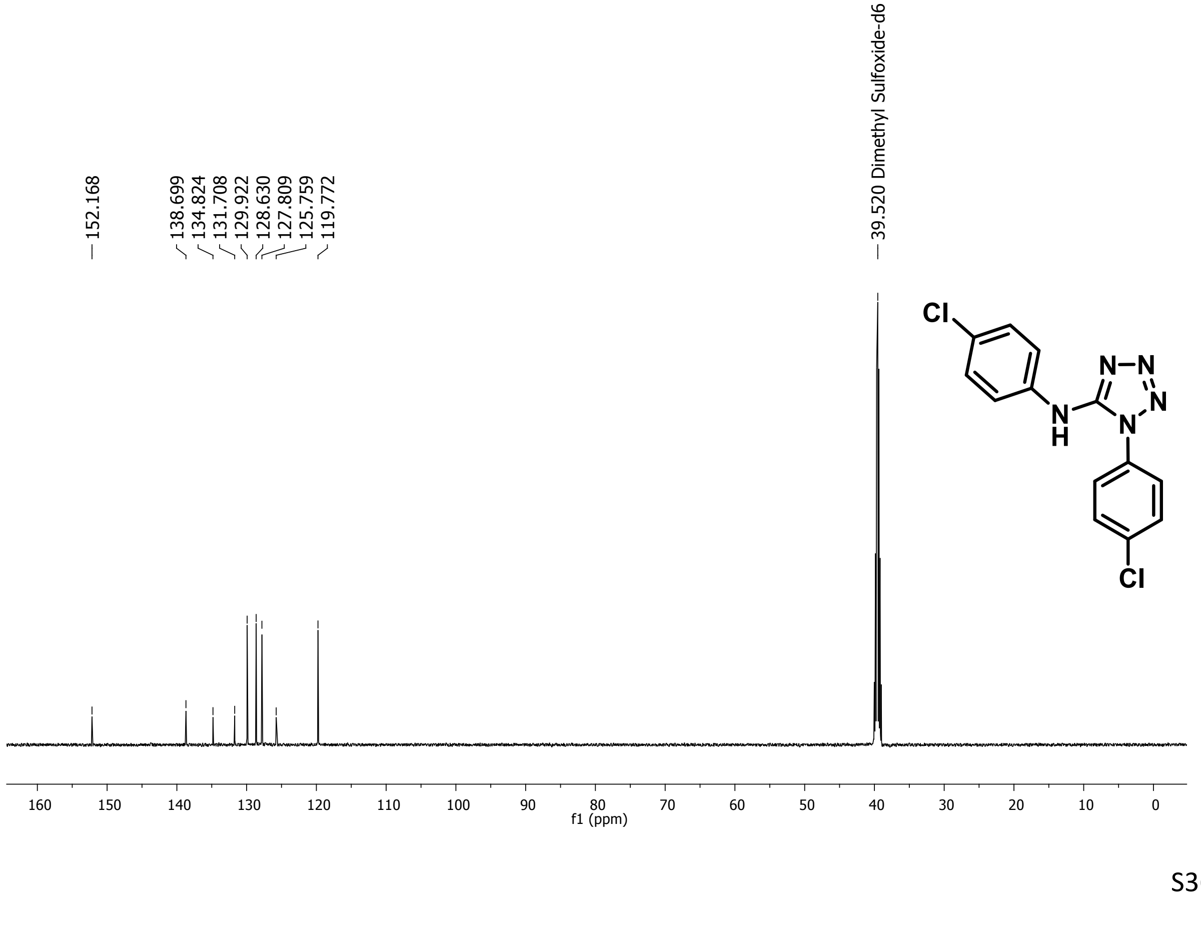

$^{13}C\{^1H\}$ NMR (DMSO-d_6, 125 MHz) of 3p.

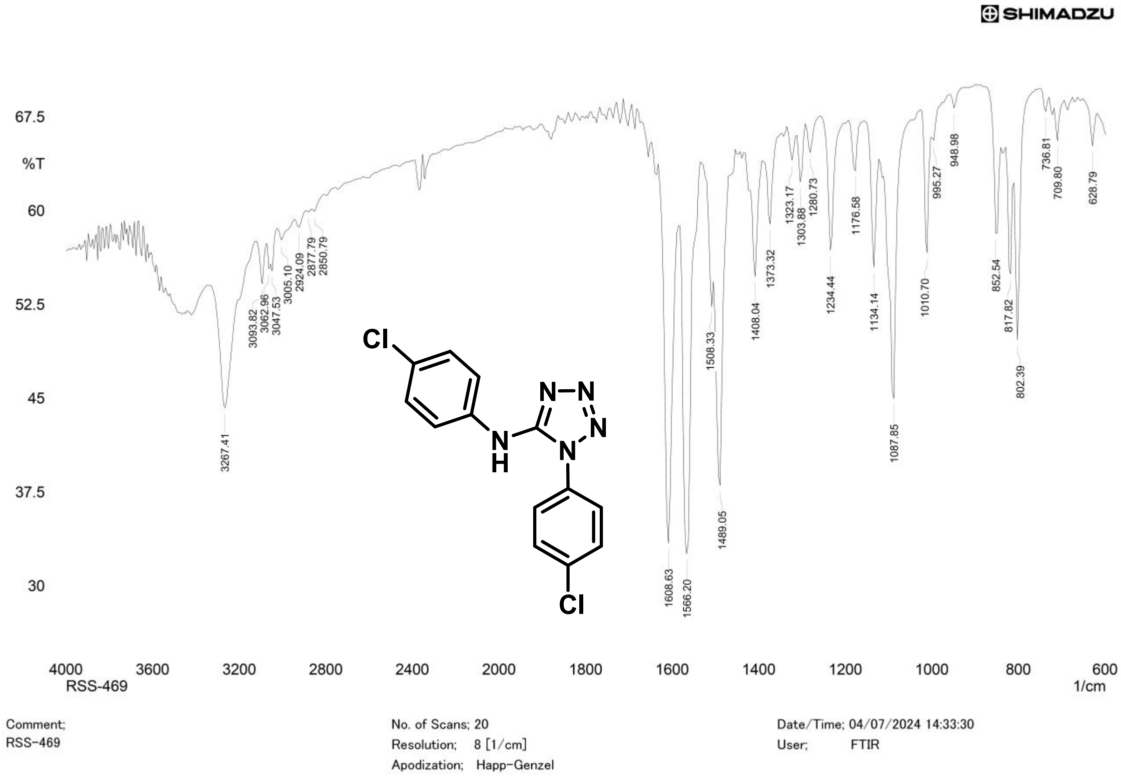

IR spectrum (KBr) of 3p.

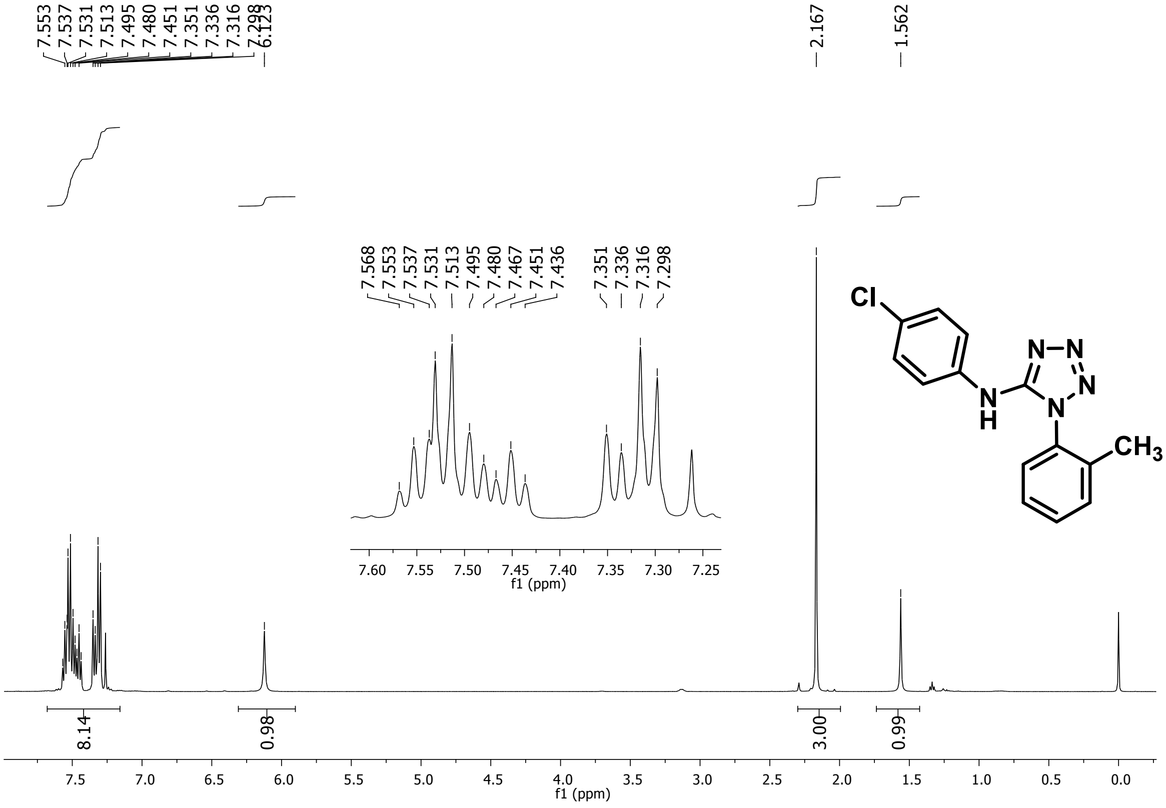

^{1}H NMR ($CDCl_3$, 500 MHz) of 3q.

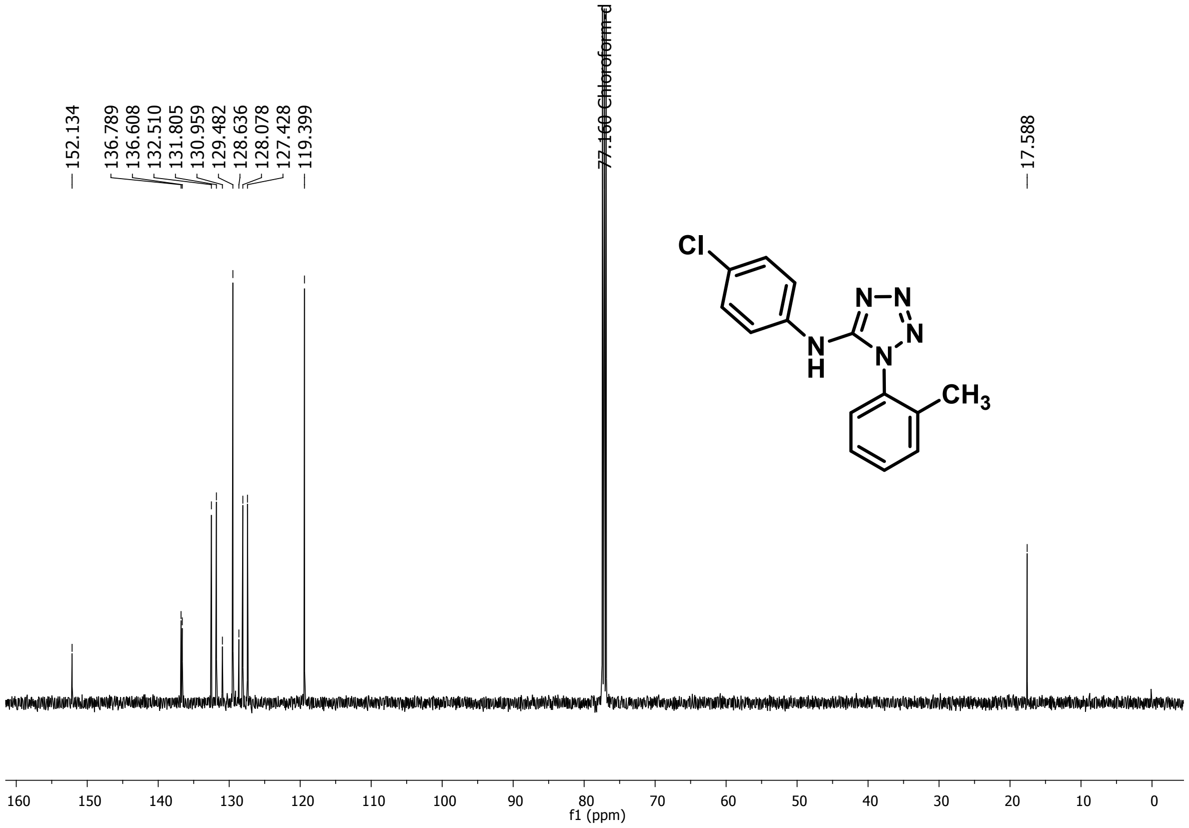

$^{13}C\{^{1}H\}$ NMR ($CDCl_3$, 125 MHz) of 3q.

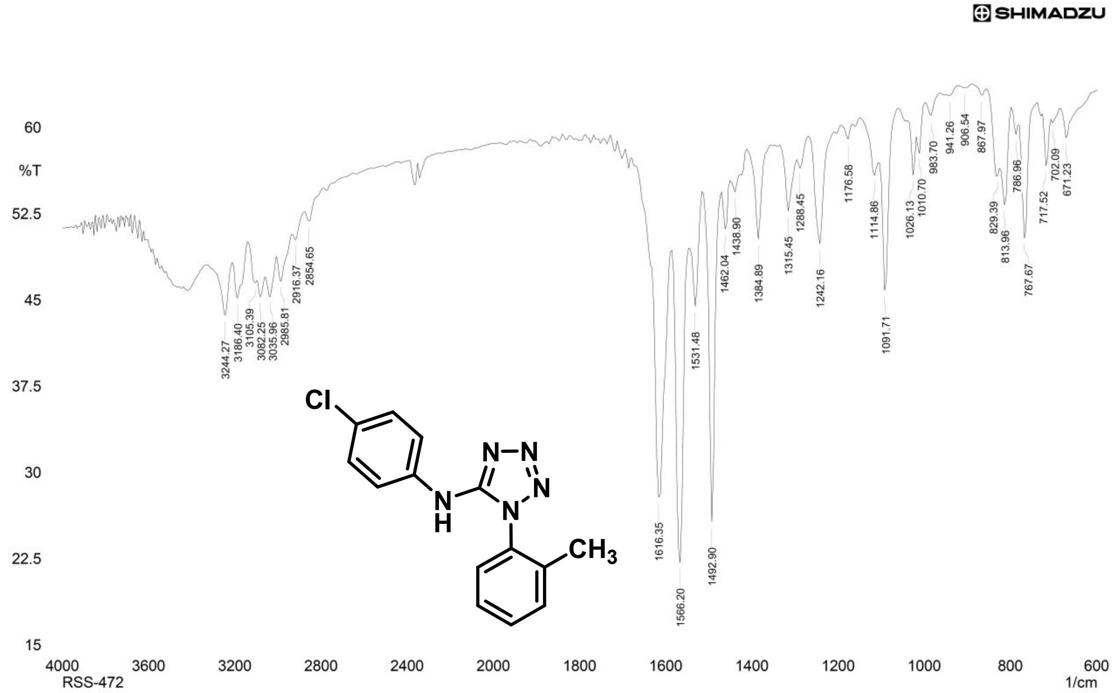

Comment;
RSS-472

No. of Scans; 20
Resolution; 8 [1/cm]
Apodization; Happ-Genzel

Date/Time; 04/07/2024 14:49:22
User; FTIR

IR spectrum (KBr) of 3q.

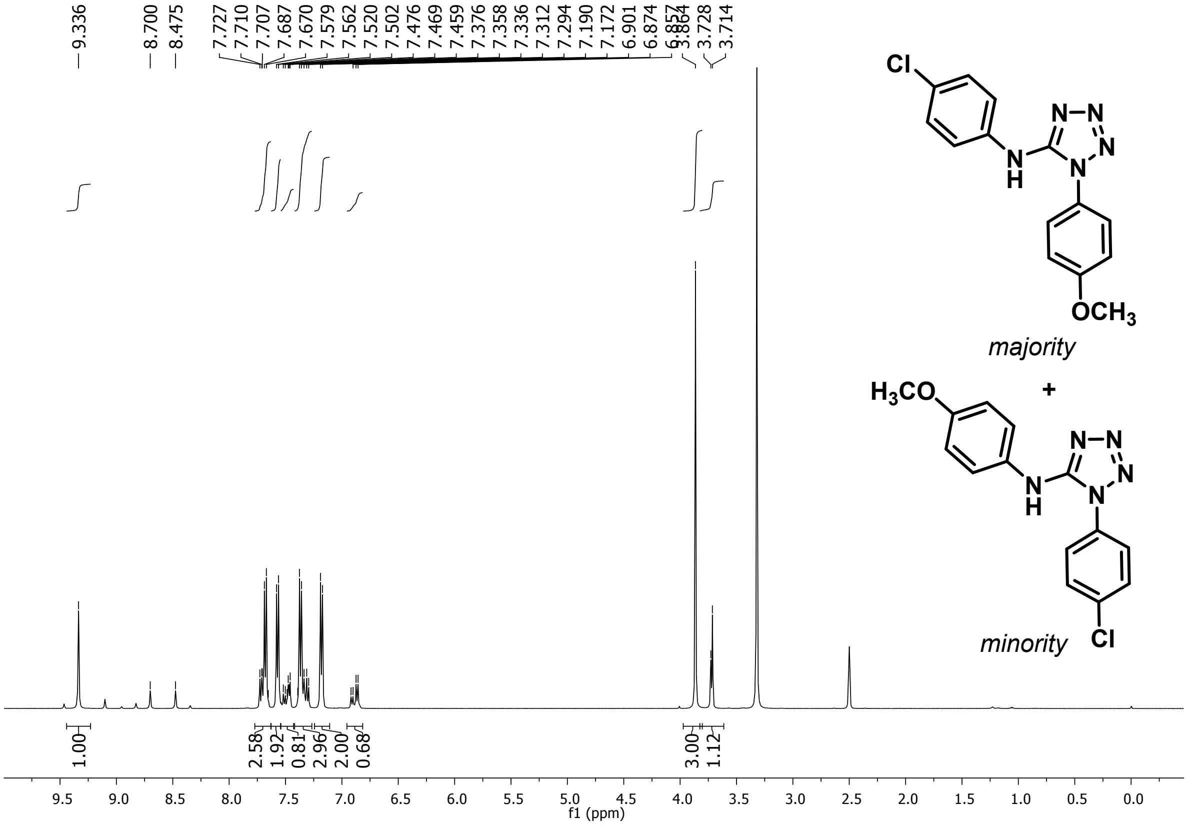

^{1}H NMR (DMSO-d_6, 500 MHz) of 3r, 3r' and 13.

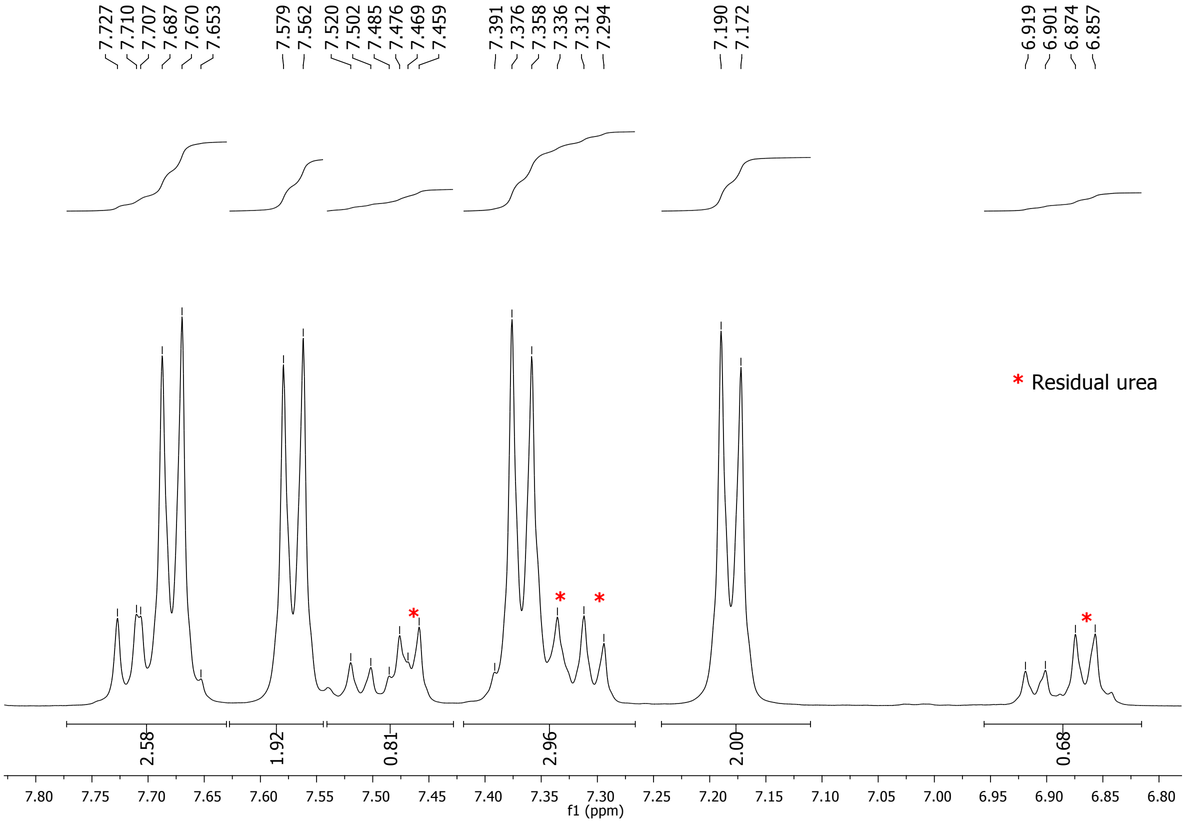

Expansion of ^{1}H NMR (DMSO-d_6, 500 MHz) of 3r, 3r' and 13.

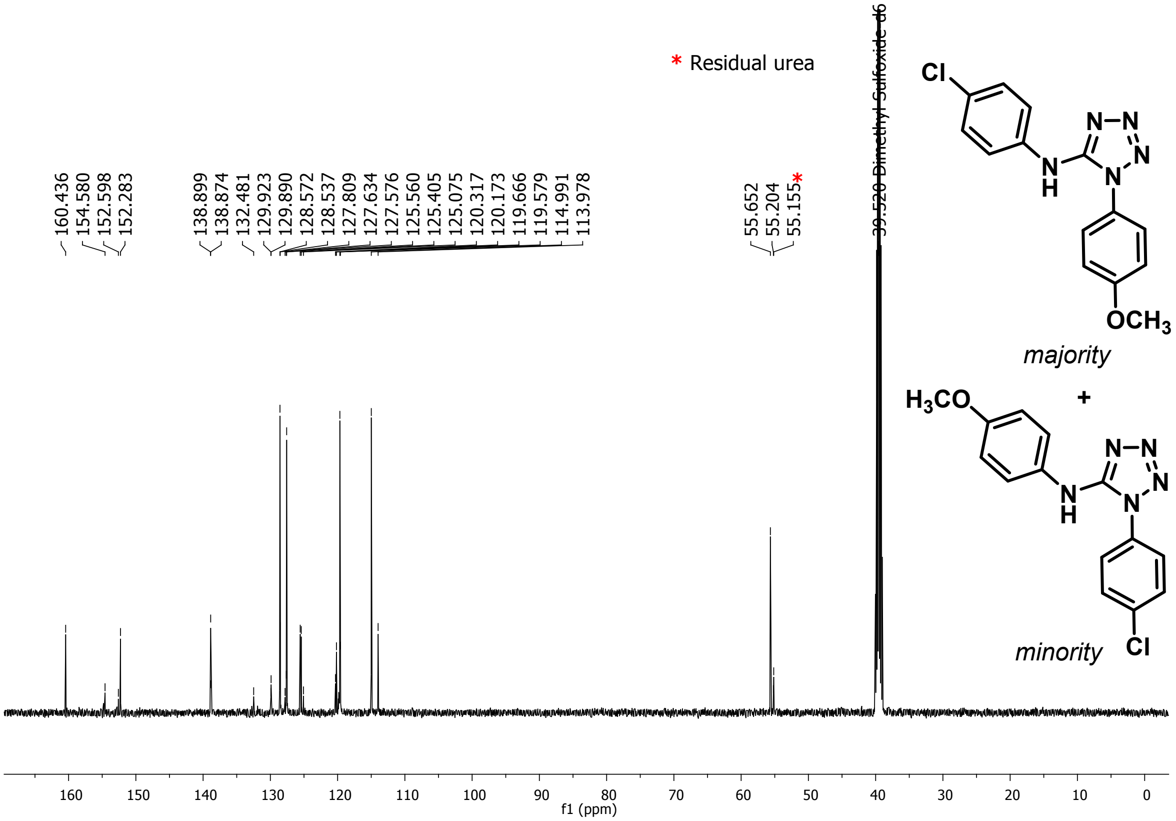

$^{13}C\{^{1}H\}$ NMR (DMSO-d_6, 125 MHz) of 3r, 3r' and 13.

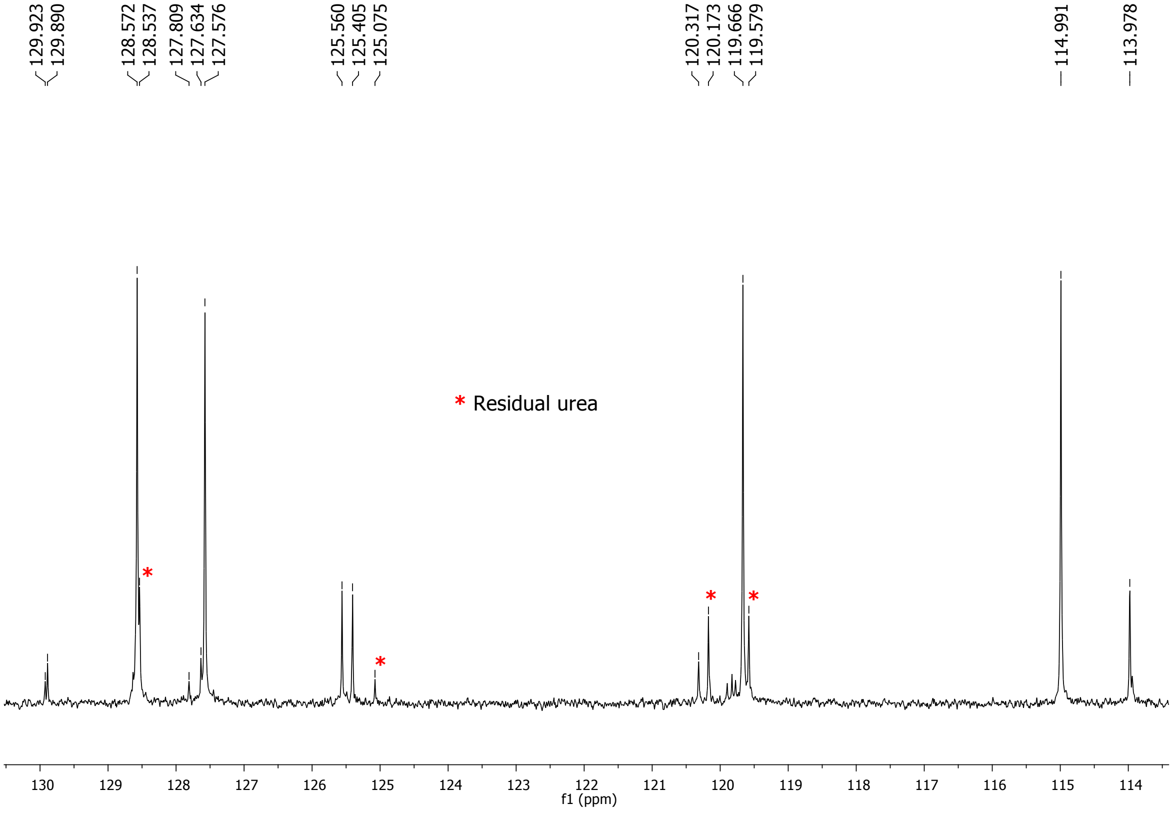

Expansion of $^{13}C\{^{1}H\}$ NMR (DMSO-d_6, 125 MHz) of 3r, 3r' and 13.

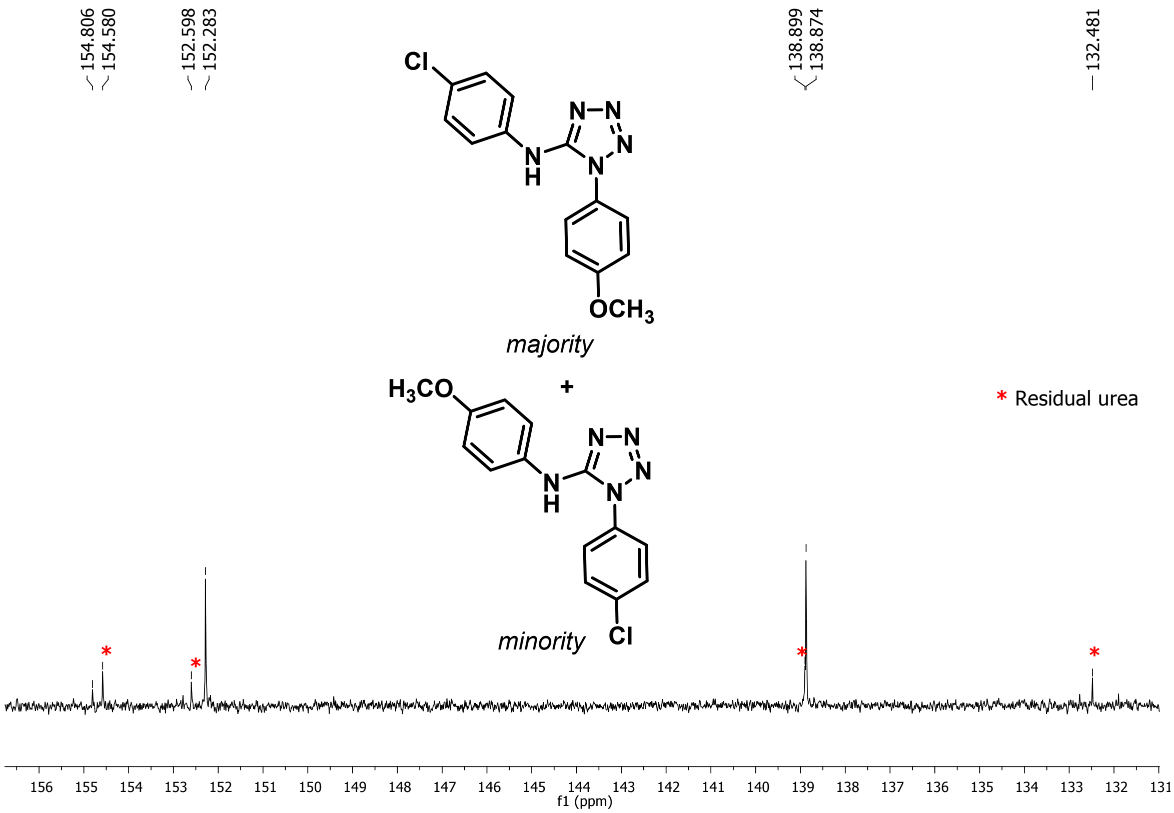

Expansion of $^{13}C\{^{1}H\}$ NMR (DMSO-d_6, 125 MHz) of 3r, 3r' and 13.

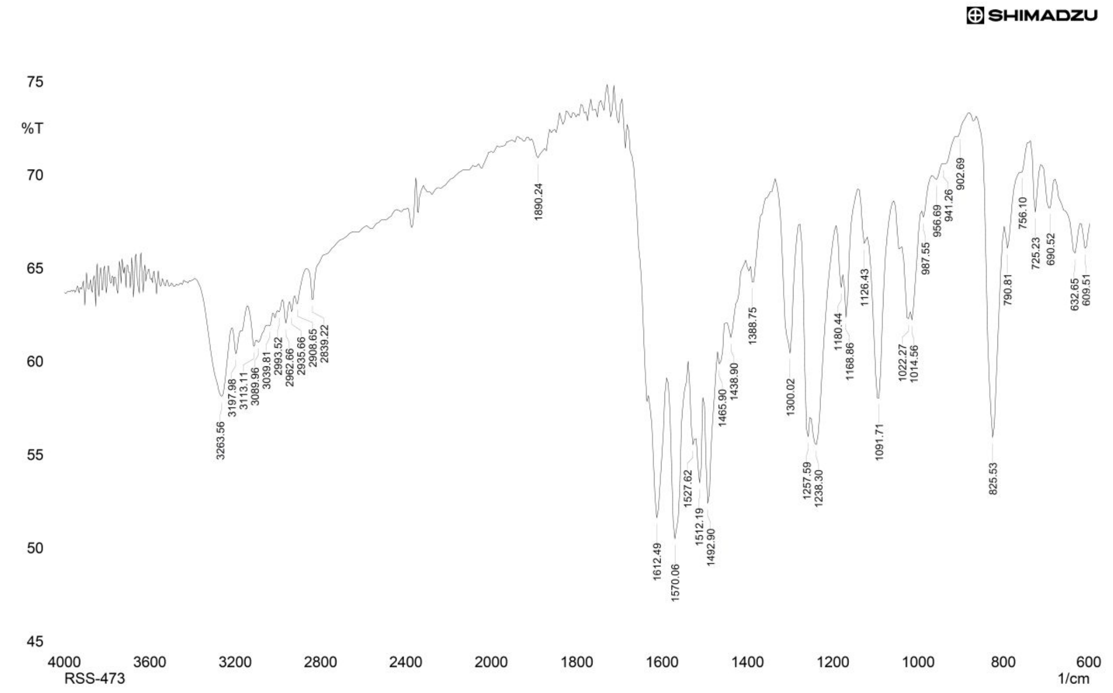

Comment;
RSS-473

No. of Scans; 20
Resolution; 8 [1/cm]
Apodization; Happ-Genzel

Date/Time; 04/07/2024 14:55:55
User; FTIR

IR spectrum (KBr) of 3r, 3r' and 13.

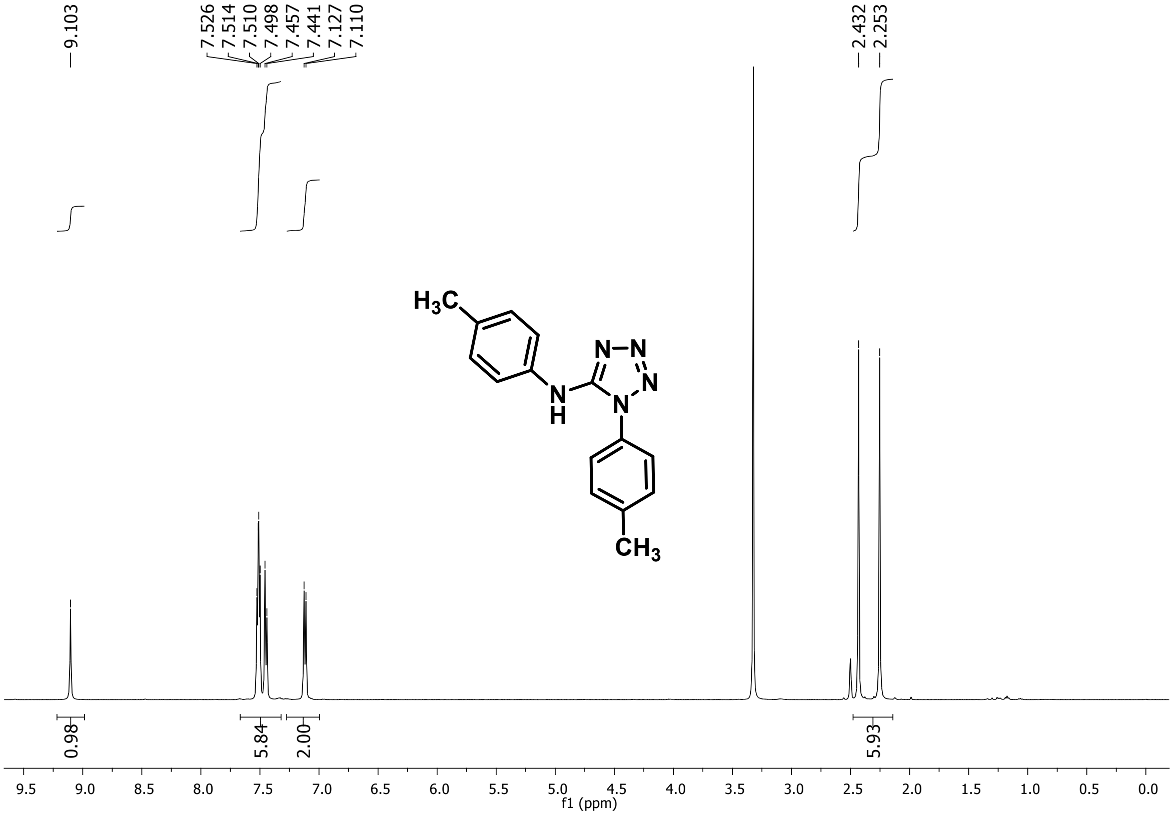

^{1}H NMR (DMSO-d_6, 500 MHz) of 3s.

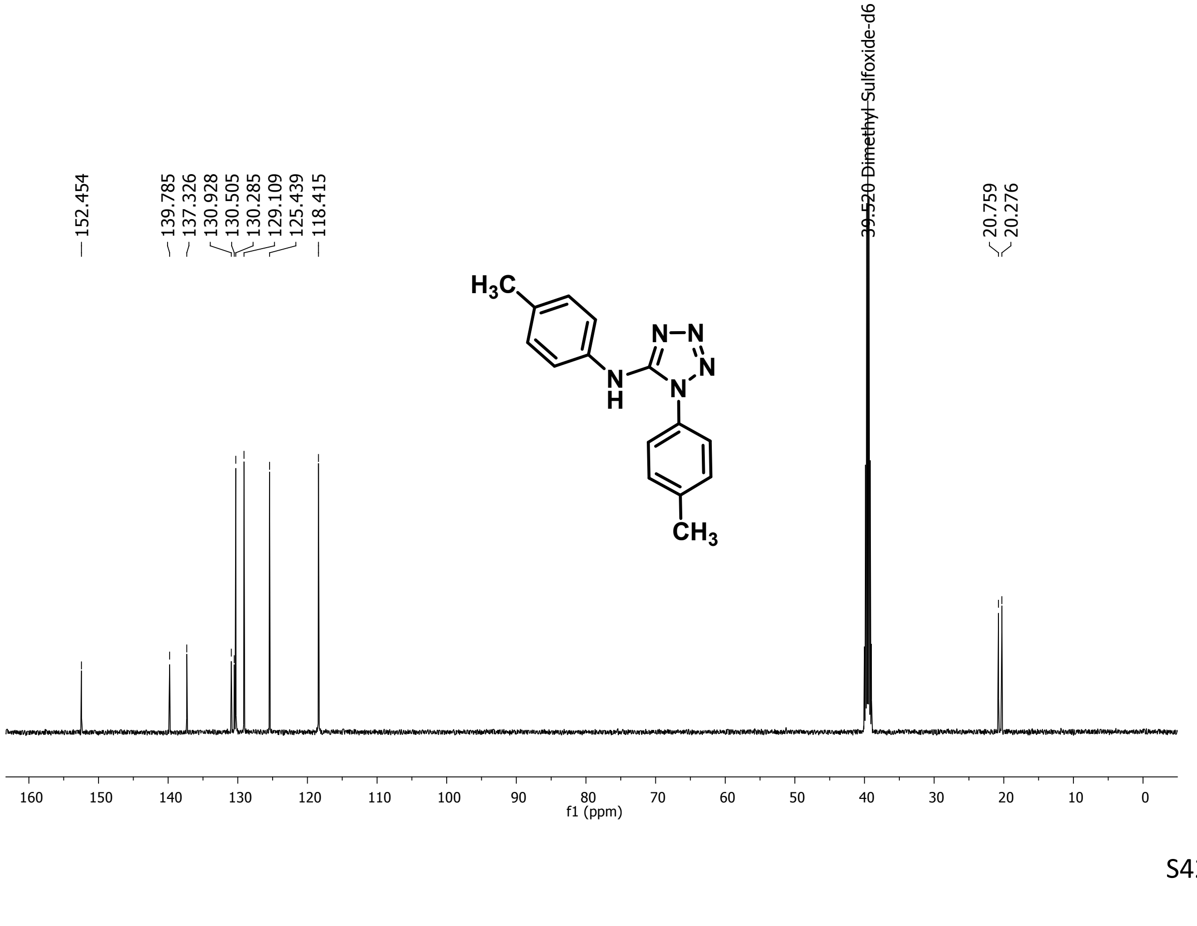

$^{13}C\{^1H\}$ NMR (DMSO-d_6, 125 MHz) of 3s.

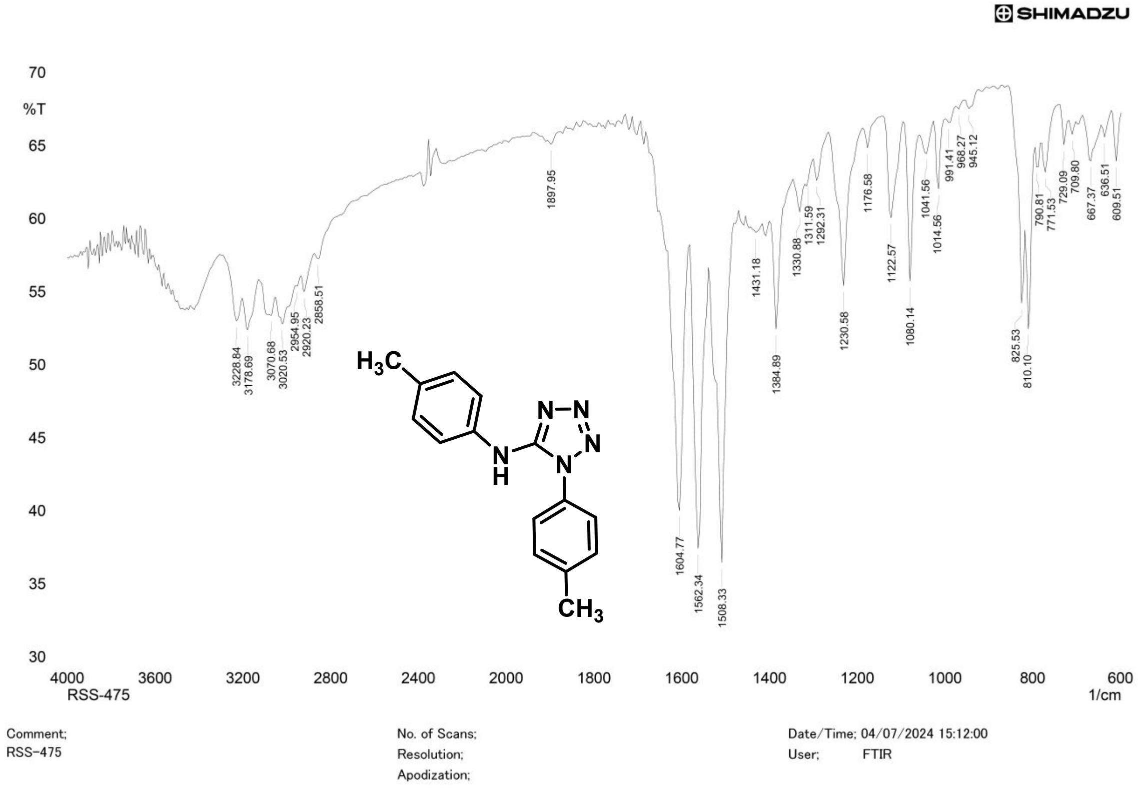

IR spectrum (KBr) of 3s.

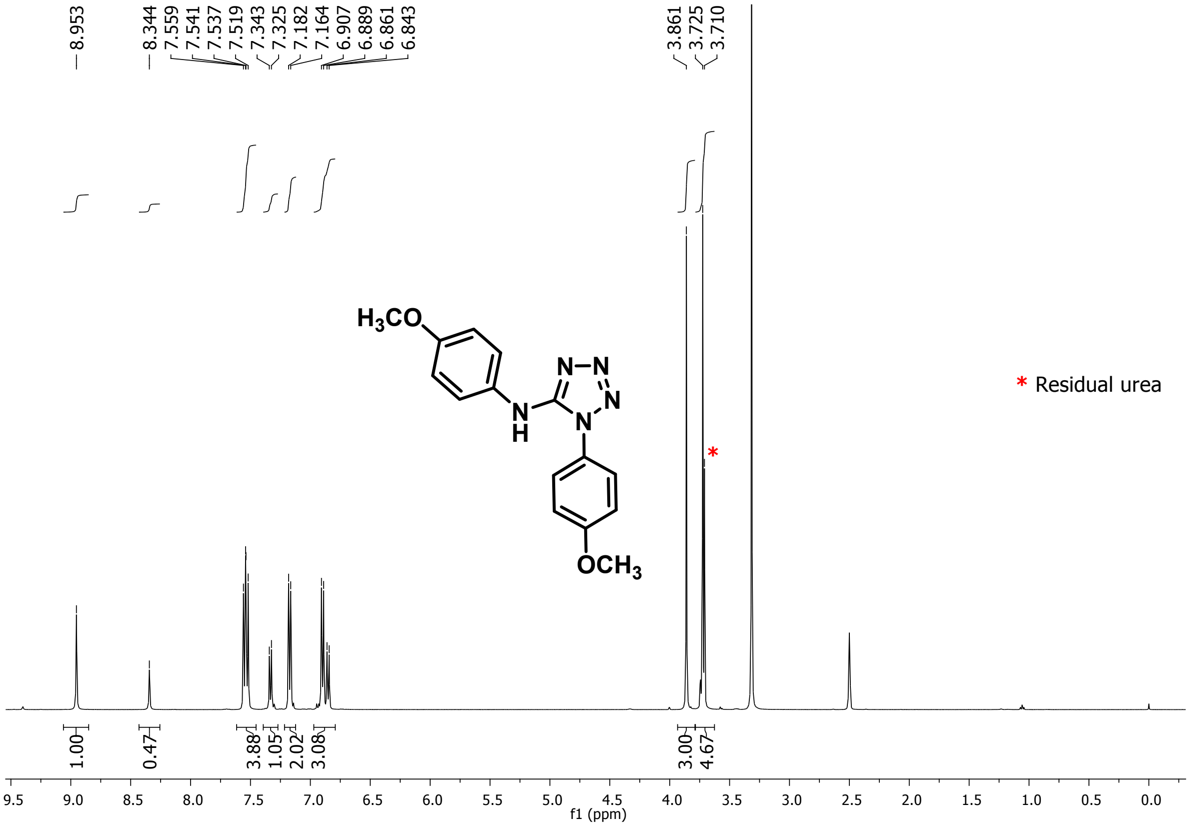

^{1}H NMR (DMSO-d_6, 500 MHz) of 3t and 14.

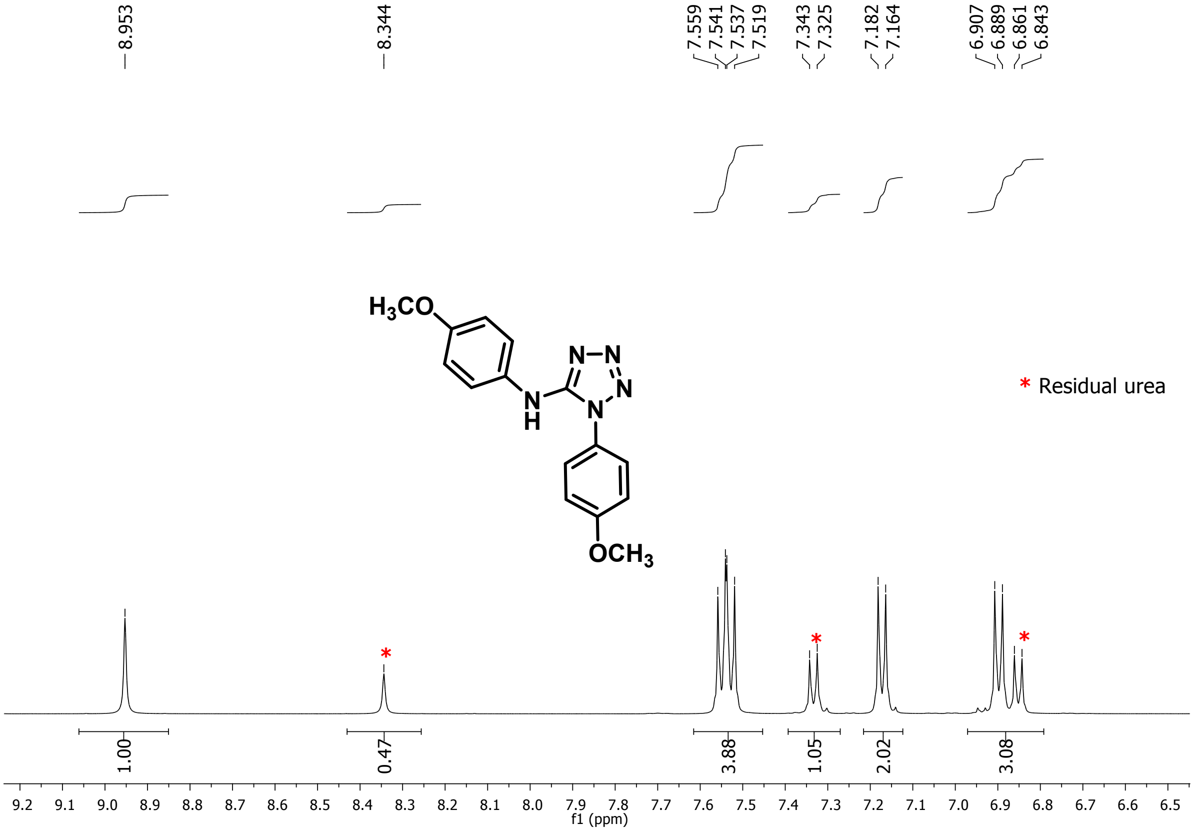

Expansion of ^{1}H NMR (DMSO-d_6, 500 MHz) of 3t and 14.

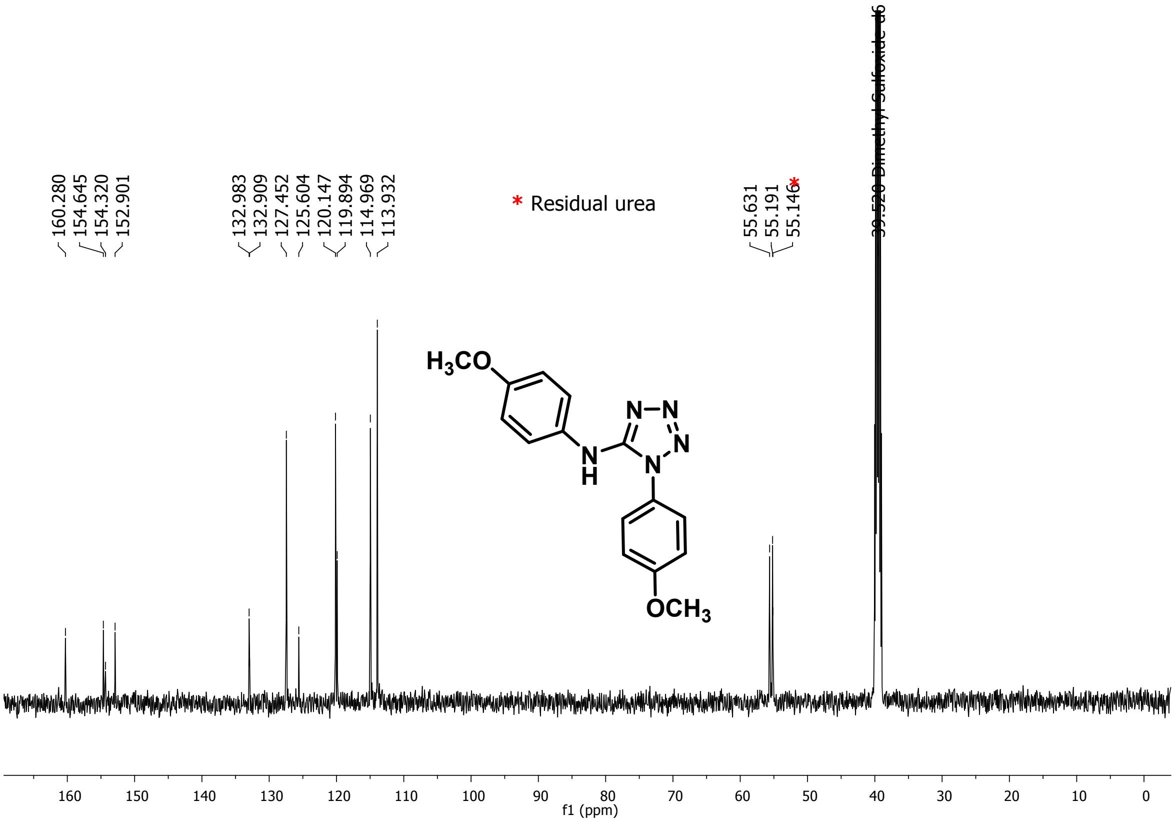

$^{13}C\{^1H\}$ NMR (DMSO-d_6, 125 MHz) of 3t and 14.

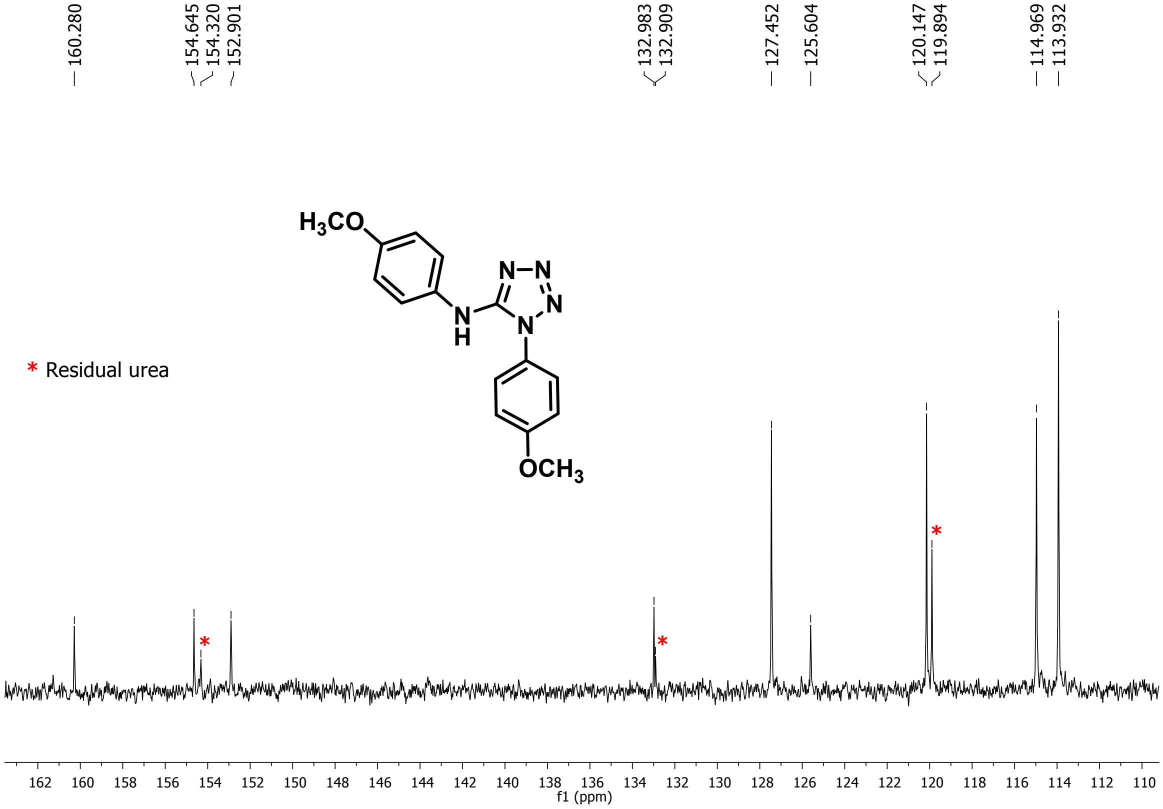

Expansion of $^{13}C\{^1H\}$ NMR (DMSO-d_6, 125 MHz) of 3t and 14.

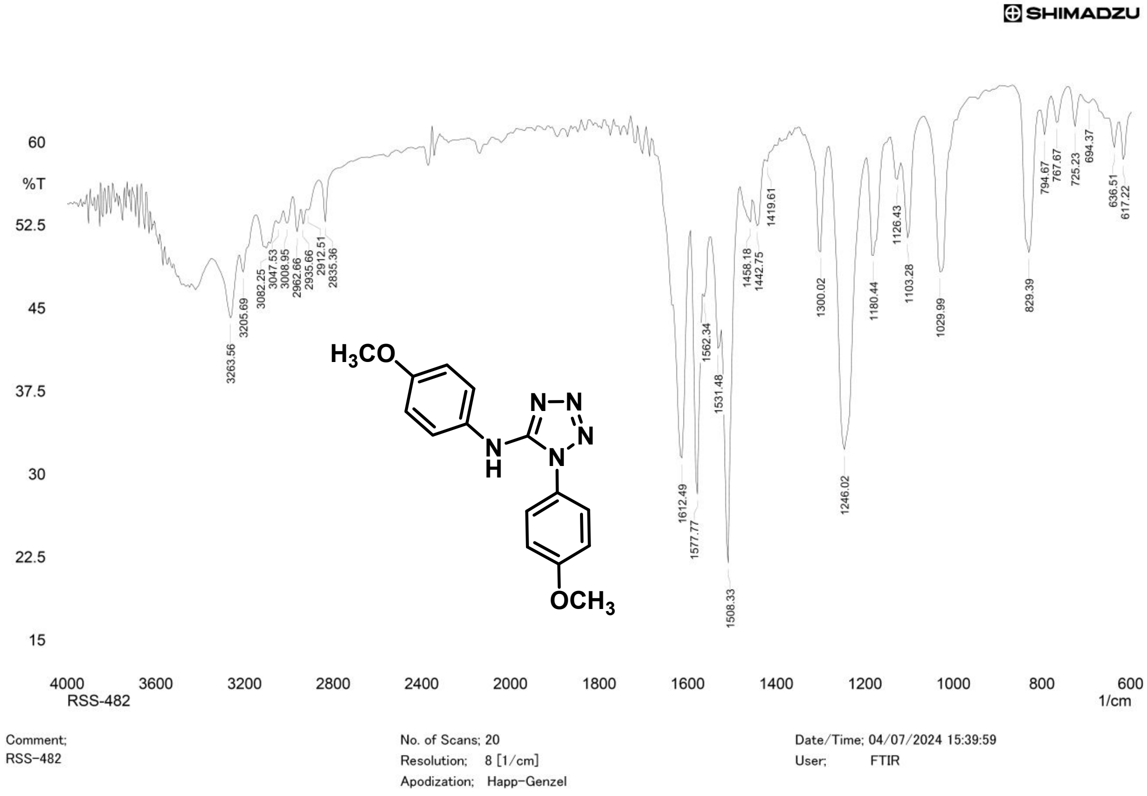

IR spectrum (KBr) of 3t and 14.

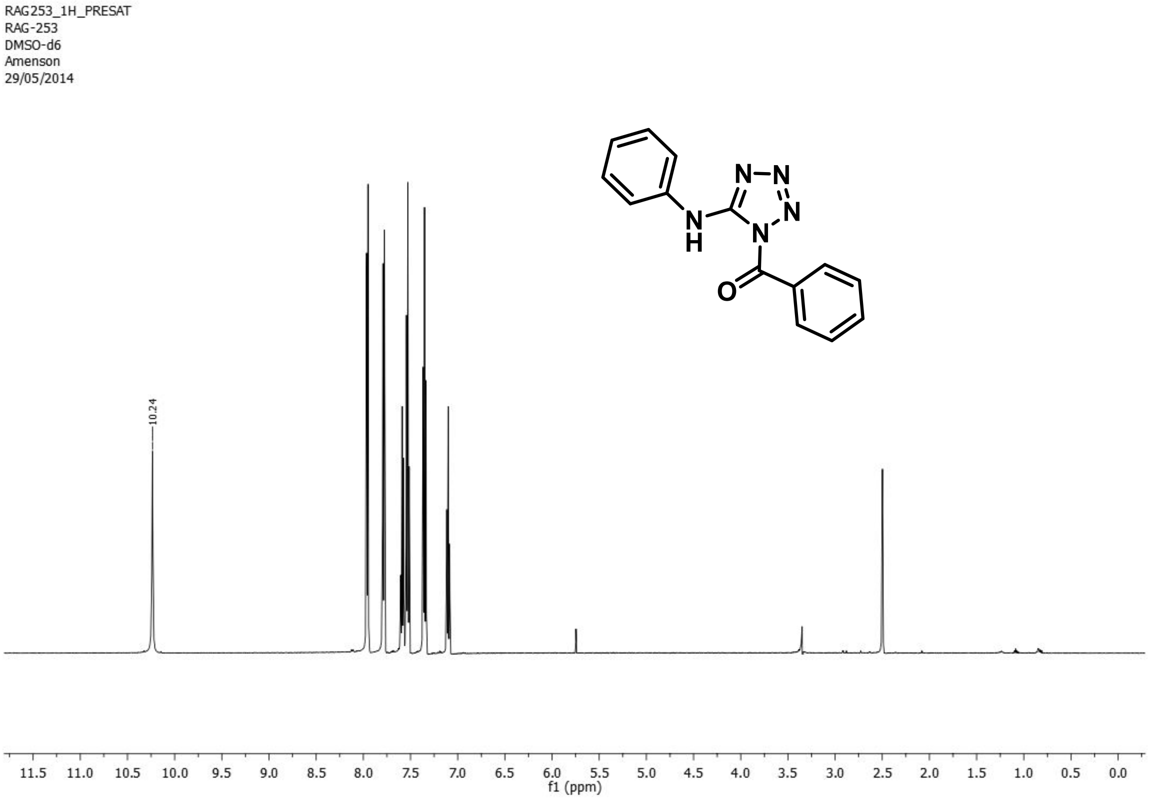

^{1}H NMR (DMSO-d_6, 500 MHz) of 11.

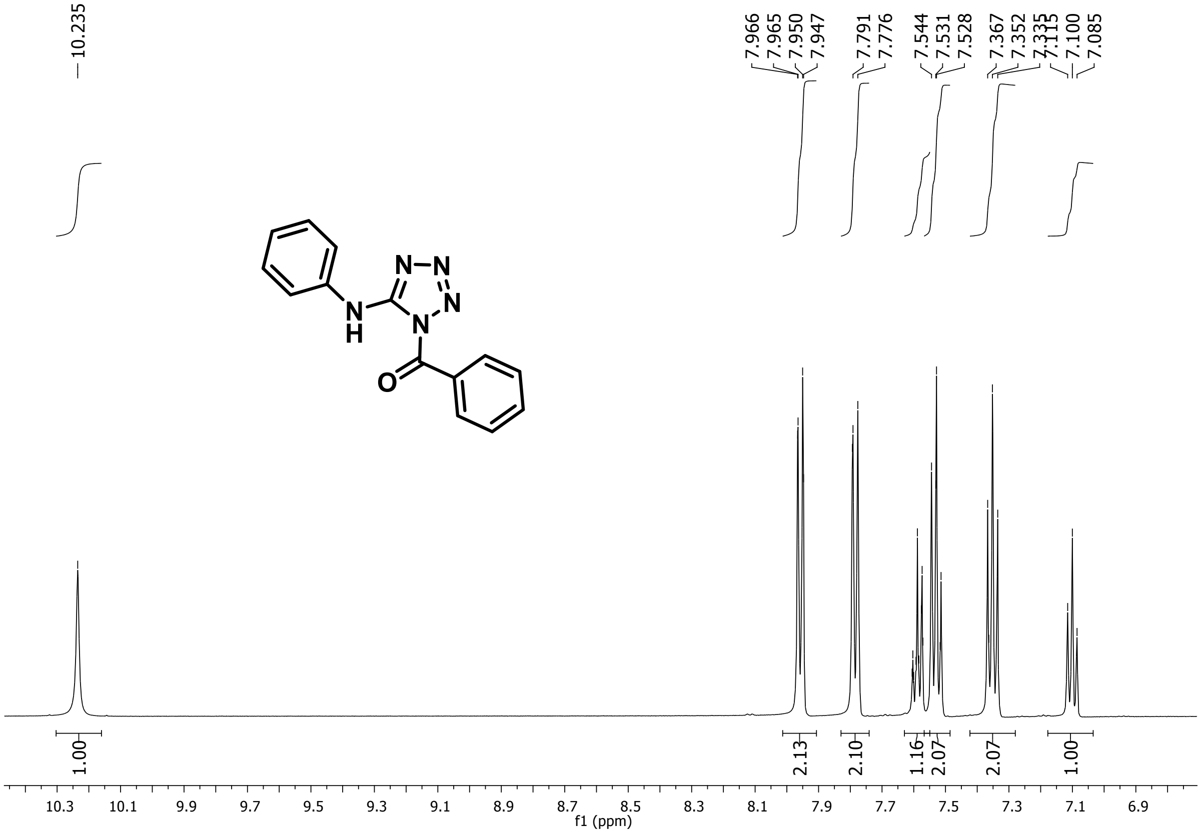

Expansion of ^{1}H NMR (DMSO-d_6, 500 MHz) of 11.

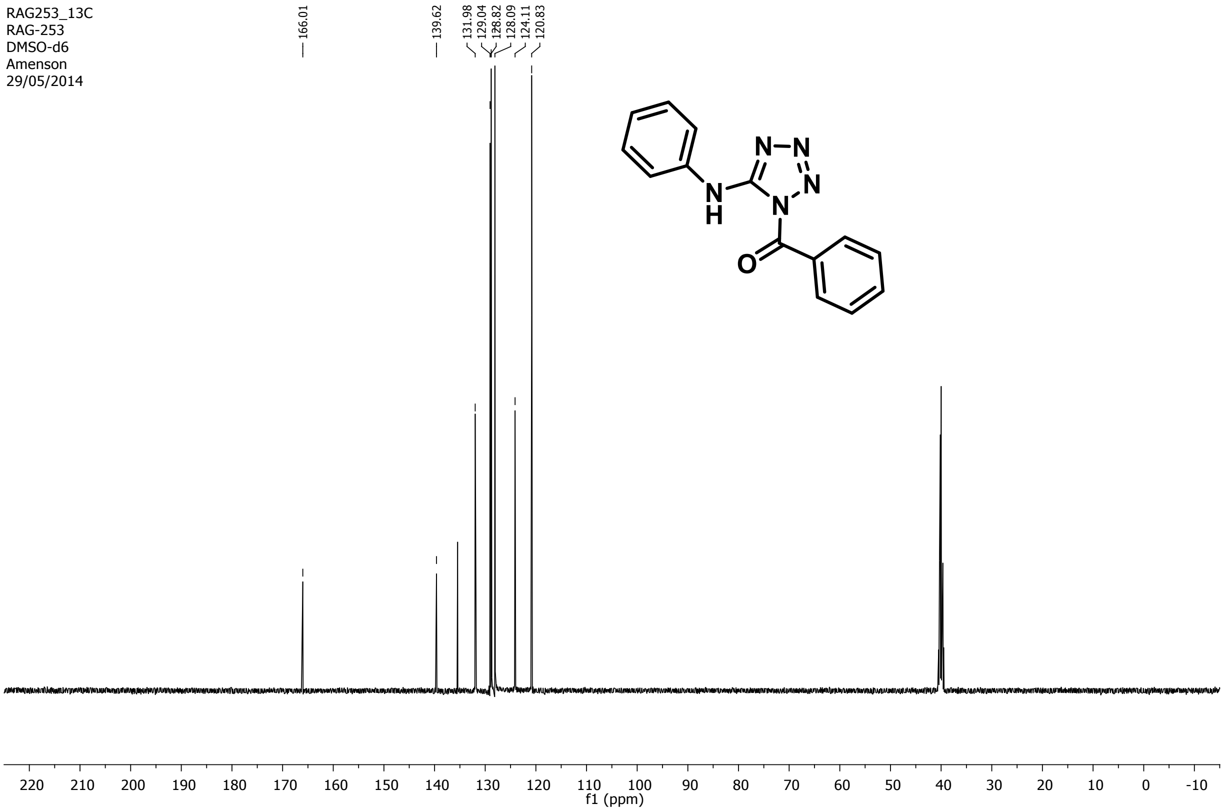

$^{13}C\{^{1}H\}$ NMR (DMSO-d_6, 125 MHz) of 11.

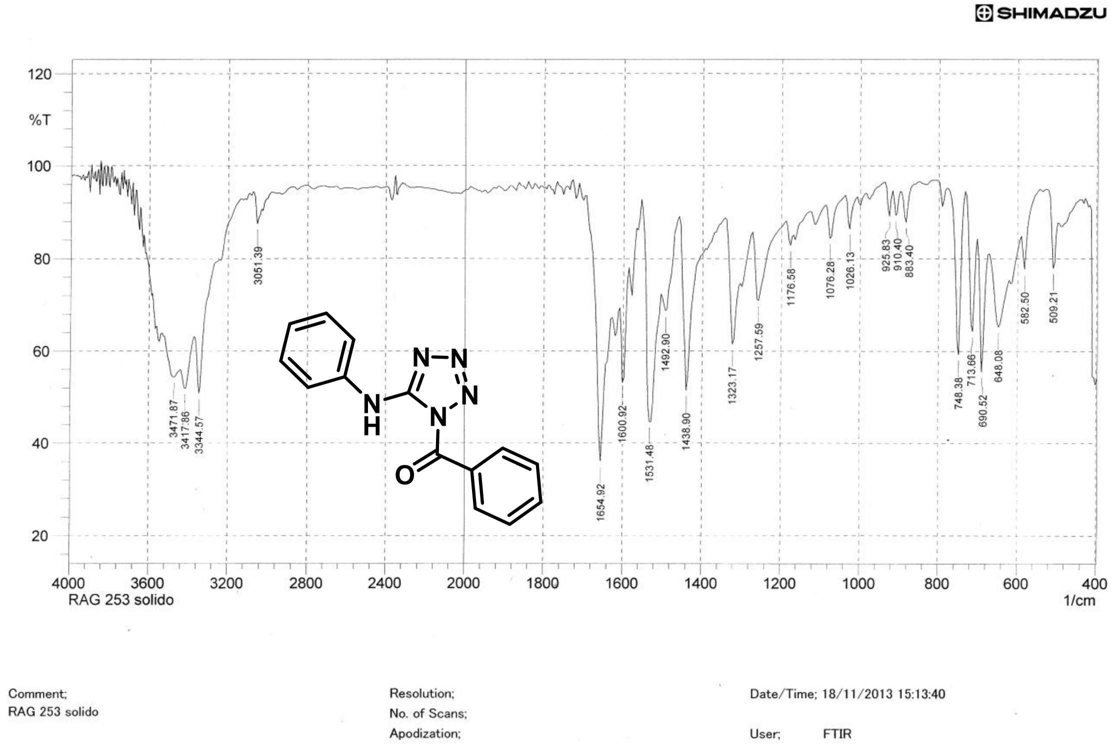

IR spectrum (KBr) of 11.

RAG 266 1H

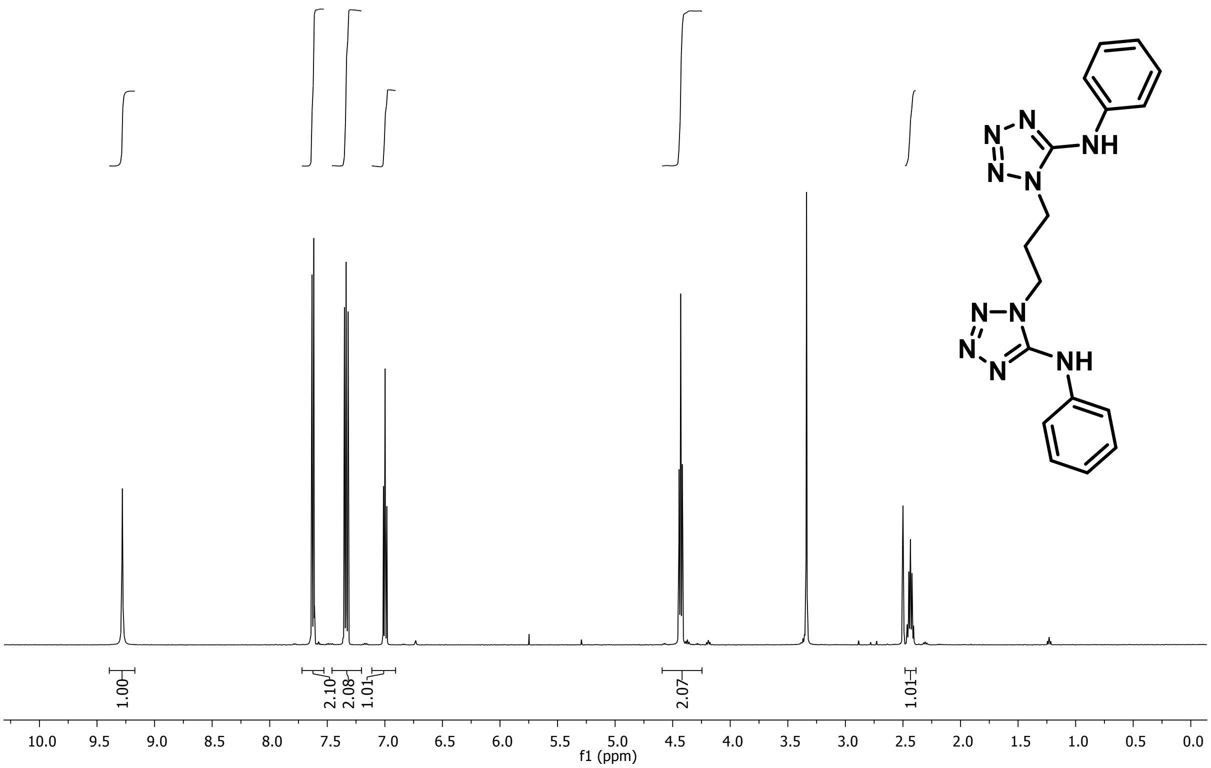

^{1}H NMR (DMSO-d_6, 500 MHz) of 5a.

RAG 266 13C

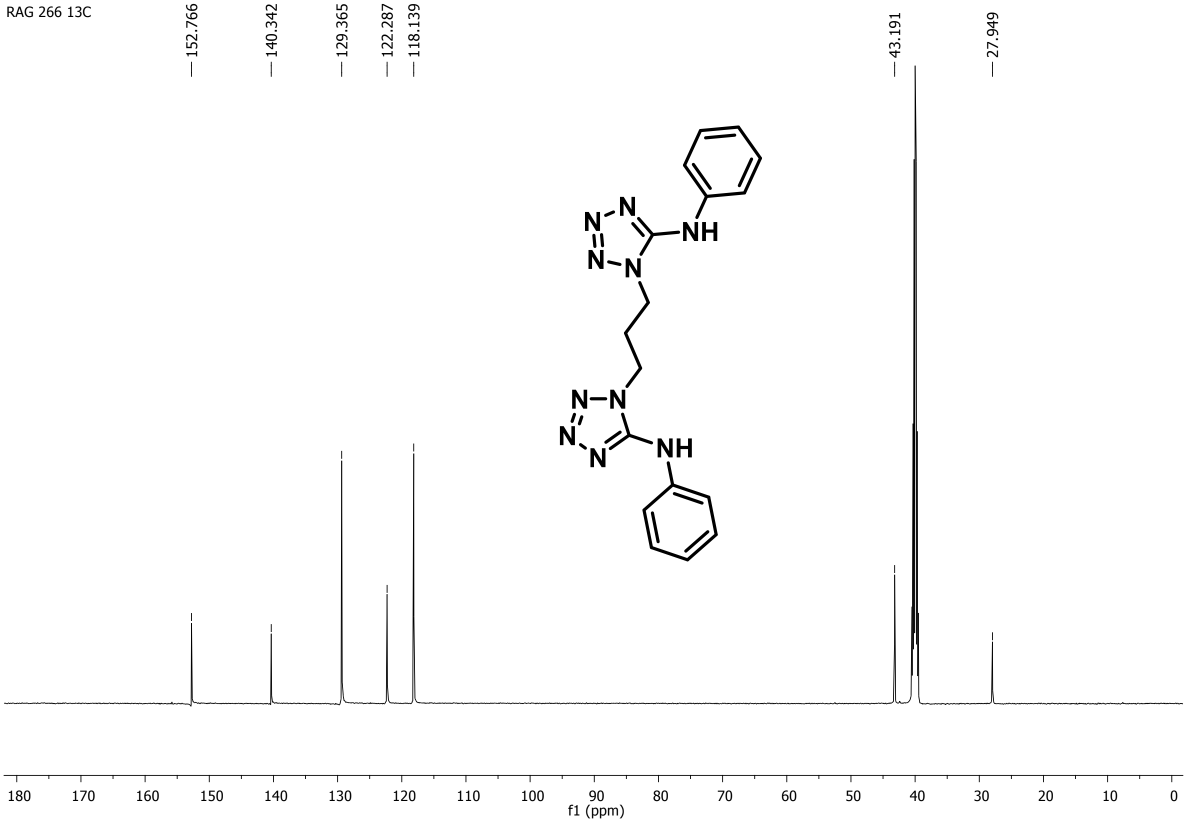

$^{13}C\{^1H\}$ *NMR (DMSO-d_6, 125 MHz) of 5a.*

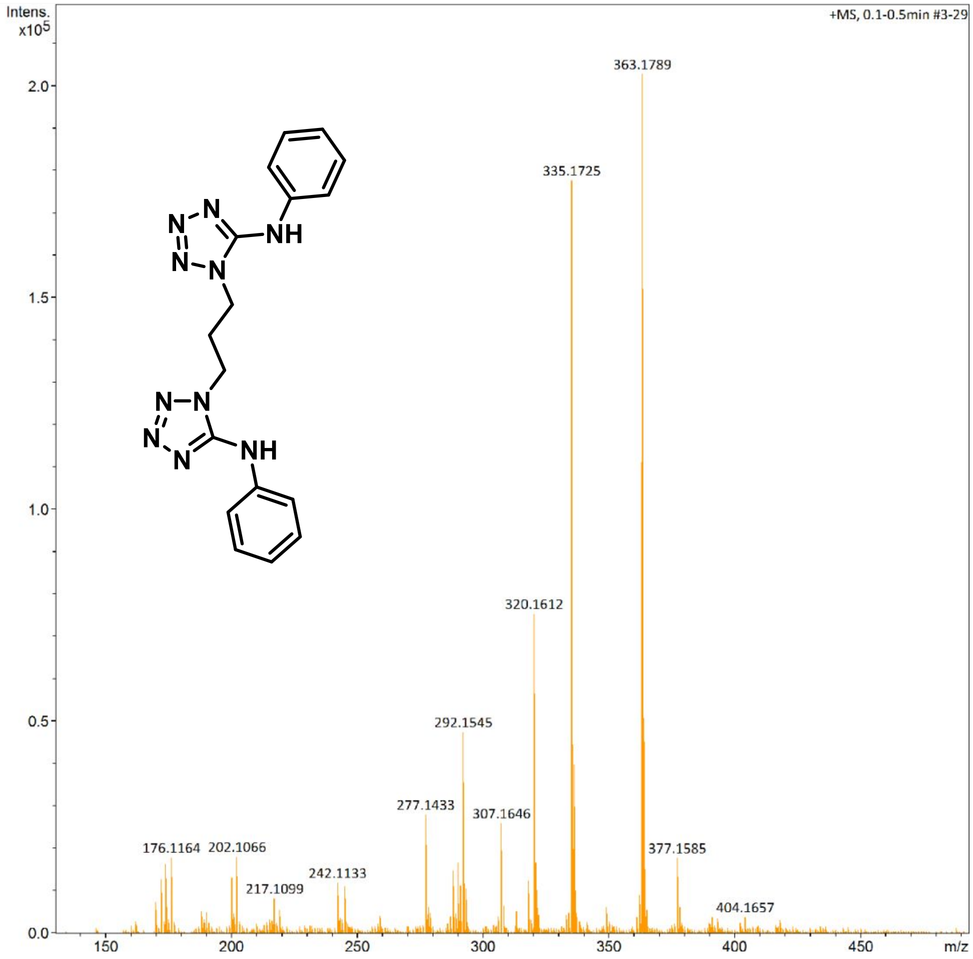

HRMS spectrum of 5a.

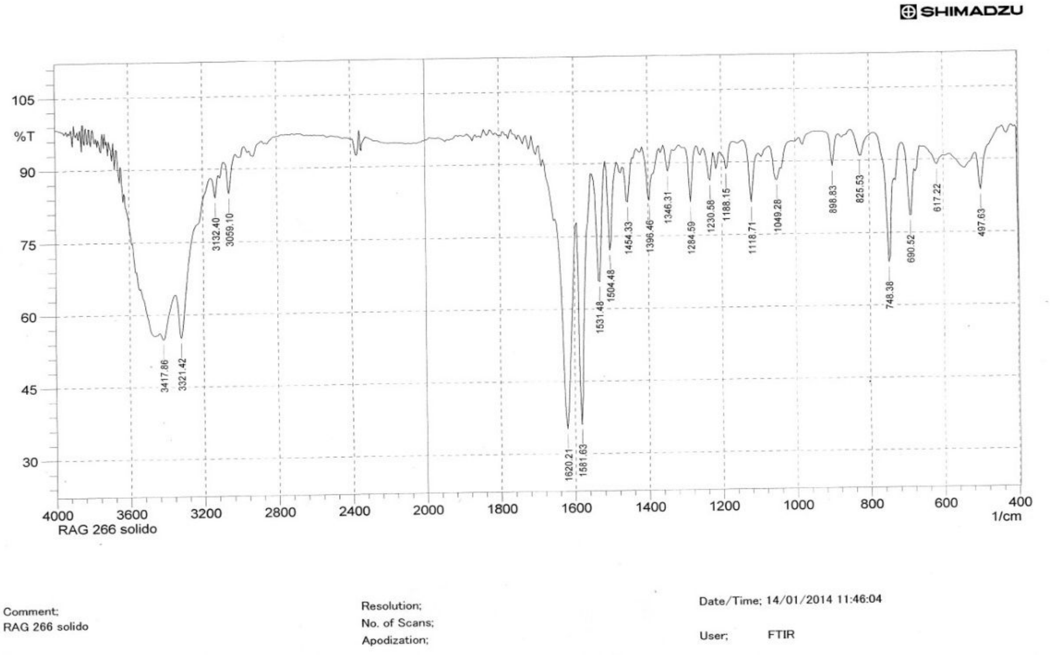

IR spectrum (KBr) of 5a.

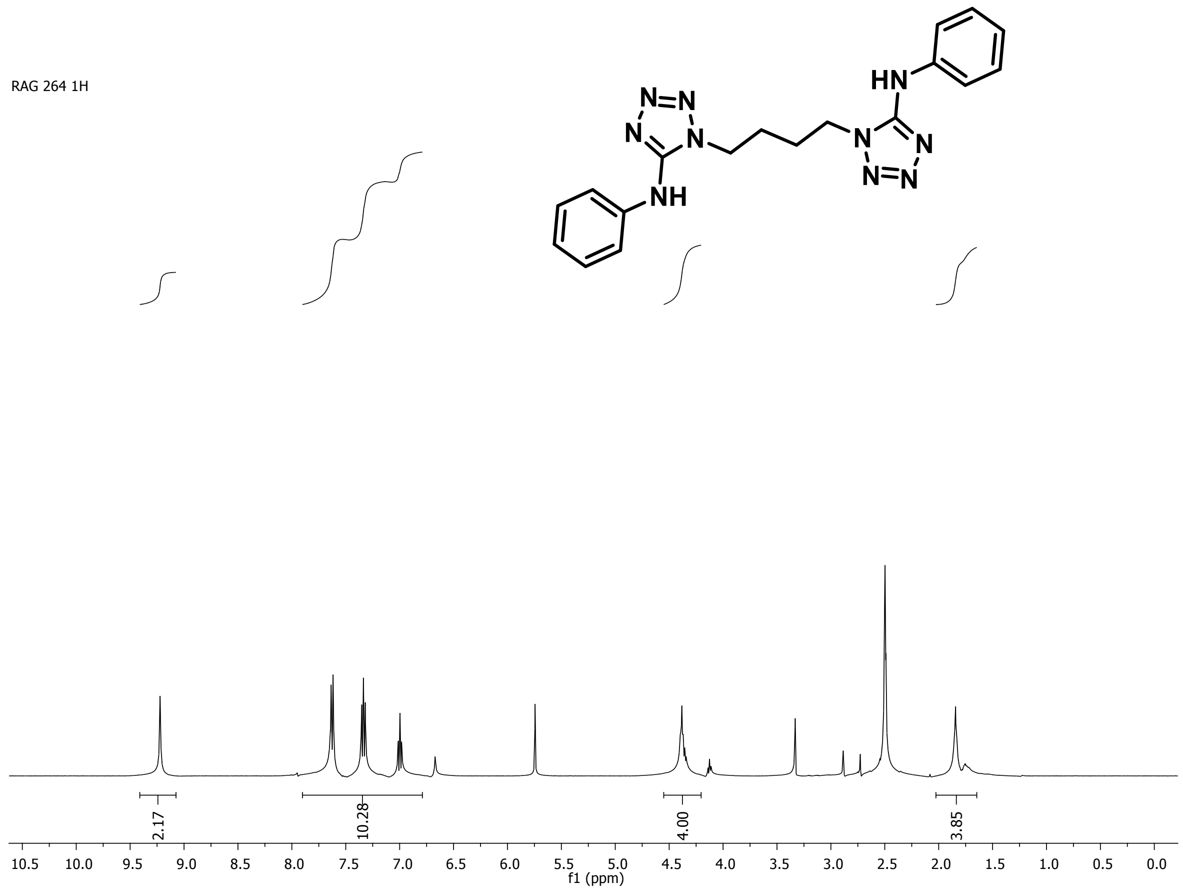

^{1}H NMR (DMSO-d_6, 500 MHz) of 5b.

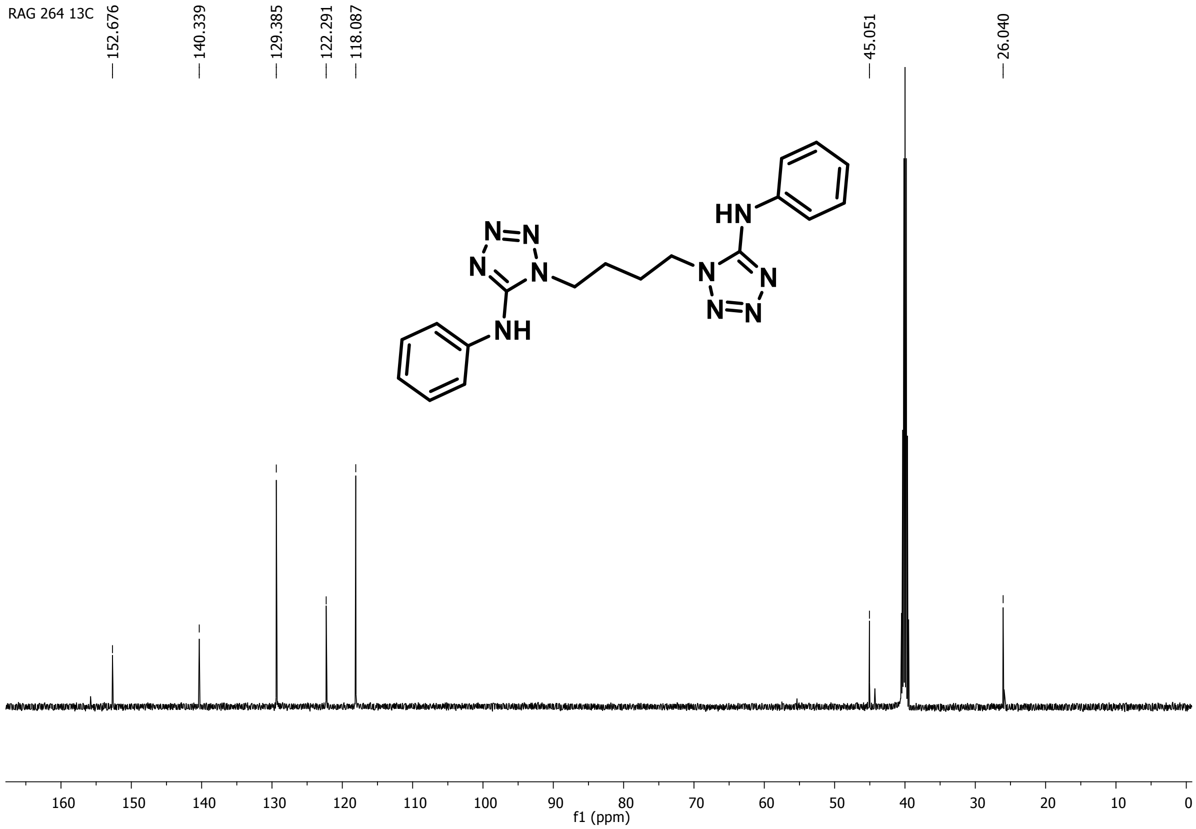

$^{13}C\{^{1}H\}$ NMR (DMSO-d_6, 125 MHz) of 5b.

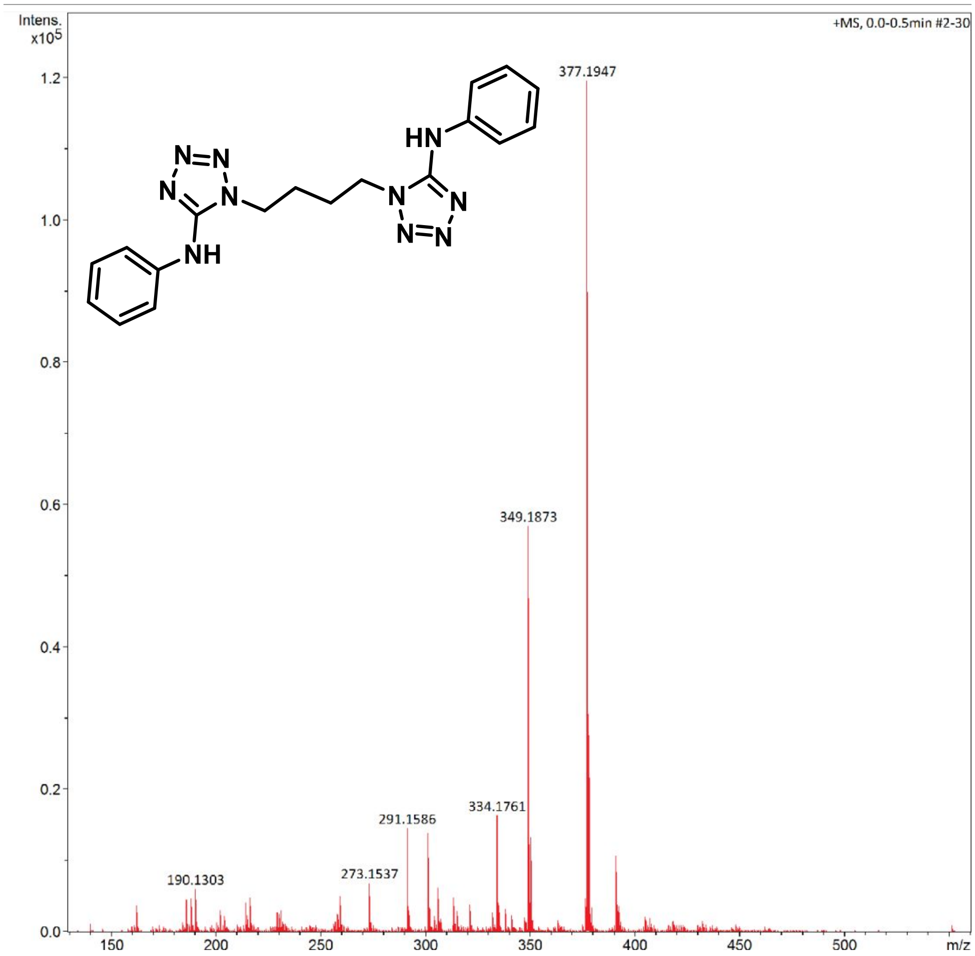

HRMS spectrum of 5b.

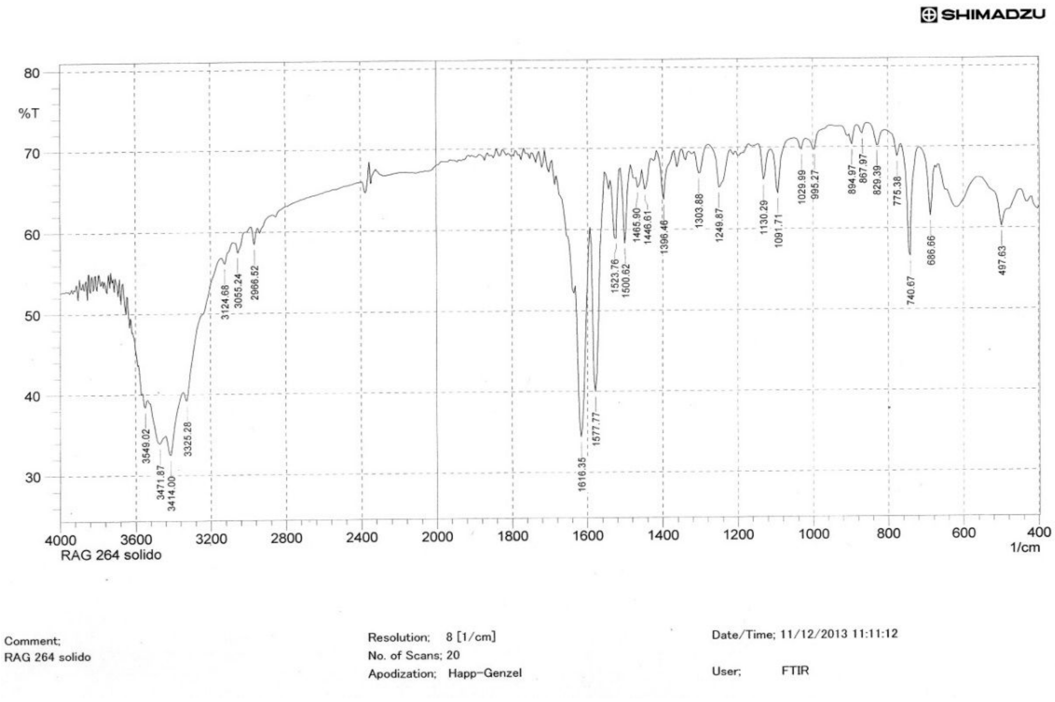

IR spectrum (KBr) of 5b.

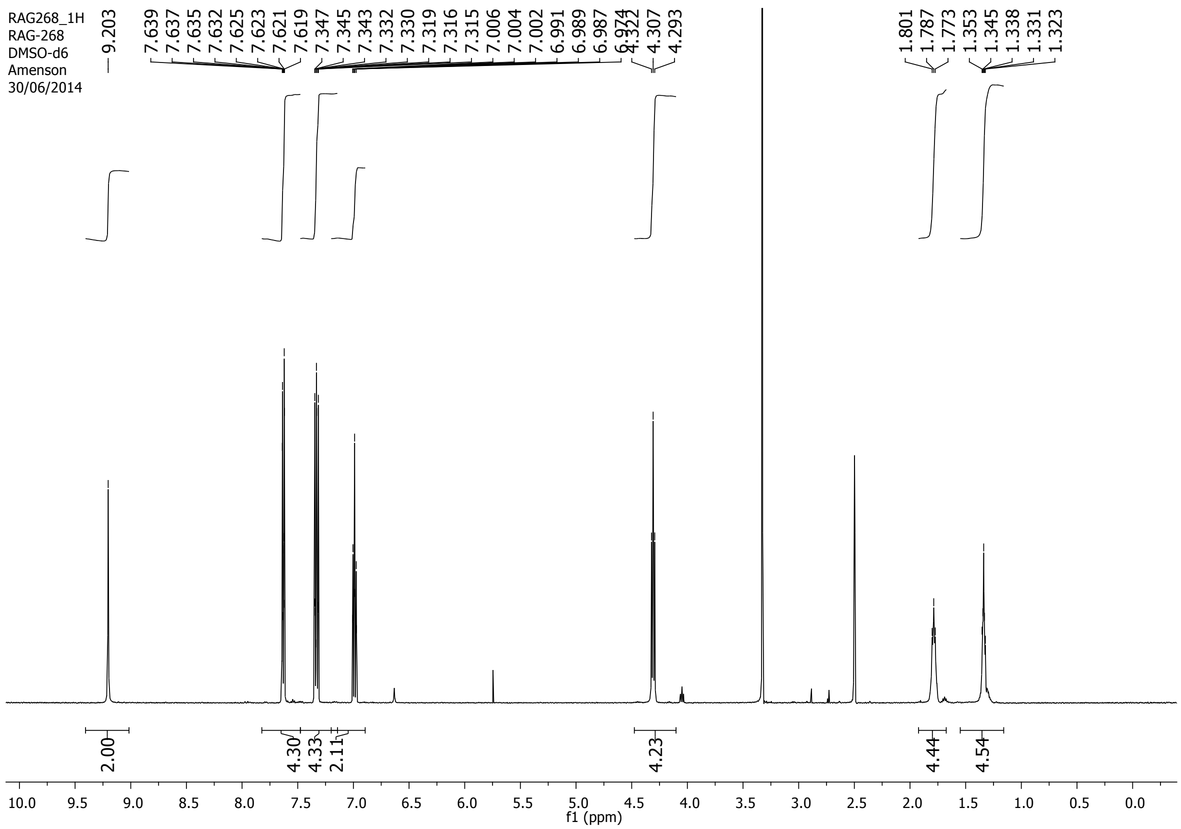

^{1}H NMR (DMSO-d_6, 500 MHz) of 5c.

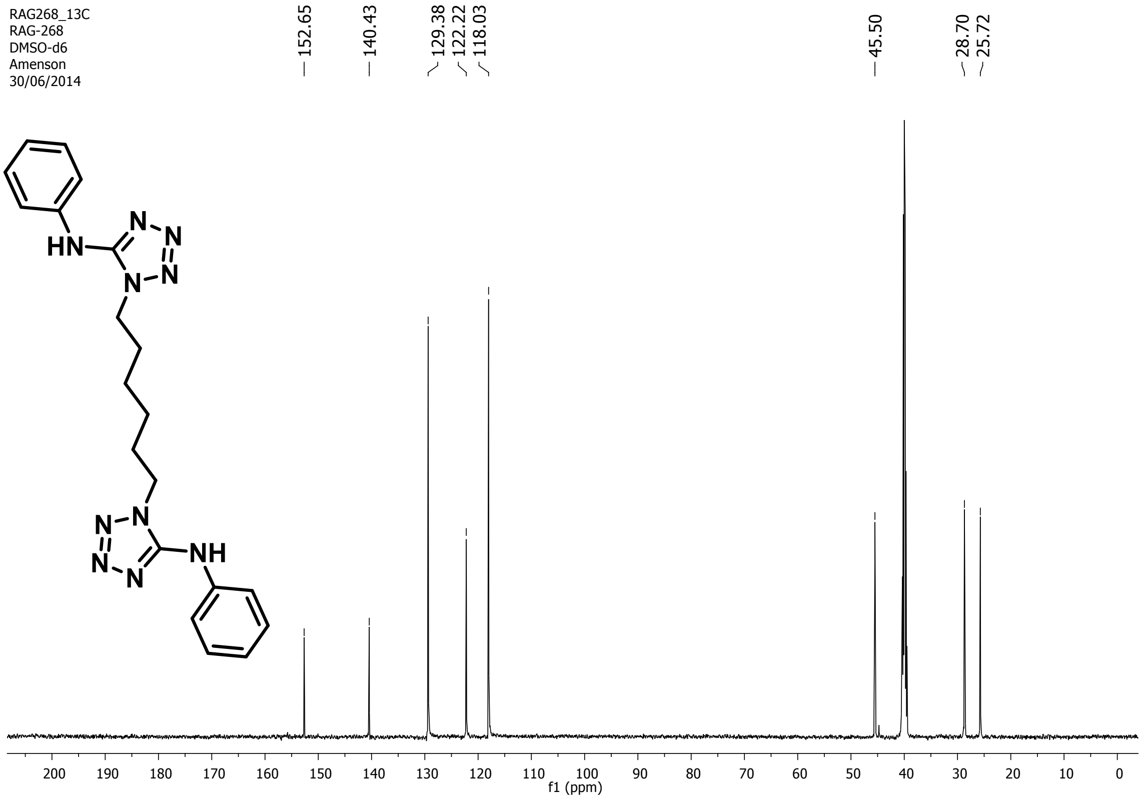

$^{13}C\{^{1}H\}$ NMR (DMSO-d_6, 125 MHz) of 5c.

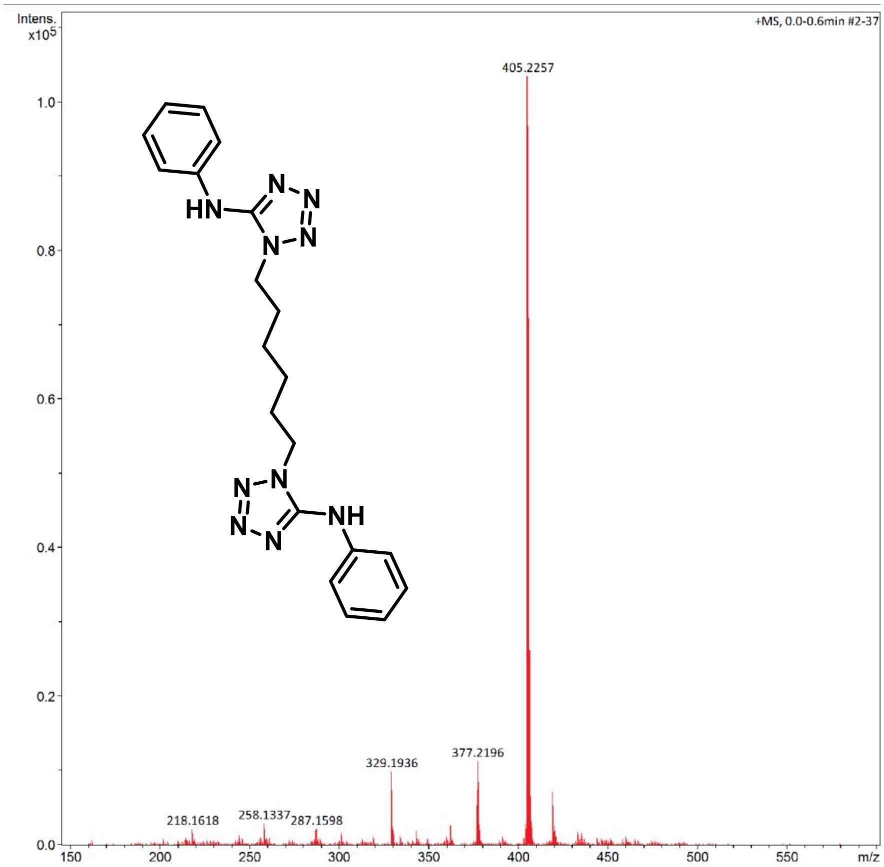

HRMS spectrum of 5c.

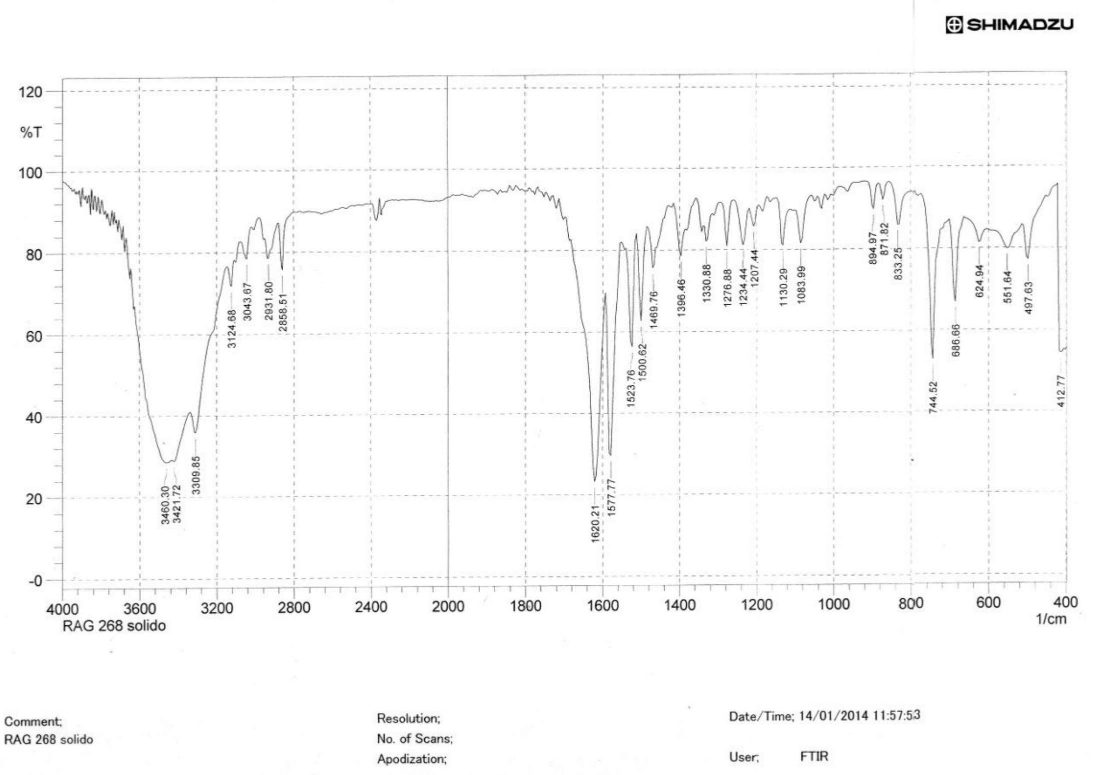

IR spectrum (KBr) of 5c.

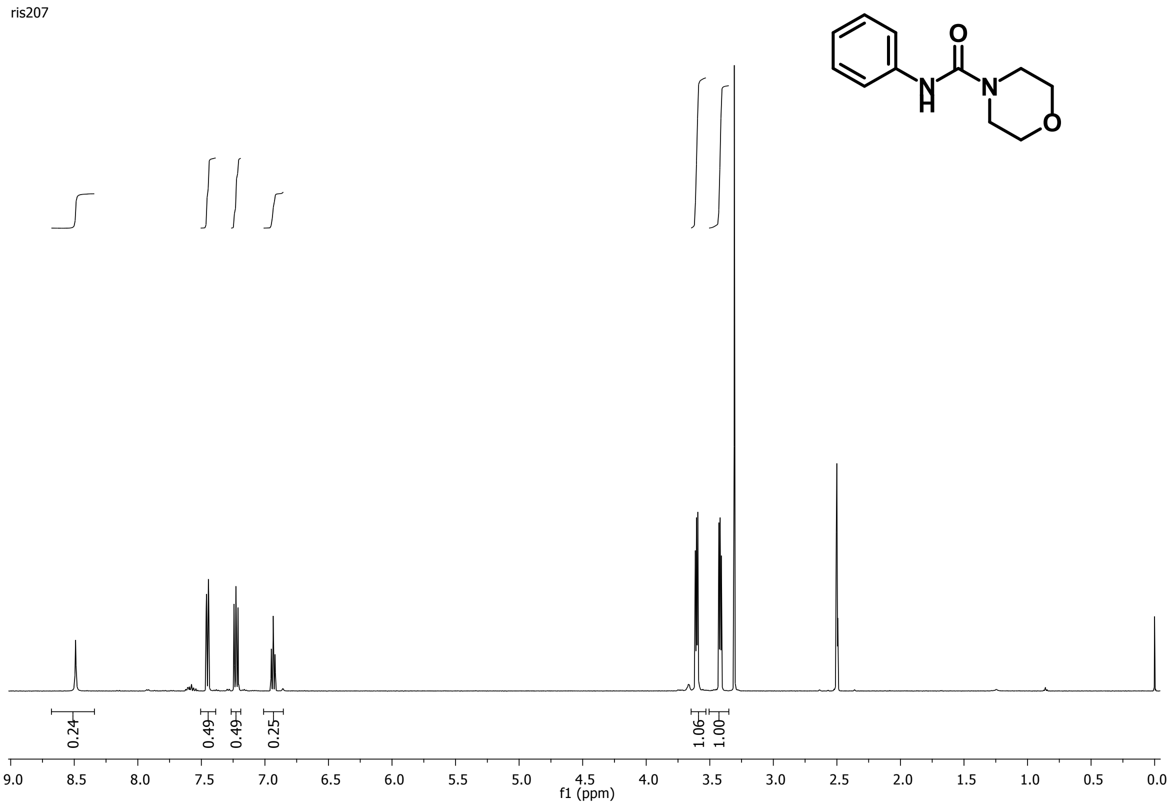

^{1}H NMR (DMSO-d_6, 500 MHz) of 9.

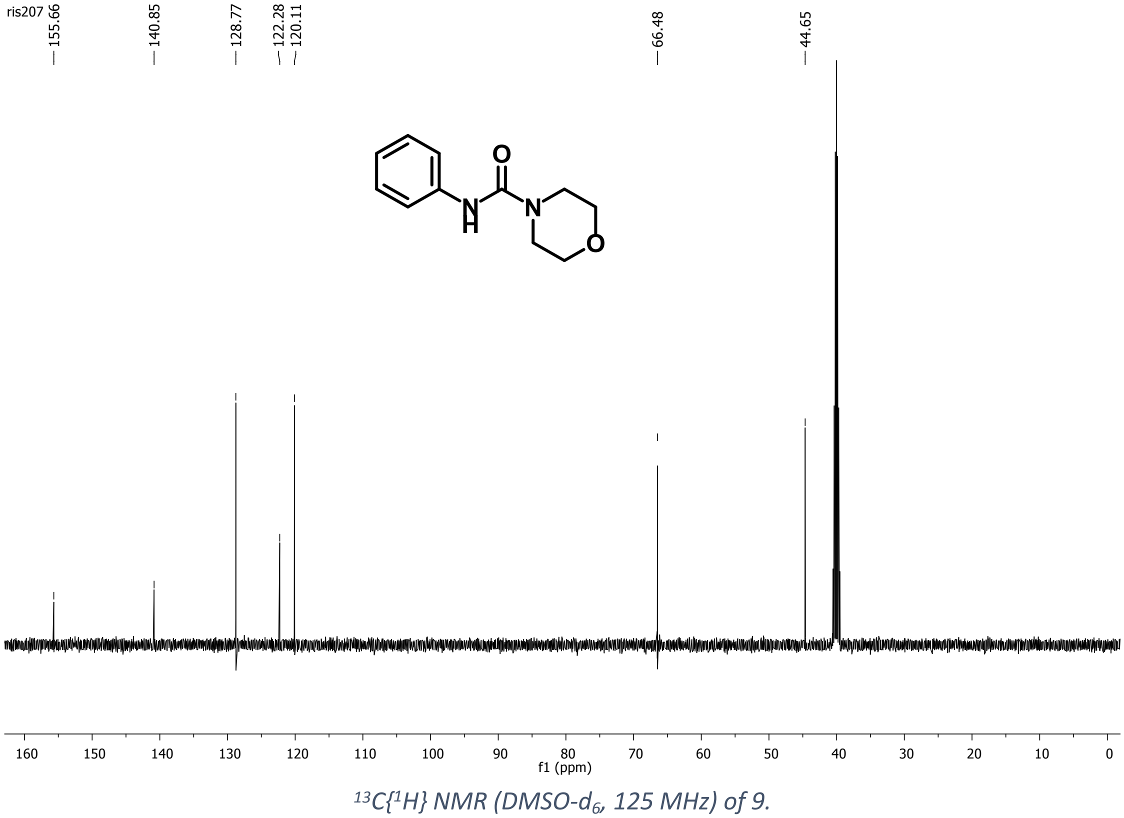

$^{13}C\{^{1}H\}$ NMR (DMSO-d_6, 125 MHz) of 9.
